# Population-level interventions for the primary prevention of dementia: a complex evidence review

**DOI:** 10.1016/j.eclinm.2024.102538

**Published:** 2024-03-10

**Authors:** Sebastian Walsh, Lindsay Wallace, Isla Kuhn, Oliver Mytton, Louise Lafortune, Wendy Wills, Naaheed Mukadam, Carol Brayne

**Affiliations:** a Cambridge Public Health, University of Cambridge, Cambridge CB2 0SR, UK; b University of Cambridge Medical School Library, School of Clinical Medicine, Cambridge CB2 0SP, UK; c Great Ormond Street Institute of Child Health, University College London, London WC1N 1EH, UK; d Centre for Research in Public Health and Community Care, University of Hertfordshire, Hatfield AL10 9AB, UK; e Division of Psychiatry, University College London, London W1T 7BN, UK

## Abstract

Dementia risk reduction is a global public health priority. Existing primary prevention approaches have favored individual-level interventions, with a research and policy gap for population-level interventions. We conducted a complex, multi-stage, evidence review to identify empirical evidence on population-level interventions for each of the modifiable risk factors identified by the Lancet Commission on dementia (2020). Through a comprehensive series of targeted searches, we identified 4604 articles, of which 135 met our inclusion criteria. We synthesized evidence from multiple sources, including existing non-communicable disease prevention frameworks, and graded the consistency and comprehensiveness of evidence. We derived a population-level intervention framework for dementia risk reduction, containing 26 high- and moderate-confidence policy recommendations, supported by relevant information on effect sizes, sources of evidence, contextual information, and implementation guidance. This review provides policymakers with the evidence they need, in a useable format, to address this critical public health policy gap.

**Funding:**

SW is funded by a 10.13039/501100000272National Institute for Health and Care Research (NIHR) Doctoral Fellowship. WW and LF are part funded by the 10.13039/501100000272NIHR Applied Research Collaboration East of England. The views expressed are those of the authors and not necessarily those of the NIHR or the Department of Health and Social Care.

## Introduction

With population ageing, dementia has emerged as a major public health challenge. Global prevalence is forecast to almost triple in the coming decades, to 150 million people living with dementia in 2050, mostly in low and middle income countries (LMICs).^1^ In addition to the human cost to people with dementia and their families, dementia also places significant burden onto health and social care systems, as well as the broader economy, with global societal costs estimated as $1313.4 billion in 2019.^2^

Evidence from high income countries suggests that dementia incidence can be reduced.^3^^,^^4^ Moreover, health behaviours such as eating a healthy diet, being physically active, and not smoking are associated with delaying onset of dementia by longer than the mortality benefit, meaning a ‘compression of morbidity’ and an associated reduction in disease costs.^5^^,^^6^

Syntheses of observational studies by the Lancet commission on dementia has identified 12 potentially modifiable lifecourse risk factors for dementia, collectively associated with around 40% of dementia prevalence (higher in LMICs^7^): low educational attainment, hearing loss, traumatic brain injury (TBI), hypertension, excess alcohol, obesity, tobacco smoking, depression, social isolation, physical inactivity, air pollution, and diabetes.^8^ Moving beyond observational data on risk factor associations, to interventional evidence measuring empirical effects on dementia prevalence is difficult because risk is accumulated across the lifecourse, pathology builds up over decades, and disease onset is often distal to cardiovascular events.^9^ Causality must therefore be considered on the balance of evidence. If causality is assumed, it is possible to focus the assessment of the evidence for dementia prevention policy on changes in the risk factors themselves, rather than empirically measuring changes in dementia prevalence itself.

Prevention of a disease by addressing its risk factors is known as ‘primary prevention’, which can be achieved by (i) ‘individual-level interventions’ – which target individuals with high risk profiles and encourage or support them to adopt healthier behaviours or receive clinical interventions; and (ii) ‘population-level interventions’ – which target the risk profile of the whole populations or communities by changing societal conditions.^10^ Population-level approaches have large potential effectiveness, economic, sustainability, and equity benefits over individual-level approaches,^9^, ^10^, ^11^, ^12^ but have been under-researched for dementia.^13^^,^^14^

The aim of this review is to summarize the best available evidence on which population-level interventions policymakers should consider adopting, to advance dementia prevention. It was not feasible to search the evidence bases of all 12 different risk factors through separate systematic reviews, and the production of a long, unfocused list of potentially effective interventions would be of limited utility to policymakers. Instead, we adopted a complex review approach^15^ in which we synthesize the evidence pragmatically and synergistically, identifying intervention themes, key contextual considerations, and implementation guidance, across each of the evidence bases of the 12 risk factors, to produce a population-level dementia risk reduction framework to guide policymakers.

## Methods

### Review structure

The review was conducted in four stages: (i) review of general non-communicable disease (NCD) prevention reviews; (ii) identification of population-level interventions for ‘typical NCD’ risk factors; (iii) identification of population-level interventions for ‘dementia-specific’ risk factors; and (iv) synthesis and production of a population-level dementia risk reduction framework. More detail on each stage is provided below.

### Review procedures

This review was registered on Prospero (ID:CRD42023396193).

All literature searches conducted in each review stage were developed and piloted with an expert medical librarian (IK). No language restrictions were applied. Full details of the search strategies are available in Appendix A.

In each of the review stages, article screening and selection was performed by two independent reviewers (SW and either LW or NM). Data extraction was by pre-agreed extraction template and was performed by SW and checked by LW. Reviewers (SW, NM and LW) met to discuss and resolve conflicts in selection and extraction at each review stage.

Throughout the review we considered population-level interventions to be: ‘measures applied to populations, groups, areas, jurisdictions, or institutions with the aim of changing the social, cultural, physical, commercial, economic, environmental, occupational, or legislative conditions to make them less conducive to the development or maintenance of the modifiable lifecourse risk factors for dementia, and/or more conducive to the development or maintenance of the modifiable lifecourse protective factors for dementia.^16^’

#### (i) Review of general NCD prevention literature

Significant efforts have already been undertaken to summarise the best interventions for the general prevention of NCDs.^17^ All of the 12 risk factors, as identified by the Lancet commission on dementia,^8^ are either NCD risk factors (low formal education, hearing loss, TBI, alcohol, obesity, smoking, social isolation, physical inactivity, and air pollution) or NCDs in their own right (diabetes, hypertension, depression). However, we considered that the general NCD prevention literature would be more mature with respect to some risk factors compared to others. We therefore conducted a rapid umbrella review of non-communicable disease prevention literature, to identify which risk factors were comprehensively included by this literature, and what types of population-level interventions were recommended.

On 12th of January 2023, and updated on 4th January 2024, we searched Medline via Ovid, Scopus, Web of Science, the Cochrane Library, and publications from the World Health Organization (WHO), and the UK National Institute for Health and Care Excellence (NICE), using terms for NCD, prevention, and review (Appendix A). We included reviews that were informed by systematic literature searches. In order to capture the generic NCD prevention literature, we excluded reviews that did not aim to summarise the totality of, or a significant proportion of, the NCD prevention literature (i.e., we excluded reviews with a narrow focus on a single disease). We excluded reviews in which none of the included interventional evidence met our definition of a population-level intervention. To capture an up-to-date reflection of the evidence base, we excluded reviews published more than a decade ago.

We assessed the risk of bias in the included reviews using the ‘A MeaSurement Tool to Assess systematic Reviews 2’ (AMSTAR 2) tool.^18^ We extracted data on risk factor(s) and/or disease(s) included, relative focus on population-level interventions, and types of population-level interventions considered to be effective.

#### (ii) Identification of interventions for typical NCD risk factors

Stage (i) identified six ‘typical NCD’ risk factors: smoking, alcohol, physical inactivity, obesity, hypertension, and diabetes (i.e., those that were consistently and comprehensively covered by existing NCD prevention literature; see Results section for more details), but the included reviews generally scored poorly on quality assessment and lacked detail on the interventions. We therefore conducted a focused search, on 22nd March 2023, for high-quality evidence summarising population-level interventions to address these six risk factors, drawing from two authoritative sources for the curation and weighing up of evidence to inform policy: the Cochrane Library, and the WHO. We searched the Cochrane library for reviews on any of the six typical NCD risk factors (Appendix A). For the WHO, we extracted data from Appendix 3 of the WHO's Global Action Plan for the Prevention and Control of NCDs,^19^ which summarizes the interventions considered in cost-effectiveness modelling by WHO for inclusion in the ‘Best Buys’^17^ report. This in turn draws interventions from regularly updated technical briefs for each of four risk factors (tobacco, smoking, alcohol, diet) and key diseases (including diabetes, and hypertension).

We included articles that summarised empirical estimates of the effects of population-level interventions for the prevention or control of any of the six typical NCD risk factors (we included calorie and sugar consumption as empirical outcomes for diet/obesity policy). We extracted description, study design, and findings (quantitative estimates of risk factor reduction) of the population-level intervention(s), assessments of the strength of evidence, and contextual information including study populations, equity effects, and implementation guidance.

#### (iii) Identification of interventions for dementia-specific risk factors

In order to summarize the evidence for population-level interventions to address the remaining six risk factors (education, hearing loss, TBI, depression, social isolation, and air pollution), in June 2023 we searched for publications in the Cochrane library, and from the WHO website using terms related to each of the risk factors. We also extracted any relevant data from the literature associated with appendix 3 of the WHO Global Action Plan. For depression and social isolation, we additionally searched the Campbell Collaboration library.

Although these searches identified relevant literature for all six dementia-specific risk factors, they yielded no suitable recommendations for three risk factors (education, depression, and social isolation). We therefore undertook further, more targeted, searches for these risk factors, designed to identify high-quality research which may have been excluded by the databases above. We searched the Turning Research into Practice (TRIP) database, Google Scholar, and via consultation with field experts. We consulted the types of interventions identified in stage (ii) to hypothesise similar interventions, which may be applicable to these three risk factors. The hypothesised interventions were used to structure the targeted searches (Appendix A).

Article inclusion, and data extraction, followed the same procedures as stage (ii).

#### (iv) Synthesis and derivation of a population-level intervention framework

Three authors (SW, LW and NM) met to apply confidence grades (low, medium, high) for the various interventions identified through stages (i) to (iii), according to the criteria in Box 1 which were developed specifically for this review. Owing to the nature of the interventions evaluated in the included articles, we considered evidence from a range of interventional and natural experiment study designs, and the inherent strengths and weaknesses of these designs were considered when appraising the strength of evidence.

Box 1 **Grading of confidence in recommended population-level interventions**

**Table:** 

High confidence recommendations	Interventions with comprehensive, consistent, and robust evidence, across a range of contexts, of a beneficial causal effect on the prevalence a risk factor
Moderate confidence recommendations	Interventions with clear and robust evidence of a beneficial causal effect on the prevalence of a risk factor, but only in a limited number of contexts
Low confidence recommendations	Interventions with empirical evidence demonstrating a clear signal that the intervention can reduce prevalence of a risk factor, but for which the evidence base lacks consistency or comprehensiveness

The recommended interventions were then compared against example NCD prevention frameworks identified in stages (i), (ii) or through professional networks, to identify intervention types which accurately grouped our recommendations, and to structure our own framework.

We then derived a ‘population-level dementia risk reduction intervention framework’, with columns grouping intervention types, rows listing risk factors, and populated only with recommendations judged at moderate or high confidence. To aid the use of the framework by policymakers, further relevant information was summarised, including example effect sizes, sources of evidence, key contextual information, whether evidence came from high-income countries (HIC) or LMICs, and implementation guidance. When selecting effect sizes to report, we used point estimates from meta-analyses where these were available, or the most conservative estimate from the highest-quality review or primary study, where meta-analysis was not available.

### Role of funding

The funders played no role in study design; in the collection, analysis, and interpretation of data; in the writing of the report; or in the decision to submit the paper for publication.

## Results

**Fig. 1:**
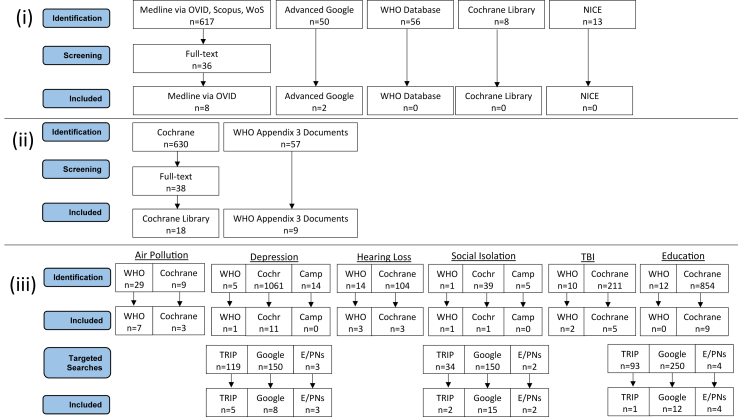
Modified PRISMA flowchart showing article identification and selection, by review stage. OVID = Interface for accessing Medline. WoS = Web of Science. NICE = National Institute of Health and Care Excellence. WHO = World Health Organization. Cochr = Cochrane Library. Camp = Campbell Collaboration. Google = Google Scholar. TRIP = Turning Research Into Practice. E/PNs = Experts and Professional Networks.

Across all stages of the review, we assessed 4604 articles for inclusion, and included 135 articles (Fig. 1). The included articles described interventions against multiple risk factors (n = 12), tobacco smoking (n = 9), excess alcohol (n = 2), obesity (n = 6), physical inactivity (n = 3), hypertension (n = 3), air pollution (n = 10), depression (n = 28), hearing impairment (n = 6), social isolation (n = 21), TBI (n = 7), and low education (n = 26) (Fig. 1).

We first describe the findings of each stage of the review, and then outline the findings for each risk factor in turn.

### (i) Review of general NCD prevention literature

Stage (i) of the review identified ten reviews (Fig. 1), of which nine were rated as being of ‘critically low’ quality, and one was rated as ‘low’ (Supplementary Table 1A). As such we were unable to rely heavily on the recommendations in these reviews, several of which were scoping-type documents owing to their broad view of the NCD prevention literature. Instead, we used their findings (Supplementary Table 1B) as supportive evidence for the interpretation of evidence found in later review stages. In addition, we identified that the evidence base would be most mature for six risk factors: tobacco smoking, excess alcohol, physical inactivity, obesity (which would often be considered through a lens of poor diet, from a primary prevention perspective), hypertension, and diabetes – which together we considered as ‘typical NCD risk factors’; and that more specific searches would likely be needed for the remaining six risk factors (hereafter named the ‘dementia-specific’ risk factors).

### (ii) Identification of interventions for typical NCD risk factors

We identified 18 cochrane reviews (Fig. 1) reporting evaluations of population-level interventions for typical NCD risk factors: tobacco smoking (n = 8), alcohol (n = 1), obesity (n = 5), physical inactivity (n = 2), and hypertension (n = 2) (Supplementary Table 2A).

The WHO's Global Action Plan for the prevention and control of NCDs was published in 2013. Appendix 3 of this report detailed key recommended interventions for four risk factors (smoking, alcohol, poor diet, physical inactivity) and four diseases (including cardiovascular disease, with some direct relevance to hypertension). The Appendix 3 document was then updated in 2017, in order to produce the 'Best buys and other recommended interventions for the prevention and control of NCDs' report. The WHO are currently in the process of updating the appendix 3 document for a second time, with the most recent draft published in August 2022. We extracted the 2013, 2017, and 2022 versions of appendix 3, along with the most recently updated technical annex (December 2022), and the latest accompanying technical briefs for each risk factor and for cardiovascular disease (November 2022) (Supplementary Table 2B).

### (iii) Identification of interventions for dementia-specific risk factors

We identified 32 Cochrane reviews (Fig. 1) reporting evaluations of population-level interventions for dementia-specific risk factors: air pollution (n = 3), depression (n = 11), hearing loss (n = 3), social isolation (n = 1), TBI (n = 5), and education (n = 9). We identified 14 WHO publications (Fig. 1) for: air pollution (n = 7), depression (n = 1), hearing loss (n = 3), TBI (n = 2), and social isolation (n = 1). We identified no relevant publications from the Campbell Collaboration searches (Fig. 1). Extracted evidence of population-level interventions is reported in Supplementary Table 3A.

The targeted searches yielded further results for depression (n = 16), social isolation (n = 19), and education (n = 17) (Fig. 1), and the extracted intervention data is reported in Supplementary Table 3B–D, respectively.

### (iv) Synthesis and derivation of a population-level intervention framework

Table 1 shows the grading of evidence for each recommendation, by risk factor. In total, we identified 11 high-confidence recommendations, 15 moderate-confidence recommendations, and 11 low-confidence recommendations, covering 10 of the risk factors (with no recommendations for diabetes or social isolation).

**Table 1:** Grading of evidence.

Risk factor	Intervention	Summary of evidence	Grade
Tobacco smoking	(T1) Increase excise taxes and prices on tobacco products	Consistent evidence from across several reviews	High
	(T2) Implement plain/standardized packaging and/or large graphic health warnings on all tobacco packages	Consistent evidence from across several reviews	High
	(T3) Enact and enforce comprehensive bans on tobacco advertising, promotion and sponsorship	Consistent evidence from across several reviews and articles	High
	(T4) Eliminate exposure to second-hand tobacco smoke in all indoor workplaces, public places, public transport	Consistent evidence across several publications/reviews, from HICs and LMICs	High
	(T5) Establish and enforce an appropriate minimum age for purchase or consumption of tobacco	A 2005 Cochrane review demonstrates effective policies for enforcement of these policies, rather than the direct effect of implementation of the policy itself	Low
Excess alcohol	(A1) Increase excise taxes on alcoholic beverages	Price elasticity values consistent across HIC and LMICs (in spite of less relative affordability in LMICs)	High
	(A2) Establish minimum prices for alcohol where applicable	Consistent empirical evidence from two countries, confirming extensive previous supporting evidence	High
	(A3) Enact and enforce bans or comprehensive restrictions on exposure to alcohol advertising (across multiple types of media) - including in connection with sponsorships and activities targeting young people	Some empirical evidence demonstrating reductions in consumption resulting from restrictions, supported by extensive empirical and experimental evidence that increased marketing (including web-based marketing) increases attitudes and consumption. Evidence base is still maturing with respect to the specifics of the regulatory approach, and accurately estimating the potential impact, in the context of the changing global, web-based alcohol advertising landscape	Moderate
	(A4) Enact and enforce restrictions on the physical availability of retailed alcohol (via reduced hours of sale)	Limited empirical evidence presented by WHO, with no supporting Cochrane review. Supported by theoretical evidence, and empirical evidence from tobacco reduction, that availability is an important factor	Moderate
	(A5) Provide consumer information about, and label, alcoholic beverages to indicate, the harm related to alcohol	The totality of evidence points to a null, or very small effect, on drinking behaviour. It is possible that newer packaging designs could be more effective than previous designs, but more evidence is needed to confirm this	Low
	(A6) Enact and enforce an appropriate minimum age for purchase or consumption of alcoholic beverages and reduce density of retail outlets	There is limited empirical evidence for this recommendation. However, it is logical that an effectively enforced minimum legal drinking age does reduce the availability of alcohol to younger people	Low
Obesity	(O1) Use effective taxation measures to reduce consumption of unhealthy products such as sugar-sweetened beverages	Consistent evidence across several reviews	High
	(O2) Implement policies to protect children from the harmful impact of food marketing	Clear that comprehensive marketing bans could reduce consumption of unhealthy foods, but unclear applicability of current evidence base to broad population context. Supporting evidence from a systematic review of 44 natural experiment studies of restricting advertising to children (across a range of mediums), but the authors consider the overall quality of the evidence to be low. Comprehensive evidence base demonstrating the effectiveness of marketing to change children's preferences, but more real-world evidence is required to accurately ascertain the empirical effect of comprehensive marketing restrictions	Moderate
	(O3) Introduce menu labelling in food service to promote healthy diets (e.g., reduce total energy intake (kcal) and/or intake of sugars)	A Cochrane review summarizes the emerging evidence base, with meta-analysis of empirical studies supported by similar findings from experimental evidence	Moderate
	(O4) Limiting portion and package size to reduce energy intake and the risk of overweight/obesity	Evidence from a meta-analysis of RCT evidence - including real-world studies	High
	(O5) Public food procurement and service policies for healthy diets (e.g., to reduce the intake of free sugars, and to increase the consumption of legumes, wholegrains, fruits and vegetables)	Evidence from several reviews supports school-based policies for ensuring that food provided/available is healthy. This is supported by evidence from other reviews demonstrating successful programmes in other settings. Heterogeneity in intervention design and variable study quality is a feature of this evidence base, with few studies including appropriate control groups, and studies generally not capturing any effects of the policies on food consumed outside of the setting. However, there is a strong argument for these interventions to be included, alongside other diet-based policies, to improve the overall food environment	Moderate
	(O6) Reformulation policies for healthier food and beverage products (e.g., reduction of free sugars)	Evidence from several reviews demonstrating reformulation policies can be effective. Range of effect sizes reflects heterogeneity in reformulation policies adopted for various populations and food products. Consistent evidence that mandatory policies more effective than those reliant on voluntary action or industry self-regulation	High
	(O7) Subsidies on healthy foods and beverages (e.g., fruits and vegetables) as part of comprehensive fiscal policies for healthy diets	Consistent evidence across several reviews show effectiveness for increasing healthy food consumption, but outcomes such as total caloric intake not reported	Low
	(O8) Front-of-pack labelling as part of comprehensive nutrition labelling policies for facilitating consumers' understanding and choice of food for healthy diets	Empirical evidence base for effectiveness is fairly weak with the majority of evidence coming from modelling studies or evidence using self-report and examining consumer understanding and attitudes in response to FOPL. However, emerging evidence base suggesting that FOPL can also contribute to reformulation drives. Based on totality of evidence, likely that FOPL will produce small effects, if any, but could be a contributory policy to support other changes to the food environment and marketing	Low
Physical Inactivity	(P1) Implement urban and transport planning and design, at all levels of government, to provide compact neighbourhoods providing mixed-land use and connected networks for walking and cycling and equitable access to safe, quality public open spaces that enable and promote physical activity and active mobility (including wheelchairs, scooters and skates) by people of all ages and abilities	Clear empirical and supporting evidence that changes to urban design can increase physical activity rates through increased walking and cycling. However, no meta-analysis reported, and several studies reported null results, reflecting a maturing evidence base. Uncertainty regarding the potential magnitude of effect, potential waning/increasing over time, and overall effect on physical inactivity prevalence and obesity	Moderate
	(P2) Implement whole-of-school programmes that include quality physical education, and adequate facilities, equipment and programs supporting active travel to/from school and support physical activity for all children of all abilities during and after school	The totality of evidence suggests the potential for a small effect on total physical activity. Much of the evidence-base consists of multi-component interventions which generally include approaches like health education and curricular changes to increase the amount of physical activity conducted at school, but do not generally tackle broader structural issues such as the built environment outside of school and gender norms. However, it is logical that policies to integrate active school travel and exercise during the school day could be complimentary to a broader strategy, and there is evidence that some interventions can contribute to increased physical activity and be acceptable to large proportions of participants	Low
	(P3) Implement multi-component workplace physical activity programmes	Limited empirical evidence for population-level interventions. As for school-based interventions, it is logical that workplaces could be important partners in broader approaches to make physical activity easier, such as providing shower facilities for employees. Other possible interventions, such as flexible working hours to allow for active commuting in daylight, were not identified in any cited documents	Low
Hypertension	(H1) Public food procurement and service policies to reduce the intake of sodium	Evidence from several reviews supports school-based policies for ensuring that food provided/available is healthy. This is supported by evidence from other reviews demonstrating successful programmes in other settings. Heterogeneity in intervention design and variable study quality is a feature of this evidence base, with few studies including appropriate control groups, and studies generally not capturing any effects of the policies on food consumed outside of the setting. However, there is a strong argument for these interventions to be included, alongside other diet-based policies, to improve the overall food environment	Moderate
	(H2) Reformulation policies for reduction of sodium content	Evidence from several reviews demonstrating reformulation policies can be effective. Range of effect sizes reflects heterogeneity in reformulation policies adopted for various populations and food products. Consistent evidence that mandatory policies more effective than those reliant on voluntary action or industry self-regulation	High
Depression	(D1) Interventions to improve the social determinants of health, including social security policies, housing improvements, and community wealth building	Some evidence that these interventions can reduce depression, but insufficient consistency to be confident in the factors which drive a successful, rather than null, outcome. One study demonstrated a reduction in anti-depressant prescribing (objectively, using electronic health records) in a deprived area of the UK, following introduction of a community wealth building (CWB) programme which involved working with local anchor institutions to address upstream economic inequalities through measures like equitable distribution of investments and procurement, and commitment to paying a ‘living wage’ to all employees, which may demonstrate an evolving evidence base	Low
Traumatic Brain Injury	(Tr1) Mandate the use of motorcycle helmets (all aged passengers)	Clear evidence from a systematic review reported by WHO, reporting studies from the US showing reductions in head injuries and hospitalisation amongst children after policy introduction. Further supportive evidence from WHO and Cochrane, including from LMICs	Moderate
	(Tr2) Mandate the use of bicycle helmets (children)	Clear evidence from a Cochrane review reporting studies from the US and Canada showing reductions in head injuries and hospitalisation after policy introduction. Further supportive evidence from WHO	Moderate
	(Tr3) Provision of free bicycle helmets to children aged under 12	Included as a component in several multi-component interventions reported by WHO and Cochrane	Low
Low Educational Attainment	(E1) Provide financial support (including removing school fees, conditional payments to schools, conditional cash transfers to households) for children to attend school, where financial barriers would otherwise exist	Campbell collaboration review reported direct evidence from 7 studies (free primary schools in Uganda n = 3 studies, studies of primary school tuition waivers (e.g., via conditional payments to the school) n = 3 from Haiti, Pakistan, Ecuador, and secondary school tuition waivers n = 1 from Ghana, supported by 10 further studies which were multi-component but included free schooling)	Moderate
	(E2) Provide free lunches in primary schools, where a lack of adequate food would otherwise be a barrier to school attendance	Campbell collaboration review reported two studies reported effects on boys and girls combined. One examined the effects of policies to improve access and quality of schooling in Burkina Faso, using secondary data from surveys and RCTs, reporting significant benefits, mainly for in-school feeding programmes and also out of school rations in some cases. Another considered the effect of a national policy to provide free lunches to all primary school children in India and reported significant benefits to primary school enrolment. Supportive evidence from ten studies reported effects on girls specifically	Moderate
	(E3) Raise the mandatory school leavers age in HICs	Report for the European Commission on Economic Policy reported that expanding compulsory education ages statistically significantly increased educational attainment, and had equity benefits. Supported by several references to other reviews of empirical data which each find increases in attainment associated with later compulsory leaving age, and equity effects	High
	(E4) Provide school materials, where there would otherwise be difficulty in affording and obtaining these	Campbell collaboration review reported similar results to those for adequate food, with 2 studies reporting benefits when effects are combined for boys and girls, and a more mixed picture for girls alone. However, the evidence quality was more mixed, the interventions were more heterogeneous, and the majority of evidence was from multi-component interventions (e.g., combined uniform provision with support for tuition fees)	Low
	(E5) Improve geographical access to schools	Campbell collaboration review reported that interventions to address inadequate access to school were consistently effective, but the authors were unable to disentangle the specific effects of these interventions from the multi-component programmes they were part of	Low
	(E6) Improve water supply and sanitation in schools	Campbell collaboration review reported that interventions to address water and sanitation in schools were consistently effective, but the authors were unable to disentangle the specific effects of these interventions from the multi-component programmes they were part of	Low
Air Pollution	(Ai1) Replacement and maintenance programmes providing cleaner cooking stoves for those currently using biomass fuels on traditional stoves or open fire	Clear evidence from several RCTs, but no systematic review, that this can reduce kitchen PM2.5 and CO. Limited data from the use of individual air pollution monitors. Supported by a systematic review of qualitative evidence to understand predictors of success	Moderate
	(Ai2) Urban interventions to reduce density of traffic, including: low emission zones, even-odd restrictions on cars	Clear evidence from studies reported by Cochrane review that these restrictions reduce PM2.5, PM10, NO and CO, in both summer and winter conditions	Moderate
	(Ai3) Postponement of non-essential polluting activities on high-pollution days	Clear evidence from studies reported by Cochrane review that these restrictions reduce PM10 and cardiovascular hospitalisations	Moderate
	(Ai4) Comprehensive (marketing, sale and distribution) coal bans for residential heating	Cochrane review presents evidence from one country (Ireland) that this can reduce respiratory and cardiovascular hospitalisations and mortality, but no empirical evidence of the associated reduction in pollution	Low
Hearing Impairment	(He1) Worksites exceeding recognised noise thresholds should reduce noise through improving equipment where feasible, and should provide and mandate the use of adequate hearing protection, with regular monitoring	Clear evidence from a Cochrane review that occupational policies to monitor and reduce noise exposure, and to provide, and mandate the use of, adequate hearing protection, can reduce acquisition of short- and longer-term hearing impairment	Moderate

HICs = High income countries. LMICs = Low- and middle-income countries. WHO = World Health Organization. RCT = Randomised Controlled Trials. FOPL = Front of pack labelling. PM = Particulate matter. CO = Carbon monoxide. NO = Nitrous Oxide.

Across the review, and through professional networks, we identified nine existing population-level intervention frameworks, for NCDs in general (n = 3), diet and/or obesity (n = 4), and alcohol (n = 2) (Supplementary Table 4). Comparing the intervention types described by these frameworks, with the high- and moderate-confidence recommendations from our review, we considered four intervention types for our framework: fiscal interventions (changing the affordability of healthy and unhealthy products/behaviours), marketing and/or advertising interventions (changing the way the population are exposed to healthy and unhealthy products), availability interventions (changing the population's access to healthy and unhealthy products/behaviours), and legislative interventions (changing the law to mandate certain behaviours from industry or the population).

Table 2 shows the derived population-level dementia risk reduction intervention framework, populated by interventions with high- and moderate-confidence. We identified most interventions of the availability type (n = 8) and legislative type (n = 8), followed by fiscal (n = 5) and marketing/advertising (n = 5).

**Table 2:** Population-level dementia risk reduction framework.

Risk factor	Fiscal interventions	Marketing/advertising interventions	Availability interventions	Legislative interventions
Tobacco Smoking	(T1) Increase excise taxes and prices on tobacco products.^a^	(T2) Implement plain/standardized packaging and/or large graphic health warnings on all tobacco packages.^b^ (T3) Enact and enforce comprehensive bans on tobacco advertising, promotion and sponsorship.^b^	(T4) Eliminate exposure to second-hand tobacco smoke in all indoor workplaces, public places, public transport.^a^	
Excess Alcohol	(A1) Increase excise taxes on alcoholic beverages.^a^ (A2) Establish minimum prices for alcohol.^b^	(A3) Enact and enforce bans or comprehensive restrictions on exposure to alcohol advertising (across multiple types of media) - including in connection with sponsorships and activities targeting young people.^a^	(A4) Enact and enforce restrictions on the physical availability of retailed alcohol (via reduced hours of sale).^a^	
Obesity	(O1) Use effective taxation measures to reduce consumption of unhealthy products such as sugar-sweetened beverages.^a^	(O2) Implement policies to protect children from the harmful impact of food marketing.^a^ (O3) Introduce menu labelling in food service to promote healthy diets (e.g., reduce total energy intake (kcal) and/or intake of sugars).^a^	(O4) Limiting portion and package size to reduce energy intake and the risk of overweight/obesity.^b^ (O5) Public food procurement and service policies to promote healthy diets (e.g., to reduce the intake of free sugars; and to increase the consumption of legumes, wholegrains, fruits and vegetables).^b^	(O6) Reformulation policies for healthier food and beverage products (e.g., reduction of free sugars).^b^
Physical Inactivity			(P1) Implement urban and transport planning and design, at all levels of government, to provide compact neighbourhoods providing mixed-land use and connected networks for walking and cycling and equitable access to safe, quality public open spaces that enable and promote physical activity and active mobility (including wheelchairs, scooters and skates) by people of all ages and abilities.^a^	
Hypertension			(H1) Public food procurement and service policies to reduce the intake of sodium.^a^	(H2) Reformulation policies for reduction of sodium content.^a^
TBI				(Tr1) Mandate the use of motorcycle helmets (all aged passengers).^b^ (Tr2) Mandate the use of bicycle helmets (children).^b^
Low Educational Attainment	(E1) Provide financial support (including removing school fees, conditional payments to schools, conditional cash transfers to households) for children to attend school, where financial barriers would otherwise exist.^c^		(E2) Provide free lunches in primary schools, where a lack of adequate food would otherwise be a barrier to school attendance.^c^	(E3) Raise the mandatory school leavers age in HICs.^b^
Air Pollution			(Ai1) Replacement and maintenance programmes providing cleaner cooking stoves for those currently using biomass fuels on traditional stoves or open fires.^c^	(Ai2) Urban interventions to reduce density of traffic, including: low emission zones, even-odd restrictions on cars.^a^ (Ai3) Postponement of non-essential polluting activities on high-pollution days.^a^
Hearing Impairment				(He1) Worksites exceeding recognised noise thresholds should reduce noise through improving equipment where feasible, and should provide and mandate the use of adequate hearing protection, with regular monitoring.^b^

TBI = Traumatic brain injury. HICs = High income countries

a Supporting evidence for this recommendation includes data from both high and low/middle income countries.

b Supporting evidence for this recommendation includes data from high income countries only.

c Supporting evidence for this recommendation includes data from low/middle income countries only.

For 13 of the recommendations we found supporting evidence from both HIC and LMICs, for 10 we found evidence from HICs only, and for three we found evidence from LMICs only (Table 2).

We did not identify any high- or moderate-confidence recommendations for diabetes, depression or social isolation.

Table 3 reports key information for policymakers regarding each recommendation, including example effect sizes, evidence sources, contextual considerations, and implementation guidance.

**Table 3:** Supporting information for the framework interventions.

Intervention	Example outcome of intervention on risk factor	Key source(s) of evidence of effectiveness and effect estimate(s)	Key considerations	Further planning and implementation guidance
(T1) Increase excise taxes and prices on tobacco products	10% increase in price results in 4–5% relative reduction in consumption	*Ortegón et al., 2012. Cost effectiveness of strategies to combat cardiovascular disease, diabetes, and tobacco use in sub-Saharan Africa and South East Asia: mathematical modelling study. BMJ* *WHO, 2021. WHO Technical manual on tobacco tax administration*	Recognition that there are different types of taxation. Comprehensive assessment of this question available in WHO manual on tobacco tax policy (most recently updated 2021) which recognises that different countries have different taxation systems and should make appropriate decisions for their local context. However, excise taxes (specific taxes on tobacco products), which are regularly updated to keep up with inflation and progressively make cigarettes less affordable, are recommended as the most effective measure. Must be implemented alongside other work to ensure reductions in illegal tobacco trade which bypasses excise taxation. Tobacco has become more affordable in LMICs in recent years, debunking concerns raised by industry that taxes will lower overall tax revenue, or drive inequalities	*WHO, 2021. WHO technical manual on tobacco tax policy and administration*
(T2) Implement plain/standardized packaging and/or large graphic health warnings on all tobacco packages	5% relative reduction in smoking prevalence Assumed baseline prevalence of 25%, there is a 5% (credible range 2%–8%) relative reduction in prevalence, using a 5-year time horizon. Using a 40-year time horizon, the estimate is 10% relative prevalence reduction (credible range 5%–15%)	*Levy et al. 2017. The Impact of Implementing Tobacco Control Policies: The 2017 Tobacco Control Policy Scorecard. J Public Health Manag Pract*. Synthesises empirical evidence from HICs on tobacco policy from US taskforce reports, reviews, and primary articles *McNeill et al., 2017. Tobacco packaging design for reducing tobacco use. Cochrane Review*	Unless updated on a regular basis with new content, the effectiveness of graphic warning labels may wane over time as consumers become too accustomed to their appearance. Messages can be reinforced by mass media campaigns. Evidence stronger for graphic health warnings than for plain packaging	*WHO, 2016. Plain packaging of tobacco products: evidence, design and implementation*
(T3) Enact and enforce comprehensive bans on tobacco advertising, promotion and sponsorship	4% relative reduction in smoking prevalence Assumed baseline prevalence of 25%, there is a 4% (credible range 2%–6%) relative reduction in prevalence, using a 5-year time horizon. Using a 40-year time horizon, the estimate is 6% relative prevalence reduction (credible range 3%–9%)	*Levy et al., 2017. The Impact of Implementing Tobacco Control Policies: The 2017 Tobacco Control Policy Scorecard. J Public Health Manag Pract* *Lovato et al., 2011. Impact of tobacco advertising and promotion on increasing adolescent smoking behaviours. Cochrane Review*	Include bans on direct advertising, such as TV, radio, magazine, newspaper, billboard, and retail point-of-sale advertising, and bans on indirect marketing, such as free distribution of products, promotional discounts, the appearance of tobacco products in TV or films, sponsorship of sports and music occasions, and the distribution of nontobacco products identified with tobacco brand names. Online advertising and indirect marketing efforts not included by the interventions which have been evaluated, and may offset these effects unless these channels are also targeted. Evidence that younger people and those with higher education benefit more	*WHO, 2003. Framework Convention on Tobacco Control*
(T4) Eliminate exposure to second-hand tobacco smoke in all indoor workplaces, public places, public transport	10% relative reduction in smoking prevalence Assumed baseline prevalence of 25%, there is a 10% (credible range 5%–15%) relative reduction in prevalence, using a 5-year time horizon. Using a 40-year time horizon, the estimate is 12.5% relative prevalence reduction (credible range 7%–19%)	*Levy et al., 2017. The Impact of Implementing Tobacco Control Policies: The 2017 Tobacco Control Policy Scorecard. J Public Health Manag Pract* *Frazer et al., 2016. Legislative smoking bans for reducing harms from secondhand smoke exposure, smoking prevalence and tobacco consumption. Cochrane Review* *Frazer et al., 2016. Impact of institutional smoking bans on reducing harms and secondhand smoke exposure. Cochrane Review*	May have smaller effects if smoke-free policies are already prominent in private worksites; or if there is low compliance due to weak enforcement or a lack of antitobacco social norms	*WHO, 2003. Framework Convention on Tobacco Control*
(A1) Increase excise taxes on alcoholic beverages	10% increase in price results in 5.1%–7.7% relative reduction in consumption	*Chisholm et al., 2018. Are the “Best Buys” for alcohol control still valid? An update on the comparative cost-effectiveness of alcohol control strategies at the global level. J Stud Alcohol Drugs* *WHO, 2017. Resource tool on alcohol taxation and pricing policies*	Levying taxes should be combined with other price measures, such as bans on discounts or promotions; and work to reduce illegal alcohol consumption which bypasses taxation. Detailed discussion of mechanism of excise taxation - i.e., taxation rate based on strength, quantity, or cost - in the 2017 resource, with conclusion that each has their strengths and weaknesses but the overall effect will consistently be reduced alcohol consumption (but differences in the effect on overall tax revenue and rate of substitutions)	*WHO, 2019. SAFER - A world free from alcohol related harms. The Technical Package* *WHO, 2017. Resource tool on alcohol taxation and pricing policies*
(A2) Establish minimum prices for alcohol	10% increase in minimum price results in a 3.4% (95% CI 1.4%–8.0%) relative reduction in total alcohol sales	*Robinson et al., 2021. Evaluating the impact of minimum unit pricing (MUP) on off-trade alcohol sales in Scotland: an interrupted time–series study. Addiction.* Interrupted time series analysis estimating the effect of the 2018 MUP in Scotland on off-trade alcohol sales, compared to England (no MUP). Supported by a similar analysis from a different set of academics *(O'Donnell, BMJ, 2019)* which reached similar conclusions using consumer panel data. A further paper *(Wyper, Lancet, 2023)* found an associated reduction in alcohol-attributable hospital mortality *Stockwell et al., 2012. Does minimum pricing reduce alcohol consumption? The experience of a Canadian province. Addiction*	SAFER package frames MUP as an adjunct to excise taxation, which may have the effect of causing retailers to lower the cost of their products and absorb the increased taxation (or in the case of ad valorem taxes, avoid them)	*WHO, 2019. SAFER - A world free from alcohol related harms. The Technical Package* *WHO, 2017. Resource tool on alcohol taxation and pricing policies*
(A3) Enact and enforce bans or comprehensive restrictions on exposure to alcohol advertising (across multiple types of media) - including in connection with sponsorships and activities targeting young people	1.2% absolute reduction in prevalence of hazardous/harmful drinking (Source study find a 3.0% (95% CI 1.0%–6.0%) reduction in drinking volume per additional unit (none, voluntary or self-regulation, partial statutory restriction, ban) of marketing restriction for beer, wine and spirits combined across 4 types of media (national TV, national radio, print media, and billboards). WHO team have then simulated this effect size to all world regions, accounting for their existing level of policy in place)	*Cook et al., 2014. Are alcohol policies associated with alcohol consumption in Low and Middle income countries? Addiction* *Rossow et al., 2021. The alcohol advertising ban in Norway: effects on recorded alcohol sales. Drug Alcohol Rev* *Brown et al., 2016. Association Between Alcohol Sports Sponsorship and Consumption: A Systematic Review, Alcohol and Alcoholism*	Effect estimate doesn't include bans on web advertising, sponsorship; or the differentiation between domestic and cross-border marketing. SAFER package notes that: restricting only one aspect of the marketing mix often results in an expansion of activity in other parts of the mix. In general, the more complete the regulation on marketing activities, the easier it will be to implement the regulation and the more effective it will be in reducing alcohol-related harm. That is why a comprehensive ban or set of restrictions is preferred. Such frameworks should ideally incorporate all forms of new and emerging media as well as existing media and other promotional channels There is a need for supra-national co-operation to effectively tackle the global advertising landscape, with legislative implications. There is comprehensive evidence that alcohol industry self-regulation is not an effective tool for reducing alcohol-related harm, and these endeavours should be led by governments. Newer options need to be evaluated, such as removing tax exemptions for alcohol advertising spending, and blocking any collection of consumer data for the purposes of alcohol marketing	*WHO, 2019. SAFER - A world free from alcohol related harms. The Technical Package* *WHO, 2022. Reducing the harm from alcohol by regulating cross-border alcohol marketing, advertising and promotion. A technical report*
(A4) Enact and enforce restrictions on the physical availability of retailed alcohol (via reduced hours of sale)	1.8–2.1% (male), 4% (female) relative reduction in prevalence of hazardous/harmful drinking (Source study reports a regression coefficient for restricted hours of sale of −0.88 (95% CI −1.44, 0.32). WHO team have them simulated this effect size to all world regions, assuming a gamma distribution, to assess for the reduction in hazardous and harmful drinking that would be achieved)	*Cook et al., 2014. Are alcohol policies associated with alcohol consumption in Low and Middle income countries? Addiction*	Alongside restricting hours of sale, other mechanisms described in the SAFER package include: regulate the number, density and location of retail alcohol outlets; establish a national legal minimum age for purchase and consumption of alcohol; and restrict the use of alcohol in public places	*WHO, 2019. SAFER - A world free from alcohol related harms. The Technical Package*
(O1) Use effective taxation measures to reduce consumption of unhealthy products such as sugar-sweetened beverages	10% increase in price results in 8%–15.9% relative reduction in consumption of sugar-sweetened beverages	Sugar-Sweetened Beverages: *World Bank, 2020. ‘Taxes on Sugar-Sweetened Beverages: International Evidence and Experiences*' *Andreyeva et al., 2022. Outcomes Following Taxation of Sugar-Sweetened Beverages: A Systematic Review and Meta-analysis. JAMA Network Open* *WHO, 2022. Policy brief on fiscal policies to promote healthy diets* *WHO, 2022. WHO manual on sugar-sweetened beverage taxation policies to promote healthy diets*	Clear evidence of a healthy equity effects of the taxation, with the greatest reductions in purchasing found in lower SES groups WHO modelling included: carbonated soft drinks, non-carbonated soft drinks, fruit and vegetable juices, liquid powder concentrates, flavoured water, energy and sports drinks, ready-to-drink tea and coffee, flavoured milk drinks; and excluded: low calorie/low sugar product categories as these contained a variable mix of products with or without added sugars, and/or artificial sweeteners. WHO assumed no substitution to untaxed beverages (based on systematic review by Andreyeva, 2022). For research purposes, SSB consumption volume is converted into the number of daily servings per capita, assuming a serving size of 355 ml.	*WHO, 2022. WHO manual on sugar-sweetened beverage taxation policies to promote healthy diets* *WHO, 2022. Policy brief on fiscal policies to promote healthy diets* *WHO, 2022. SSB taxes in the WHO European region: success through lessons learned and challenges faced*
(O2) Implement policies to protect children from the harmful impact of food marketing	60 kcal (95% CI 3.1–116.9) absolute reduction/day/person	*Russell et al., 2019. The effect of screen advertising on children's dietary intake: A systematic review and meta-analysis. Obes Rev* *WHO, 2022. Protecting children from the harmful impact of food marketing: policy brief*	The evidence for policies to reduce the power of, and children's exposure to, food and non-alcoholic beverage marketing is still emerging; however, there is clear evidence that such marketing practices are abundant for unhealthy food high in fats, sugars and salt; and evidence on the impact of marketing on children is unequivocal and has recently been updated in a new systematic review The 2022 policy brief states the importance of comprehensive (all forms of media), and mandated marketing restrictions, over partial and voluntary approaches which were historically employed, and are less likely to be effective (and industry are likely to lobby for). As for alcohol marketing, important to recognise the cross-national nature of digital media. Also importance of protecting children up to the age of 18, rather than just under 12s	*WHO, 2016. Tackling food marketing to children in a digital world: trans-disciplinary perspectives* *WHO, 2020. Regional action framework on protecting children from the harmful impact of food marketing in WPRO* *WHO, UNICEF, 2018. A Child Rights-Based Approach to Food Marketing: A Guide for Policy Makers*
(O3) Menu labelling in food service to promote healthy diets (e.g., reduce total energy intake (kcal) and/or intake of sugars)	7.8% relative reduction in calories consumed per sitting Meta-analysis of the restaurant-based RCTs found a reduction in energy purchased of 47 kcal (95% CI 15–78) equating to a relative reduction of 7.8% (95% CI 2.5%–13.1%). Evidence from other high-quality real-world studies, and the lab-based experiments, supported this finding	*Crockett et al., 2018. Nutritional labelling for healthier food or non-alcoholic drink purchasing and consumption. Cochrane Review* *Updated Cochrane review in progress*	Most studies evaluated labelling on menus or menu boards, where a range of products were available. Other studies evaluated settings which provided only one food/drink option, but included a nutritional label on the packaging. Some assessed absolute energy labels, with no other information/formatting, others assessed traffic light formats, or energy labels with further information on nutritional content or exercise equivalents	Guidance from WHO in print
(O4) Limiting portion and package size to reduce energy intake and the risk of overweight/obesity	8.5%–13.5% relative reduction in calories Meta-analysis of 58 studies found a small to moderate effect of portion, package, or tableware size on consumption (SMD 0.38, 95% CI 0.29–0.46), with effects consistent for adults and children. Which would equate to an 8.5%–13.5% (144–228 kcal) reduction in calories, if achieved across the whole diet; and an absolute reduction of 144–228 kcal per day in the UK population	*Hollands et al., 2015. Portion, package or tableware size for changing selection and consumption of food, alcohol and tobacco. Cochrane Review* *Updated Cochrane review in progress*	Likely to have greatest effect by reducing size at the larger end of the scale, than changes to already smaller portions/packages/tableware	
(O5) Public food procurement and service policies to promote healthy diets (e.g., to reduce the intake of free sugars; and to increase the consumption of legumes, wholegrains, fruits and vegetables)	0.18 (95% CI 0.05–0.31) reduction in servings per day of SSB (serving = 355 mls), 0.76 (95% CI 0.37–1.16) additional servings per day of fruit (serving = 80 g)	*Micha et al., 2018. Effectiveness of school food environment policies on children's dietary behaviours: A systematic review and meta-analysis. PloS One* *Driessen et al., 2014. Effect of changing the school food environment. Obes Rev* *Niebylski et al., 2014. Healthy Food Procurement Policies and Their Impact. Int J Environ Res Public Health*	Evidence strongest for schools, but empirical evidence also shows effectiveness in other settings such as workplaces, hospitals, and nursing homes. These interventions do not generally impact consumption outside of the setting, and should therefore be considered as part of broader food environment policy 2021 action framework suggests that public food procurement could influence private sector employers to follow suit. It also points to evidence from Brazil where such policies have had co-benefits of guaranteed income for local farmers, and reducing climate footprint	*WHO, 2021. Action framework for developing and implementing public food procurement and service policies for a healthy diet*
(O6) Reformulation policies for healthier food and beverage products (e.g., reduction of free sugars)	2–11% relative reduction in dietary sugar per person per day (resulting in average 1.04 kg absolute reduction in body weight)	*Hashem et al., 2019. Effects of product reformulation on sugar intake and health-a systematic review and meta-analysis. Nutr Rev*	Reformulation may result from policies setting mandatory limits or voluntary targets for nutrient content in food and beverage products, or it may happen in the absence of a specific reformulation policy, as result of industry response to e.g., a FOPL or food or beverage tax policy. Evidence from the contributing systematic reviews suggest that mandatory limits have been more effective than voluntary/industry-led ones	
(P1) Implement urban and transport planning and design, at all levels of government, to provide compact neighbourhoods providing mixed-land use and connected networks for walking and cycling and equitable access to safe, quality public open spaces that enable and promote physical activity and active mobility (including wheelchairs, scooters and skates) by people of all ages and abilities	20% relative increase in cycling journeys	The WHO Europe 2006 report ‘*Physical activity and health in Europe: evidence for action*’ presents two case studies: the 2003 introduction of a congestion charge in London, England, and the 1999–2002 major investment in cycling infrastructure (alongside mass media campaign) in Odense, Denmark. Both are reported to have been associated with a 20% increase in cycling journeys. No references to peer-reviewed literature with detailed methodology are available	Clear overlap with this recommendation, and policies to reduce air pollution	*WHO, 2018. ACTIVE: a technical package for increasing physical activity* *WHO Europe, 2018. Towards More Physical Activity in Cities* *WHO, 2022. Compendium of WHO and other UN guidance on health and environment*
(H1) Public food procurement and service policies to reduce the intake of sodium	0.17 g (95% CI 0.10–0.24) absolute reduction/person/day in dietary sodium	*Micha et al., 2018*. *Effectiveness of school food environment policies on children's dietary behaviors: A systematic review and meta-analysis. PloS One* *Driessen et al., 2014. Effect of changing school food environment. Obes Rev* *Niebylski et al., 2014. Healthy Food Procurement Policies and Their Impact. Int J Environ Res Public Health* *McLaren et al., 2016. Population-level interventions in government jurisdictions for dietary sodium reduction. Cochrane Review*	2021 action framework suggests that public food procurement could influence private sector employers to follow suit. It also points to evidence from Brazil where such policies have had co-benefits of guaranteed income for local farmers, and reducing climate footprint	*Action framework for developing and implementing public food procurement and service policies for a healthy diet* *WHO, 2021. Global sodium benchmarks for different food categories* *WHO, 2016. SHAKE the salt habit: technical package for salt reduction*
(H2) Reformulation policies for reduction of sodium content	5–10% (−0.57 g) reduction/person/day in dietary sodium, (resulting in approximate −0.53 mmHg absolute reduction of SBP)	*Gressier et al., 2021. What is the impact of food reformulation on individuals' behaviour, nutrient intakes and health status? A systematic review of empirical evidence. Obes Rev* *McLaren et al., 2016. Population-level interventions in government jurisdictions for dietary sodium reduction. Cochrane review*	Reformulation may result from policies setting mandatory limits or voluntary targets for nutrient content in food and beverage products, or it may happen in the absence of a specific reformulation policy, as result of industry response to e.g., a FOPL or tax policy. Evidence from the contributing systematic reviews suggest that mandatory limits have been more effective	*WHO, 2021. Global sodium benchmarks for different food categories* *WHO, 2016. SHAKE the salt habit: technical package for salt reduction*
(Tr1) Mandate the use of motorcycle helmets (all aged passengers)	54% absolute reduction in head injuries requiring hospitalisation Median reduction of −54% (95% CI –49% to −59%) in head injuries	*WHO, 2023. Helmets: a road safety manual for decision-makers and practitioners, 2nd edition.* Includes a systematic review of legislation for mandatory motorcycle helmet use *(Peng, 2017, Am J Prev Med)* reports 5 studies from the USA. The WHO report also cites several single studies from a mix of other countries including LMICs demonstrating effectiveness of legislation	Importance of appropriate accompanying enforcement, education/promotion, and availability of helmets is stressed, in order for legislation to be effective. Evidence suggests full face helmets, and firm fitting helmets, are relatively more protective than alternatives	*WHO, 2023. Helmets: a road safety manual for decision-makers and practitioners, 2*nd *edition*
(Tr2) Mandate the use of bicycle helmets (children)	18% (95% CI 11.5–24.3) absolute reduction in head injuries requiring hospitalisation	*MacPherson, 2008. Bicycle helmet legislation for the uptake of helmet use and prevention of head injuries. Cochrane review*	Importance of appropriate accompanying enforcement, education/promotion, and availability of helmets is stressed, in order for legislation to be effective. Provision of free bicycle helmets to children aged under 12, has been estimated to increase odds of wearing a helmet by 4.35 times, which in turn could reduce the risk of head injury by 63–88% in the event of an accident (*Owen, 2011. Non-legislative interventions for the promotion of cycle helmet wearing by children. Cochrane review*)	*WHO, 2023. Helmets: a road safety manual for decision-makers and practitioners, 2nd edition*
(E1) Provide financial support (including removing school fees, conditional payments to schools, conditional cash transfers to households) for children to attend school, where financial barriers would otherwise exist	2%–75% absolute increase in primary school enrolment (immediate effect of policy) - heavily dependent on baseline enrolment rate Additional 0.72 years of extra schooling 30% relative increase (from 9% to 11.8%) in secondary school completion rate 11.4% absolute increase in rate of higher education enrolment	*Grogan et al., 2009. Universal primary education and school entry in Uganda. Journal of African Economies* *Keats et al., 2018. Women's schooling, fertility, and child health outcomes: Evidence from Uganda's free primary education program. Journal of Development Economics* *Psaki, S. et al., 2022. Policies and interventions to remove gender-related barriers to girls' school participation and learning in low- and middle-income countries: A systematic review of the evidence. Campbell Systematic Reviews* *Patel-Campillo et al., 2022. Breaking the poverty cycle? Condit'onal cash transfers and higher education attainment. International Journal of Educational Development*	School infrastructure must be adequately prepared to maintain educational standards, given the potentially large increases in numbers of students Evidence of universal effects, and closing the gap between socioeconomic groups	
(E2) Provide free lunches in primary schools, where a lack of adequate food would otherwise be a barrier to school attendance	18.5%–26% absolute increase in primary school enrolment 0.41 additional grades completed (either by extra years of education, or by reducing grade repetition rate)	*Kaur et al., 2017. Essays in human capital development* *Kazianga et al., 2013. The effects of “girl-friendly” schools: Evidence from the BRIGHT school construction program in Burkina Faso. American Economic Journal: Applied Economics* *Kazianga et al., 2016. The Medium-Term Impacts of Girl-Friendly Schools: 7-Year Evidence from School Construction in Burkina Faso (No. 1609). Oklahoma State University, Department of Economics and Legal Studies in Business*	Evidence of universal effects, and closing the gap between genders and socioeconomic groups	
(E3) Raise the mandatory school leavers age in HICs	0.25–0.3 absolute increase in average years of education (national average) – consistent effect across three studies of 14 European countries, from reforms in late 20th century	*Aakvik et al., 2010. Measuring heterogeneity in the returns to education using an education reform. European Economic Review* *Brunello et al., 2009. Changes in Compulsory Schooling, Education and the Distribution of Wages in Europe. Economic Journal* *Meghir et al., 2005. Educational Reform, Ability, and Family Background. American Economic Review*	Reliant on sufficient provision of educational infrastructure and opportunities, and economic considerations of the effects of young people being later to join the workforce and paid employment. Evidence of universal effects, and closing the gap between socioeconomic groups	
(Ai1) Replacement and maintenance programmes providing cleaner cooking stoves for those currently using biomass fuels on traditional stoves or open fires	Absolute reduction in kitchen PM2.5 of −0.46 μg/m^3^ (95% CI 0.33–0.60), and CO of 5.7 μg/m^3^ (95% CI 3.9–7.5) from solid fuel stoves with chimneys.	*WHO, 2014. WHO guidelines for indoor air quality: household fuel combustion.* Development of these guidelines included an SR of clean fuel interventions for people currently using biomass or coal as the primary cooking fuel, with open fires or traditional stoves *Ye et al., 2022. Effects of a Liquefied Petroleum Gas Stove Intervention on Gestational Blood Pressure: Intention-to-Treat and Exposure-Response Findings From the HAPIN Trial. Hypertension*	A review of contextual factors finds that no factors guarantee success, but to be widely adopted, interventions must take relevant contextual factors into account e.g., can the traditional foods be cooked on the provided stove, is there long-term support for replacing parts, is the fuel supply reliable to that setting	*WHO, 2014. WHO guidelines for indoor air quality: household fuel combustion* *WHO, 2022. Compendium of WHO and other UN guidance on health and environment*
(Ai2) Urban interventions to reduce density of traffic, including: low emission zones, even-odd restrictions on cars	10.4% reduction in PM2.5, 6.8%–27% reduction in PM10, 2.2–3.5% reduction in NO2, and 9% reduction in CO	*Burns, 2019. Interventions to reduce ambient particulate matter air pollution and their effect on health. Cochrane review*		*PHE, 2020. Review of interventions to improve outdoor air quality and public health: principal interventions for local authorities* *WHO, 2022. Compendium of WHO and other UN guidance on health and environment*
(Ai3) Postponement of non-essential polluting activities on high-pollution days	16.9% reductions in PM10, achieved by multi-component restrictions on high-polluting days (driving restrictions, shutdown of certain major stationary emitters, street sweeping, traffic enforcement activities, and restriction on the use of biomass combustion for residential heating)	*Burns, 2019. Interventions to reduce ambient particulate matter air pollution and their effect on health. Cochrane review*		*PHE, 2020. Review of interventions to improve outdoor air quality and public health: principal interventions for local authorities* *WHO, 2022. Compendium of WHO and other UN guidance on health and environment*
(He1) Worksites exceeding recognised noise thresholds should reduce noise through improving equipment where feasible, and should provide and mandate the use of adequate hearing protection, with regular monitoring	60% reduction in occupationally-acquired hearing impairment Avoidance of hearing loss OR 0.4 (95% CI 0.23–0.69), at 5 years	*Tikka et al., 2017. Interventions to prevent occupational noise-induced hearing loss. Cochrane review*	Worksites exceeding recognised thresholds (80 dB(A)) should provide and mandate the use of adequate hearing protection, with regular audiometric monitoring of those working in areas which continue to exceed thresholds despite installation of quieter equipment	*WHO, 2018. Addressing the rising prevalence of hearing loss*

95% CI = 95% Confidence Interval. LMICs = low- and middle-income countries. SES = socioeconomic status. SSB = sugar-sweetened beverages. SMD = standardised mean difference. SBP = systolic blood pressure. PM = particular matter. CO = carbon monoxide. NO2 = nitrous oxide. OR = odds ratios.

N.B. “credible ranges” are quoted from the source literature by Levy et al., and are defined as “credible ranges for effect sizes based on the number of studies conducted, variation in results, and strength of evidence.”

### Findings by risk factor

#### Tobacco smoking

Consistent evidence, from across several reviews, demonstrates the effectiveness of increasing excise taxes, smoking bans in public places, and comprehensive marketing bans and packaging controls. In particular, taxation policies and smoking bans, are supported by evidence from both HIC and LMICs.

The WHO also recommends introduction of a minimum legal age for smoking, which aims to delay the age of smoking initiation. However, we only identified supporting empirical evidence from a 2005 Cochrane review (Supplementary Table 2A) focussed on interventions to improve enforcement of this legislation (not the direct empirical effect of the legislation itself), so we have graded this as a low-confidence recommendation.

#### Excess alcohol

Consistent evidence demonstrated the effectiveness of excise taxes and minimum unit prices for alcohol, and we recommend these with high-confidence. Empirical data for the newer policy measure, minimum unit pricing, was only reported from HICs.

There was also clear evidence for comprehensive marketing bans, and from LMICs for interventions to restrict the physical availability of alcohol, for example, through limiting the permitted hours of sale, or reducing the density of licensed alcohol vendors.

#### Obesity

Consistent evidence, from several reviews, demonstrates the effectiveness of interventions targeting diet to reduce obesity, specifically increasing taxation of unhealthy products such as sugar-sweetened beverages, reducing portion/package sizes, and reformulation policies to reduce the sugar content of available foods – supported by consistent evidence that mandatory policies are more effective than industry-led voluntary policies.

We found clear evidence for food procurement policies, e.g., improving the healthiness of food available in schools or hospitals, and for marketing policies to restrict advertising of unhealthy foods to children, and menu labelling in restaurants – though these evidence bases are still developing.

Evidence for front of food pack labelling is mixed, and equitable population-level effects are only likely to be achieved if they lead to reformulation of products – we grade this as a low-confidence recommendation. We also grade subsidies of healthy foods as a low-confidence recommendation because, although the evidence base was consistent and these intervention could clearly be an important part of a whole-system approach to obesity prevention, the only reported outcomes were improvements to healthy food intake, and not other important proxies for obesity such as total calorie intake.

#### Physical inactivity

The evidence base for physical inactivity is relatively less mature than for smoking, alcohol, or poor diet. We make one moderate-confidence recommendation, for urban and transport design and planning policies, which collectively have a clear evidence base showing that making the built environment and infrastructure more amenable to physical activity, including active commuting, can reduce physical inactivity.

We find mixed evidence, and make a low-confidence recommendation, for interventions specifically targeted at schools or workplaces, though these are clearly important components for any whole-of-community approach to reducing physical inactivity (for example, transport policies to increase cycling to work will benefit from adequate workplace cycle racks and shower facilities).

#### Hypertension

Based on the evidence we appraised, we make two recommendations specifically for hypertension; however, the evidence base for both reformulation (high-confidence) and public food procurement (moderate-confidence) interventions comes from a mix of studies considering sodium content specifically, including two Cochrane reviews (Supplementary Table 2A), and others which consider dietary sodium interventions as part of broader dietary policy (Supplementary Table 2B).

#### Diabetes

We make no specific recommendations for population-level interventions with empirical evidence showing a reduction in diabetes. However, obesity and physical inactivity are established risk factors for type II diabetes (T2DM), and we can therefore expect the interventions for obesity and physical inactivity to convey indirect dementia risk reduction benefits through a reduction in T2DM prevalence.

#### Depression

We did not make any high- or moderate-confidence recommendations for population-level interventions to reduce depression, despite identifying several high-quality reviews, including Cochrane reviews, which had looked directly for relevant evidence. The evidence base suffers from limitations around outcome ascertainment, with many studies considering ‘depressive symptoms’ measured by a single-question on a survey, rather than comprehensive clinical assessment.

We found mixed results for interventions aiming to improve social determinants of health (SDOH), such as social security interventions, housing interventions, and direct cash transfers (Supplementary Table 3A and B) – with some evidence that these interventions can reduce depression, but the evidence base was inconsistent and it was not clear which factors drive a successful, rather than null, outcome. We therefore grade this as a low-confidence recommendation. Through professional networks we identified one study, too recent to be included in any review articles, that demonstrated a reduction in anti-depressant prescribing (objectively, using electronic health records) in a deprived area of the UK. The intervention was the introduction of a community wealth building (CWB) programme which involved working with local anchor institutions to address upstream economic inequalities through measures like equitable distribution of investments and procurement, and commitment to paying a ‘living wage’ to all employees.^20^

#### Social isolation

We did not make any recommendations for population-level interventions to reduce social isolation – despite identifying several recent systematic reviews, including some that specifically searched for evidence on population-level interventions.

#### Traumatic brain injury

Clear evidence demonstrates the effectiveness of legislation to mandate the use of bicycle helmets for children, and motorcycle helmets for passengers of all ages, in reducing TBI. Further evidence, from HICs, suggested that provision of bicycle helmets to children from low socioeconomic backgrounds, school-based and community-based programmes to encourage helmet use, and proper enforcement of the legislation, will increase the likelihood of success of these recommendations.

More broadly, there is clear evidence, summarised through a series of articles from WHO, for road safety interventions to reduce the incidence of road traffic injuries (of which head injuries will constitute a proportion), these include speed management, segregated off-road cycle lanes, seatbelt legislation, and drink driving legislation (Supplementary Table 3A).

#### Low educational attainment

A Campbell Collaboration review reports robust evidence, particularly from Sub-Saharan Africa, for the removal of financial and food barriers from school (predominantly primary school) attendance. These include national policy to remove school fees, household cash transfers conditional on school attendance, and provision of free school lunches with/without take home rations (Supplementary Table 3D).

Several reviews identify empirical evidence from Europe that raising the mandated school leavers age (post-intervention leaving age ranged between 14 and 18 years in the included studies) increases the total amount of education received by the population, provided there is sufficient capacity within the education system.

Several of the studies reported equity impacts of these three recommendations, with children from lower income households standing to gain the most.

#### Air pollution

We make two recommendations for ambient air pollution, with clear evidence demonstrating a reduction in pollutants such as particulate matter (PM2.5 and PM10) and carbon monoxide (CO) from urban traffic restrictions, such as low emission zones, and postponement of non-essential polluting activities, such as road cleaning, on high-pollution days.

We make one recommendation for indoor air pollution, with consistent evidence from a WHO systematic review reporting empirical reductions in PM2.5 and CO resulting from stove exchange programmes that replaced traditional cookers using biomass with newer devices using cleaner fuels. A second review by WHO also reported key contextual factors to consider when implementing this type of intervention, including the provision of stove maintenance where required, and ensuring traditional foods can be cooked effectively on the newer stoves (Table 3).

#### Hearing impairment

We found clear evidence that occupational interventions involving exchanging equipment for quieter alternatives, and provision of (and mandated use of) hearing protective devices for those working in consistently noisy environments can reduce hearing loss (Supplementary Table 3A).

No evidence was found for policy interventions to make hearing corrective devices more readily available or affordable to the general public.

## Discussion

We identified clear and robust evidence for the effectiveness of 26 population-level interventions to reduce the prevalence of nine of the 12 risk factors, of which 23 have been empirically evaluated in HICs, and 16 in LMICs. We identify interventions that act through fiscal (e.g., removing primary school fees), marketing/advertising (e.g., plain packaging of tobacco products), availability (e.g., cleaner fuel replacement programmes for cooking stoves), and legislative (e.g., mandated provision of hearing protective equipment at noisy workplaces) levers.

The evidence base was more mature with regards to some risk factors, and some intervention types, than others. We were unable to include any recommendations in our framework for diabetes (directly), depression or social isolation. For depression, we did identify a developing evidence base for SDOH-type interventions such as community wealth building, social welfare policies, and housing improvements, however, the evidence base was not yet strong enough for inclusion in our framework. For social isolation, we identified several recent systematic reviews that have specifically looked for evidence on population-level interventions, for example urban green space interventions, and found a paucity of high-quality evidence. Given the importance of depression and social isolation in their own right, as well as being modifiable risk factors for dementia, these represent significant evidence gaps.

The evidence bases for the fiscal, legislative, and availability intervention types were relatively more mature than for marketing and advertising restrictions. However, we note this is an increasing area of research,^21^ and the quality of evidence may be expected to improve in the near future.

As outlined in the introduction, primary prevention can be characterised through a binary distinction between individual-level and population-level approaches. However, it is significant to note that our review of existing NCD prevention frameworks (Supplementary Table 4) identified one framework in particular^22^ that conceptualised the types of interventions included in this review as an intermediate level between superficial, individual-level interventions, and more radical, societal-level change. This speaks to an intractable trade-off between the relative ease of implementation and evaluation of an intervention, and the profoundness of the scale of change that can be achieved. To take obesity as an example, appetite-suppressant drugs are readily amenable to clinical trials to demonstrate efficacy, but depend on healthcare access and drug affordability, and do not address the root causes of obesity, so will have limited impact on obesity prevalence and will increase health inequalities. At the other end of the spectrum, making significant, sustained (without the need for ongoing intervention) changes to the food system, such that healthier products are more available, affordable, and socially desirable than unhealthy alternatives, is difficult to achieve, and even more difficult to evaluate directly through changes to individuals’ body mass index. In this context, the population-level interventions identified by this review (e.g., taxation on sugar-sweetened beverages) can be seen as a pragmatic middle-ground, enabling policymakers to achieve meaningful, and equitable, reductions in population dementia risk, with support from empirical evidence of effectiveness.

Our complex evidence review approach enabled us to identify evidence on the best-researched population-level interventions, across 12 different risk factors, identifying evidence relevant to all levels of government, in both HICs and LMICs. This approach assumed causality of the risk factors on dementia, and none of the included articles measured a change in dementia prevalence directly. In addition, for obesity, we assumed that measured reductions in total caloric or free sugar intake would reduce weight. Due to the complexity and late-life nature of the dementia syndrome, proving beyond doubt the causality of a dementia risk factor is challenging. The evidence bases of the 12 risk factors we included have been adjudged by the Lancet's commission of experts to be sufficiently robust to consider them as potentially modifiable risk factors. In addition to the potential dementia risk reduction benefit, each of these risk factors also represent valid targets for public health policy in their own right.

By incorporating evidence from existing NCD prevention reviews and frameworks, we were able to meaningfully synthesise this evidence into a user-friendly framework, which can help to structure policymaking approaches to population-level dementia risk reduction. Given the breadth of our research question, it was not feasible to conduct a systematic review of primary evidence, and pilot searches confirmed that even an umbrella review would have produced an unmanageable number of potential studies. Moreover, a systematic review aims to identify all literature on a topic, whereas policymakers (and the professionals who work with them) really want to know what the best-evidenced interventions are, and how to implement them^15^^,^^23^ – we designed our study accordingly. Our approach may have missed some relevant interventions, particularly those from emerging evidence bases which may not be well covered by secondary data sources like Cochrane and WHO yet. For example, research since the Lancet commission's 2020 report has suggested strengthening evidence of a causal link between repeated sports-related concussions (mild TBI) and dementia risk^24^ and some policy-level interventions such as rule changes to minimise collisions have been identified.^25^^,^^26^ It will be relevant to periodically update our review to capture these emerging evidence bases.

We included key contextual information for each policy recommendation (Table 3) but we did not explicitly consider negative effects of the policy recommendations, such as higher tax burden or reduced autonomy. We only include recommendations in our framework that were judged to be high- or moderate-confidence. These criteria are clear and were developed specifically for this review, and gradings for each recommendation were agreed between three co-authors (SW, LW, NM), nevertheless there is some subjectivity in these judgements.

Whilst it is easy to conceptually distinguish population-level interventions from individual-level ones, in practice there are interventions that blur this boundary, and definition of ‘population-level interventions’ vary.^27^ We focused our population-level intervention definition on interventions that change societal conditions, leading to the exclusion of some interventions, such as screening, vaccination, and mass media interventions, which are delivered at scale, but are clinical or high-agency in their mechanism. This approach ensured that all our recommendations are likely to achieve the key advantages of the population-level approach as described by Geoffrey Rose^10^: magnitude, equity, and longevity of benefit.^12^ The recommended interventions will typically benefit the population across the lifecourse (e.g., reduction of pollution benefits everyone from children to older people), however, as evidence strengthens that specific lifecourse phases are important for dementia risk accumulation, targeting of some interventions towards specific population subgroups may be possible.

We included only interventions for which there were empirical data on showing beneficial changes in risk factors for dementia. Study designs included randomized control trials, quasi-experimental studies, and natural experiment designs. Due to the complex nature of population-level interventions, it was rarely feasible for studies to eliminate all possible sources of bias. The limitations of the included studies were considered when grading evidence. Forms of evidence beyond these designs are clearly important to a comprehensive understanding of disease and prevention strategies, including observational, qualitative, and modelling data. However, there are potentially unknowable factors when applying these non-empirical data to policy implementation, and direct interventional evidence holds a tangible advantage when communicating with policymakers, which drove our decision to prioritize this evidence. Where relevant, we considered the supporting evidence from other types of data, and this is described explicitly in the evidence tables. And we provide policymakers with key implementation guidance and contextual information for the recommendations, in Table 3.

Policymakers, public health leaders, and dementia researchers aiming to reduce dementia are presented with two broad approaches, identification of high-risk individuals and encourage them to adopt healthier lifestyles and/or take up clinical interventions (individual-level approaches), or the introduction of policies and interventions that make societal conditions less conducive to the development of dementia (population-level approaches). Population-level approaches have the greatest reach and can achieve the largest, sustained, and equitable reductions in disease. Nevertheless, individual-level approaches have dominated policy and research spheres because they are considered easier to implement and evaluate.^11^^,^^14^ In this review, we have made 26 recommendations for interventions with strong empirical evidence that they can reduce dementia risk factors across the population. In addition, we provide key accompanying information and resources that policymakers can use to implement these recommendations in their own context. Whilst more research is needed, in particular concerning population-level interventions to reduce depression and social isolation, the central message of this review is that we already have enough evidence to tackle a major global public health challenge. The policies and interventions recommended have relevance at all levels of government, in both HIC and LMICs – the time for policy action is now.

### Outstanding questions

• Identification of effective population-level interventions to reduce depression and social isolation.
• Further causal epidemiology work to develop the evidence base for, and identify new, modifiable risk factors for dementia.
• Interventional evidence and natural experiment studies to examine the effect of population-level interventions to reduce these risk factors on directly measured dementia incidence, where feasible.

## Contributors

All authors conceived this paper. SW, LW, IK, and NM developed and piloted the search strategies. SW, LW and NM accessed and verified the data, and performed article screening, study selection, data extraction and checking, quality assessment, and evidence grading. SW drafted this paper. All authors reviewed and edited this paper.

## Data sharing statement

No new data were collected for the purposes of this review. All evidence synthesised in this review is available from the cited references.

## Declaration of interests

We declare no interests.
