# Supplementary material for: Population-level interventions for the primary prevention of dementia: a complex evidence review
Source: eClinicalMedicine. 2024 Mar 10;70:102538. doi: 10.1016/j.eclinm.2024.102538 (PMC10940136; doi:10.1016/j.eclinm.2024.102538)
Supplement: Supplementary data [file mmc1.docx]

**Population-level interventions for the primary prevention of dementia: a complex evidence review. SUPPLEMENTARY MATERIAL**

Contents

| Appendix A | Detailed search strategy | Pg 2 |
| --- | --- | --- |
| Supplementary Table 1A | Quality appraisal scores for general NCD prevention reviews | Pg 5 |
| Supplementary Table 1B | Extraction of general non-communicable disease prevention reviews | Pg 6 |
| Supplementary Table 2A | Extraction of Cochrane reviews from search (ii) | Pg13 |
| Supplementary Table 2B | Extraction of WHO evidence from search (ii) | Pg 30 |
| Supplementary Table 3A | Extraction of WHO and Cochrane evidence from search (iii) | Pg 63 |
| Supplementary Table 3B | Extraction of further searches for depression literature | Pg 94 |
| Supplementary Table 3C | Extraction of further searches for social isolation literature | Pg 97 |
| Supplementary Table 3D | Extraction of further searches for education literature | Pg 100 |
| Supplementary Table 4 | Extraction of existing NCD prevention frameworks | Pg 105 |

**Appendix A – Detailed Search Strategy**

### (i) Review of general NCD prevention literature – 12/01/23

**Rapid review of NCD prevention reviews**

MEDLINE via Ovid

Ovid MEDLINE(R) and Epub Ahead of Print, In-Process, In-Data-Review & Other Non-Indexed Citations, Daily and Versions 1946 to January 11, 2023

1. (review or systematic review).pt. or review*.ti. [limit to yr="2012 -Current”] – 1606184
2. (non-communicable disease* or noncommunicable disease*).ti. and prevent*.ti,ab. [limit to yr=”2012 -Current”] - 1080
3. 1 and 2 – 257

Advanced Google search

Pdf only. First 50 hits screened.

1. "Non-communicable disease" or "Non communicable disease" or "Noncommunicable disease"

AND

1. prevention

AND

1. review.

World Health Organisation (WHO)

Reviewed entire list of publications by the WHO noncommunicable diseases team, available here: <https://www.who.int/publications/i>

56 hits identified

Cochrane Library

1. "non communicable disease*"

AND

1. prevent*

8 hits identified

NICE

1. "non communicable disease prevention".

13 hits identified

Updated search 03/01/24, requested by peer reviewer

Medline search updated to 03/01/24. 57 new articles

Scopus search added:

(TITLE("non-communicable disease*" or "noncommunicable disease*") AND TITLE-ABS(prevent*) AND TITLE(review*)) or (TITLE("non-communicable disease*" or "noncommunicable disease*") AND TITLE-ABS(prevent*)) AND ( LIMIT-TO ( DOCTYPE,"re" ) ) AND ( LIMIT-TO ( PUBYEAR,2012) OR LIMIT-TO ( PUBYEAR,2013) OR LIMIT-TO ( PUBYEAR,2014) OR LIMIT-TO ( PUBYEAR,2015) OR LIMIT-TO ( PUBYEAR,2016) OR LIMIT-TO ( PUBYEAR,2017) OR LIMIT-TO ( PUBYEAR,2018) OR LIMIT-TO ( PUBYEAR,2019) OR LIMIT-TO ( PUBYEAR,2020) OR LIMIT-TO ( PUBYEAR,2021) OR LIMIT-TO ( PUBYEAR,2022) OR LIMIT-TO ( PUBYEAR,2023) OR LIMIT-TO ( PUBYEAR,2024) )

285 articles.

Web of Science Core collection search added:

# Web of Science Search Strategy (v0.1)

# Database: Web of Science Core Collection

# Entitlements:

- WOS.IC: 1993 to 2024

- WOS.CCR: 1985 to 2024

- WOS.SCI: 1900 to 2024

- WOS.AHCI: 1975 to 2024

- WOS.BHCI: 2008 to 2024

- WOS.BSCI: 2008 to 2024

- WOS.ESCI: 2019 to 2024

- WOS.ISTP: 1990 to 2024

- WOS.SSCI: 1956 to 2024

- WOS.ISSHP: 1990 to 2024

# Searches:

1: TI=( "non-communicable disease*" or "noncommunicable disease*" ) and (TI=(prevent*) or AB=(prevent*)) and TI=(review*) Date Run: Wed Jan 03 2024 14:31:11 GMT+0000 (Greenwich Mean Time) Results: 128

2: TI=( "non-communicable disease*" or "noncommunicable disease*" ) and (TI=(prevent*) or AB=(prevent*)) and Review Article (Document Types) Date Run: Wed Jan 03 2024 14:31:15 GMT+0000 (Greenwich Mean Time) Results: 253

3: #1 OR #2 and 2023 or 2022 or 2021 or 2020 or 2019 or 2018 or 2017 or 2016 or 2015 or 2014 or 2013 or 2012 (Publication Years) Timespan: 2012-01-01 to 2024-12-31 Date Run: Wed Jan 03 2024 14:33:38 GMT+0000 (Greenwich Mean Time) Results: 247

### (ii) Identification of population-level interventions for typical NCD risk factors – 22/03/23

**Focused search for interventions on tobacco, alcohol, diet and obesity, salt and hypertension, and physical inactivity**

WHO

The WHO periodically update the technical briefs for tobacco, alcohol, unhealthy diet, physical inactivity, diabetes, and cardiovascular diseases (including hypertension), available here (<https://www.who.int/teams/noncommunicable-diseases/updating-appendix-3-of-the-who-global-ncd-action-plan-2013-2030>) which underpin appendix 3 of the WHO Global NCD Action Plan. We reviewed the latest versions of these technical briefs (updated November 2022), as well as the technical annex to appendix 3 (updated December 2022), and accessed relevant strategy and technical documents referenced as sources of the interventions presented by these up-to-date technical briefs.

Cochrane Library

tobacco OR smok* OR alcohol* OR diet* OR "physical activity" OR "physical inactivity" OR obes* OR “unhealthy weight” OR "healthy weight" OR salt OR hypertension OR "blood pressure" OR diabetes in Record Title - with Cochrane Library publication date from Jan 2013 to present, in Cochrane Reviews (Word variations have been searched)

### (iii) Identification of population-level interventions for dementia-specific risk factors – 01/06/23

WHO Database - searches for article type: ‘publications’:

air pollution

brain injury

head injury

depression

social isolation

hearing loss

hearing impairment

education

Cochrane Library – searches for title/abstract/keyword (word variations auto-searched):

air pollution

brain injur*

head injur*

depression

mental health

social isolation

hearing loss

hearing impairment

education

school

Campbell Collaboration

Depression

Social isolation

**Further searches for Depression – 08/06/23**

TRIP Database (reviews or key primary evidence)

1. PICO search:
   I - population-level OR "population level" OR policy OR policies OR environment
   O - depression OR "mental health”
2. Default search: population-level intervention AND depression

Google Scholar (screened first 50 hits)

population-level intervention depression

policy intervention depression

"green space" AND intervention AND depression

Experts

Names of experts contacted available on request

Professional Networks

We also included articles identified through professional networks

**Further searches for Social Isolation – 13/06/23**

TRIP Database (reviews or key primary evidence)

1. PICO search:
   I - population-level OR "population level" OR policy OR policies OR environment
   O – “social isolation”
2. Default search: population-level intervention AND “social isolation”

Google Scholar

population-level intervention social isolation (screened first 50)

policy intervention social isolation (screened first 100)

Experts

Names of experts contacted available on request

Professional Networks

We also included articles identified through professional networks

**Further searches for Low Formal Education – 13/06/23**

TRIP Database (reviews or key primary evidence)

1. PICO search:
   I - population-level OR "population level" OR policy OR policies OR environment
   O – education OR school (reviewed first 50 SRs)
2. Default search: population-level intervention AND educational attainment

Google Scholar (screened first 50 hits)

population-level intervention education

policy intervention education

policy intervention educational attainment

policy intervention school completion

policy intervention higher education

Experts

Names of experts contacted available on request

Professional Networks

We also included articles identified through professional networks

| **Supplementary Table 1A – Quality appraisal scores (using AMSTAR 2) for general non-communicable disease prevention reviews** | | | | | | | | | | | | | | | | | | | |
| --- | --- | --- | --- | --- | --- | --- | --- | --- | --- | --- | --- | --- | --- | --- | --- | --- | --- | --- | --- |
| **Author, Year** | **1** | **2** | **3** | **4** | **5** | **6** | **7** | **8** | **9** | **10** | **11** | **12** | **13** | **14** | **15** | **16** | **# of critical weaknesses** | **# of non-critical weaknesses** | **Rating** |
| Aceves, 2019 | 1 | 0 | 0 | 0.5 | 0 | 0 | 0 | 0 | 0 | 0 | x | x | 0 | 0 | x | 1 | 4.5 | 6 | Critically low |
| Campbell, 2014 | 0 | 0 | 1 | 0.5 | 0 | 0 | 0 | 0.5 | 0 | 0 | x | x | 0 | 0 | x | 1 | 4.5 | 4.5 | Critically low |
| Fritz, 2022 | 1 | 0.5 | 1 | 0.5 | 1 | 0 | 0 | 0.5 | 0 | 0 | 0 | 0 | 0 | 1 | 1 | 1 | 4 | 1.5 | Critically low |
| Hyseni, 2017 | 1 | 0 | 1 | 0.5 | 1 | 0 | 0 | 0.5 | 0 | 0 | x | x | 0 | 0 | x | 1 | 4.5 | 2.5 | Critically low |
| Jain, 2022 | 1 | 1 | 1 | 0.5 | 0.5 | 1 | 0 | 0.5 | 1 | 0 | x | x | 1 | 1 | x | 1 | 1.5 | 1 | Low |
| Jourdan, 2016 | 1 | 0 | 1 | 0.5 | 1 | 1 | 0 | 1 | 0 | 0 | x | x | 0 | 0 | x | 1 | 4.5 | 1 | Critically low |
| Nguyen, 2023 | 1 | 0.5 | 1 | 0.5 | 1 | 1 | 0 | 0.5 | 1 | 1 | x | x | 0 | 0 | x | 1 | 3 | 1.5 | Critically low |
| Reilly, 2019 | 1 | 0 | 1 | 0 | 0 | 0.5 | 0 | 1 | 1 | 0 | x | x | 1 | 1 | x | 1 | 3 | 1.5 | Critically low |
| Rowbotham, 2017 | 1 | 0 | 1 | 0.5 | 0.5 | 0.5 | 0 | 0 | 1 | 0 | x | x | 1 | 0 | x | 0.5 | 2.5 | 3.5 | Critically low |
| Saraf, 2012 | 1 | 0 | 1 | 0 | 0 | 0 | 0 | 1 | 1 | 0 | x | x | 1 | 1 | x | 1 | 3 | 2 | Critically low |

| **Supplementary Table 1B – Extraction of general non-communicable disease prevention reviews** | | | | | | | | | | |
| --- | --- | --- | --- | --- | --- | --- | --- | --- | --- | --- |
| **Author, Year** | **Title** | **Summary of review** | **Risk factors/diseases targeted** | **Exclusive focus on population-level approaches?** | **Details of the population-level interventions** | **Populations/contexts of interest** | **Findings (overall and of the population-level interventions specifically)** | **Recommended interventions** | **Notes** | **Supportive evidence for broader review** |
| Aceves, 2019 | Non-communicable disease prevention in Mexico: policies, programs and regulations | Review of 9 federal NCD prevention plans(n=2), regulations (n=4) and/or policies (n=3) accompanied by scoping review to identify evaluation studies of their effectiveness | Almost exclusively healthy diet and obesity | Yes, exclusive focus on policies that target social and environmental factors | Healthy food provision in schools and workplaces, taxation of sugar-sweetened beverages and nonessential energy dense foods, clear front of package labelling, regulation of advertising of unhealthy food and drink to children, food security programmes | Whole population, schools, workplaces | 15% in sugar-sweetened beverages and 5.1% decrease in processed food consumption post-tax, poor compliance and inadequate enforcement of advertising regulation | Stops short of recommending specific policies, focussing more on the need for more funding to implement and evaluate further policies | Importance of adequate enforcement of advertising regulation to ensure compliance | Empirical evidence in favour of taxation of unhealthy foods. Supportive qualitative evidence for the acceptability of school food standard policies and front of pack labelling |
| Campbell, 2014 | Healthy food procurement policy: an important intervention to aid the reduction in chronic noncommunicable diseases  Healthy Food Procurement Policies and Their Impact | Review of 34 food procurement policy interventions in schools (n=20), worksites (n=6), and other settings (e.g. hospitals, care homes, and prisons) (n=6), remote communities (n=3) and measured healthier food consumption or purchasing, and nutrition-related health indicators | Poor diet, blood pressure, BMI, lipids, glucose | Yes, all interventions were changes to the food environment. Some interventions additionally included health education | A variety of interventions, including government regulation of food procurement standards, government subsidies for local producers of healthier foods, and site-based interventions to increase availability or affordability of healthier foods (and vice versa for unhealthy foods) in cafeterias and vending machines | Schools, worksites, hospitals, care homes, prisons, remote communities. All studies from HICs (mainly US, UK, Canada) | All studies showed a positive outcome. Most studies reported improvements in the healthiness of the average purchased or consumed meals. A small number of studies reported improvements in knowledge or attitudes towards healthy foods. Only two studies reported improved health outcomes: school-based interventions to increase availability of healthy foods in the cafeteria were shown to lower BP in one study, and BMI in another | Mandated healthy food procurement policies for schools, employers, and other places where food options are limited (e.g. hospitals, care homes, prisons, military bases, and remote communities). | Noted that interventions which included subsidising local food producers who produced healthy foods, such as fruit and vegetables, had a co-benefit of supporting local agriculture  No study evaluated the impact of these policies on food purchased outside of the setting of interest (e.g. workers may have adopted healthier foods at work, but started eating less healthily outside of work)  Some studies showed no change in the overall number of purchases (e.g. from vending machines), suggesting direct swapping of unhealthy with healthy choices rather than avoidance behaviours | Supportive evidence for procurement and service policies for diet - with evidence for schools, workplaces, hospitals, remote communities - which is currently graded as moderate. However, this review scored poorly on QA as there was no consideration of the risk of bias in the included studies |
| Fritz, 2022 | How to dampen the surge of non-communicable diseases in Southeast Asia: insights from a systematic review and meta-analysis | Systematic review and meta-analysis of 51 studies of the effect of interventions in South East Asia against smoking (n=8), alcohol (n=4), diet and physical inactivity (n=20), screening (n=4), health education (n=11), and (healthcare) systems approach (n=5) | Tobacco, alcohol, diet, physical inactivity, HbA1c, BMI, cholesterol | No, heavy focus on individual-level interventions. Population-level interventions made up 0/8 studies on smoking, 0/4 on alcohol, 2/20 on diet (Chawla, Koo), 0/13 on diabetes, 0/3 on CVD, and 0/4 on cancer  Chawla, 2017. Koo, 2018 | Multi-component school-based intervention including improving healthiness of food and drink options available at intervention schools, banning carbonated drinks, ask nearby vendors to offer healthier choices, increase classroom-based physical activity, and health education of children and parents  School-based provision of wholegrain foods, and health education of children and parents | South East Asia (excluding Singapore).  Population-level evidence from schools in Thailand and Malaysia | Initial meta-analysis showed a positive outcome, but pools across very heterogeneous study designs and outcome measures (using standardised mean differences). Clear evidence of publication bias, and results are more borderline after accounting for this, though still significant for screening interventions and patient education interventions.  Both population-level interventions demonstrated small but statistically significant improvements, in diet, physical activity, cholesterol and BMI in one study; and BMI and waist circumference in the other. |  |  | Supportive evidence for procurement and service policies in schools; and multi-components school-based physical inactivity interventions, but does not provide significant new evidence |
| Hyseni, 2017 | The effects of policy actions to improve population dietary patterns and prevent diet-related non-communicable diseases: scoping review | Scoping review of 58 reviews of empirical and modelling evidence on the effect of policy interventions to improve diet | Diet | Almost, few studies on nutrition education | Changing price of food, mostly taxation of unhealthy products, but some subsidisation of healthier products (n=19 studies), promotion of healthier foods through marketing (n=10), provision of healthier foods in schools or workplaces (n=11), reformulation policies (n=2), food labelling (n=6), supply chain investment (n=2) | All countries and settings included | Almost all interventions found to be effective. Evidence base most mature for price-based policies, taxing sugar-sweetened beverages and subsidising fruit and vegetables.   School- and workplace-based interventions to improve availability of healthy foods show moderate effects with a paucity of long-term data.   Food labelling and mass media interventions show moderate or null effects.   Paucity of evidence for food supply chain investment interventions. | Favours legislative action over softer levers. For example, banning junk food advertising to children more effective than mass media campaigns promoting healthy foods; taxation and mandating product reformulation more effective than voluntary schemes |  | Supportive evidence for fiscal policies (already graded as high confidence). Supportive of the moderate confidence grading for procurement and service policy for diet in schools and workplaces, due to lack of long-term data. Supportive of the moderate and low confidence grading for labelling interventions. Suggestive of possible inclusion of mass media interventions for diet, but suggests small to null effects |
| Jain, 2022 | Prevention and Management of High-Burden Noncommunicable Diseases in School-Age Children: A Systematic Review | Umbrella review of 50 SRs of interventions for the prevention and/or management of NCDs in children | Smoking, pollution | No, heavy focus on individual-level interventions. Only one study on reducing pollution relevant (Quansah 2017) | Installation of more efficient cooking stoves in LMICs (Malawi, Rwanda, Guatemala, Nicaragua, Nigeria, Burkina Faso, China, Peru, Kenya, Ghana, India, Nepal, Mexico, South Africa, Honduras, and Ethiopia) | Children | Reduction in particulate matter exposure |  |  | No |
| Jourdan, 2016 | The involvement of young people in school- and community-based noncommunicable disease prevention interventions: a scoping review of designs and outcomes | Scoping review of 12 intervention studies (1 RCT, others qualitative, non-randomised uncontrolled studies, or mixed methods) which evaluated the role of involving young people in school- and community-based NCD prevention interventions | Diet, physical inactivity | No, around half of the quantitative studies were population-level, others focused on health education | Increasing the healthiness of food offered by schools (n=4)  Construction of a playground n=1)  (Birnbaum 2002, Carissan 2012, Orme 2013, Rowe 2010) | Children | Scoping review findings are generally of a lack of evidence to answer their specific review question  Quantitative outcome data reported for only one of the population-level interventions (Bimbaum 2002) showing a small increase in fruit and vegetable consumption |  |  | One study provides supportive evidence for procurement and service policies in schools |
| Nguyen, 2023 | Cost-effectiveness of  non-communicable disease  prevention in Southeast Asia: a  scoping review | A scoping review of 42 cost-effectiveness analyses of interventions in South East Asia spanning primary, secondary and tertiary prevention programmes for CVD and type II diabetes | Risk factors: smoking, alcohol, physical inactivity, poor diet, hypertension, hyperlipidaemia.  Diseases: cardiovascular disease and type II diabetes | Mixed, several studies focus on population-level interventions but majority individual-level and/or clinical interventions | Taxation of cigarettes (n=4) or sugar sweetened beverages (n=2), ban on smoking in public places (n=3), regulation of tobacco advertising and/or warning labels (n=3), reformulation or substitution to reduce sodium intake (n=2) | South East Asian adults (excluding Singapore as high-income) | All population-level interventions considered likely to be cost-effective | Discouragement of tobacco use through taxation, warning on package; reducing salt intake through a government “soft regulation” strategy that combines targeted industry agreements, government monitoring,  and public education; reducing sugar consumption through effective taxation on  sugar-sweetened beverages | Analyses based on economic modelling, and the degree of empirical data driving these models compared to assumptions is not clear from the descriptions in the review | Economic modelling to support taxation, marketing, and reformulation policies in low- and middle-income countries |
| Reilly, 2019 | Physical activity interventions in early life aimed at reducing later risk of obesity and related non-communicable diseases: A rapid review of systematic reviews | Rapid umbrella review of 23 systematic reviews of interventions to increase physical activity amongst children | Physical inactivity, obesity | No, heavy focus on health education interventions | Provision of physical activity equipment (n=2), policy change at pre-school to mandate minimum 1 hour of structured exercise (n=1), improving the playground facility (n=1) | Children | Majority of interventions identified individual-level (despite aiming to cover the socioecological model). Overall quality of the evidence was low. Whilst several interventions were found to be efficacious, the authors note these were small effects and studies measured short-term outcomes only  Only the study measuring playground improvements reported a positive outcome | Multi-faceted interventions which address multiple lifecourse stages and multiple levels of the socio-ecological framework, with a particular focus on organisational/policy levels |  | Weak evidence, but supports the conclusion that interventions to increase physical activity require structural changes, such as playground improvements, rather than more superficial interventions |
| Rowbotham, 2017 | Effective whole-of-population strategies for preventing chronic disease | Rapid umbrella review of 48 high-quality reviews of 'whole of population' interventions for the prevention of chronic disease | Poor diet, physical inactivity, tobacco use, harmful alcohol consumption, obesity | Mostly, also included health education campaigns, and behavioural interventions delivered at scale (e.g. exercise on prescription) | Laws and regulations (e.g. junk food advertising restrictions), tax and price interventions (e.g. sugar-sweetened beverages tax), environmental and availability interventions (e.g. improving green space and cycling infrastructure) | Adults in high income settings comparable to the Australian population. Excluded interventions targeted at high-risk groups, e.g. Indigenous populations | Overall reported most consistent evidence for workplace-based interventions for diet, physical activity, weight, and smoking - with some of these interventions including built environment re-design, but mostly individual-level interventions.   Marketing restrictions, price-based policies, and community and workplace-based availability policies for food/sugary beverages/alcohol were found to have moderate evidence of effectiveness. Along with smoking bans and social marketing campaigns. |  | Did not report on strength of effect sizes or whether benefits sustained in the long-term. Only reported on 'strength of evidence', meaning consistency and maturity of evidence base demonstrating effectiveness. | Reports mixed evidence for many of the recommendations, however, due to the rapid review design, interpretation of findings from this review is challenging |
| Saraf, 2012 | A systematic review of school-based interventions to prevent risk factors associated with noncommunicable diseases | Systematic review of 37 school-based intervention studies (RCTs n=30; non-RCTs n=7) aiming to prevent NCD risk factors | Physical inactivity, diet, tobacco | No, majority of interventions were individual-level (e.g health education) | Multi-component school-based interventions that included improving the healthiness of the food available (n=4), or subsidising the cost of fruit and vegetable options (n=1)  (Bere 2006, French 2004, Lytle 2006, Foster 2008, Caballero 2003) - all US-based studies | Children. American Indian children (n=1) | Majority of included interventions effective and at low risk of bias (RCTs). However, many studies only measured knowledge or behaviour intention, and most had short follow-ups.  Only two population-level interventions reported significant positive behavioural effects, with small, short-term reductions in unhealthy food purchasing (French 2004) and overweight prevalence (Foster 2008). | School-based interventions |  | Provides supportive evidence for procurement and service-based policies for healthy diets in schools |

| Supplementary Table 2A – Extraction of Cochrane reviews from search (ii) | | | | | | | | | |
| --- | --- | --- | --- | --- | --- | --- | --- | --- | --- |
| Author, Year | Title | Risk Factor | Summary of Review | Focus on Population-Level Interventions | Details of the Population-Level Interventions | Populations/Context of Interest | Findings | Recommended interventions | Notes |
| Bala, 2017 | Mass media interventions for smoking cessation in adults | Smoking | 11 before-after studies with controls, assessing the effect of mass media interventions, with a primary intention of encouraging smokers to quit, on smoking prevalence | 3/11 studies considered the effect on sociocultural norms, measured as support for tobacco control legislation (n=2), and number of social contacts smoking and number encouraging individuals to quit (n=1) | Interrupted time series analyses of the (i) California and (ii) Massachusetts tobacco control programmes that ran through the 1990's, using population-representative state-wide repeat cross-sectional surveys.  (iii) Chi squared comparison of a mass media intervention targeted at Vietnamese-Americans in California, compared to a control group in Texas  All 3 considered at risk of bias due to non-randomised design and self-reported outcomes | Population-level interventions = adults in USA  Broader review: adults in Australia, England, South Africa, and USA | Norms concerning smoking changed compared to baseline in all three studies, but the only study with a control group found no difference between the intervention and control communities | n/a |  |
| Carson-Chahhoud, 2017 | Mass media interventions for preventing smoking in young people | Smoking | 8 studies, using clustered RCT designs (n=7), and an ITS design (n=1), assessing the effect of mass media interventions on smoking behaviours | 4/8 studies considered the effect on norms - e.g. perceived prevalence of smoking, and attitudes, amongst peers | School-based controlled trials in the USA. All considered at high-risk of bias due to randomisation approach or reporting, attrition, clustered design, and lack of blinding | Population-level interventions = school children in USA  Broader review: children in Norway and USA | Two studies found no evidence of effect, with either both groups and neither group reporting a change in norms. Small effects noted in favour of intervention in the other two studies | n/a |  |
| Coppo, 2014 | School policies for preventing smoking among young people | Smoking | 1 study, using a before-after design with control group, to assess the effect of a school-based intervention on smoking prevalence | 1/1 multi-component study with population-level and individual-level components | A school-wide smoking ban was combined with health education and training of anti-smoking champions. Considered at high risk of bias due to a small sample size, lack of blinding, intraclass correlation, and lack of reporting to determine selective reporting | School children in China | No difference in smoking prevalence at 1 year | n/a |  |
| Frazer, 2016a | Legislative smoking bans for reducing harms from second-hand smoke exposure, smoking prevalence and tobacco consumption | Smoking | 77 studies using ITS (n=36), before-after with control (n=23), and without control (n=18) investigating the health effects of legislative smoking bans in workplaces, restaurants and bars | Exclusively | Comprehensive' smoking bans (n=59 studies) - tobacco smoking prohibited in workplaces, including restaurants and bars. May include provision of smoking rooms  'Partial' smoking bans (n=18) - legislation which permits smoking in bars and restaurants  Included local, state or national level legislation | 21 countries (mix of HIC and LMIC): Argentina, Belgium, Canada, Denmark, England, France, Germany, Hong Kong, Ireland, Italy, Netherlands, New Zealand, Norway, Panama, Scotland, Spain, Sweden, Switzerland, Turkey, Uruguay, USA | Evidence for smoking bans in improving cardiovascular, respiratory, perinatal health, and mortality outcomes for both smokers and non-smokers is persuasive. Evidence of a dose‐response association, with sustained and improved health outcomes over time, specifically cardiovascular. Evidence that a comprehensive ban is more effective than a partial ban. Evidence of a benefit for lower socioeconomic groups. Given heterogeneity of evidence, singular estimated effect size not reported. Evidence demonstrating a reduction in smoking prevalence is mixed | Legislative smoking bans in workplaces, bars and restaurants |  |
| Frazer, 2016b | Impact of institutional smoking bans on reducing harms and second-hand smoke exposure | Smoking | 17 studies using before-after with control (n=3), and without control (n=14) investigating the second-hand health effects of smoking bans in hospitals (n=12), prisons (n=3), and universities (n=2) | Exclusively | Comprehensive or partial smoking bans in the setting of interest, in the presence of absence of broader public smoking bans already in place | Healthcare settings, prisons, and universities - both service users and staff  8 countries (mix of HIC and LMIC): Australia, Canada, Croatia, Ireland, Japan, Spain, Switzerland, and USA | Evidence of a reduction in active smoking prevalence in hospitals (RR 0.75 95% CI 0.69, 0.81)), and universities (RR 0.72 (0.64, 0.80)), with no effect noted in the prison setting - though enforcement of legislation in this setting was noted to be low. A reduction in passive smoking was reported in all three settings, either by self-report, or demonstrated health and mortality reductions | Legislative smoking bans in healthcare, further education, and prison settings | The overall quality of evidence was graded 'low' on account of study designs (the majority of studies lacked a control group), and because passive smoking rates were not biochemically validated |
| Lovato, 2011 | Impact of tobacco advertising and promotion on increasing adolescent smoking behaviours | Smoking | 19 longitudinal studies with 1 follow-up (n=11) or multiple follow-ups (n=8) assessing the association between exposure or receptivity to tobacco advertising, and the subsequent smoking behaviour | Exclusively - though the intervention is to increase the risk factor here, with assumed inverse effect of restricting advertising | 11 studies assessed receptivity to tobacco advertisement, for example the ownership or willingness to accept tobacco promotion items (e.g. branded t-shirts), or identification of a favourite brand or advertisement  5 studies assessed exposure to tobacco advertisement, for example via shopping frequency in stores containing cigarette advertising, or recall tests | Adolescents in HICs (Australia, England, Germany, Spain and USA) | There is substantial evidence that exposure to tobacco advertising is associated with adolescent smoking. Evidence that increased awareness or exposure to tobacco advertising was associated with an increased likelihood of tobacco initiation 6-24 months later was found in 18/19 studies, with odds ratios for high receptivity/exposure compared to low ranging from 1.1 to 2.89 (with a fairly even spread of effect estimates within this range). And evidence of a dose-response relationship with those considered in high-exposure/receptivity groups at increased risk than those at moderate-exposure/receptivity. Results supported by a consideration of available cross-sectional and time-series/econometric studies, but recognition that all these designs are at risk of confounding | Policies to restrict exposure of adolescents to tobacco advertising | This review does not provide evidence of advertising restrictions on a reduction in smoking (rather it shows that an increase in advertising increases smoking, with an assumption that the inverse will hold). |
| McNeill, 2017 | Tobacco packaging design for reducing tobacco use | Smoking | 51 studies using randomised (n=1), cohort or cross-sectional (n=19), or lab-based experimental (n=31) designs, investigating the effect of plain tobacco packaging interventions on smoking prevalence/ consumption (n=5) or behaviours (e.g. eye tracking) (n=46) | Exclusively - considering this as a marketing restriction | Packaging with a uniform colour (and in some cases shape and size) with no logos or branding, apart from health warnings and other government‐mandated information, and the brand name in a prescribed uniform font, colour and size. Designed to reduce the promotional appeal. | Only Australia had implemented the intervention at the time of study. Experimental RCT conducted in UK. | The observational study from Australia found a 3.66% reduction in odds following the introduction of the plain packaging legislation. This equated to an absolute drop in prevalence from 19% to 18.5%. This is supported by a second analysis of the same data by the Australian government, finding a 0.55% absolute reduction. This study was generally considered at low risk of bias, except for their outcome measure which may have biased towards the null (leading to an effect underestimate) and the possibility of confounding by a co-introduction of health warnings which were more graphic on the packaging  Findings supported by observational data on an increase in quit attempts, and experimental data on behaviours such as increased attention to health warnings when the rest of the packaging is plain | Plain packaging of cigarettes |  |
| Stead, 2005 | Interventions for preventing tobacco sales to minors | Smoking | 35 studies using randomised (n=6), non-randomised (n=7), and uncontrolled before-after studies (n=22) of interventions in improve shopkeeper’s adherence to legislation prohibiting the sale of tobacco to minors, mostly measured by compliance checks but sometimes measured by changes in prevalence | From the perspective of the children in these communities, all interventions considered here were population-level; though the most common intervention type was an education-based intervention with the shopkeepers | Education about legal requirements, notification of the results of compliance checks, warning of enforcement, implementation of enforcement, and co-introduction of other legislation such as restrictions of self-service displays and minors' possession of tobacco | Children in Australia, Canada, UK, and USA | Trials with control groups show mixed results regarding reductions in prevalence, with some reporting significant reductions in younger, but not older, age groups, and others finding no effect. Uncontrolled studies found reductions in prevalence by up to 10% (absolute reduction), but these studies were at higher risk of bias  Regarding compliance, those studies which used education only were generally ineffective. Whilst those which involved an active enforcement component were usually successful | Where legislation is in place to reduce tobacco sales to minors, active enforcement policies are required to achieve good compliance from shopkeepers | There was no evidence of the effect of the introduction of the legislation itself. The discussion notes several arguments postulated in favour and against this regulation. This policy is not included by the WHO, and there is insufficient evidence here to add it. |
| Siegfried, 2014 | Restricting or banning alcohol advertising to reduce alcohol consumption in adults and adolescents | Alcohol | 4 studies using RCT (n=1) and ITS (n=3) designs assessing the implementation of (n=3) or lifting of (n=1) advertising restrictions on alcohol production on alcohol consumption | Exclusively | RCT from the Netherlands randomised 40 men aged 18-29 to watch a movie with either high- or low-alcohol content, accompanied by an alcohol advertisement (3 intervention groups); control group watched low-alcohol content + neutral advert. Consumption of alcohol during viewing session used as the outcome. Considered at risk of bias due to lack of blinding of staff and participants  3 ITS studies all based in Canada and tracked alcohol sales data in provinces introducing (n=2) or lifting (n=1) a ban, with use of a neighbouring province control group. All considered at risk of bias due to the non-randomised design. Additionally, one intervention was noted to have occurred as a direct policy decision by a government who lost office during the implementation - these socio-political factors were considered to be a possible source of confounding during the analysis period. This study also lacked some consumption data for certain types of alcohol | Young male trial participants in the Netherlands, and general population in Canada | RCT found that watching a high-alcohol movie and seeing an alcohol advert resulted in a statistically significant increase in alcohol consumption (MD 0.65 and 0.73 drinks respectively). Generalisability of these findings unclear  The ITS studies generally all found no statistically significant effects of bans on alcohol consumption. One study found an increase in beer and wine purchases after lifting of a ban, but this was compensated for by a decrease in spirits, producing a non-significant effect overall | Lack of robust evidence to support |  |
| Crockett, 2018 | Nutritional labelling for healthier food or non‐alcoholic drink purchasing and consumption | Diet/Obesity | 28 studies using RCT (n=16), cluster RCT (n=1), quasi-RCT (n=5), and ITS (n=6) designs to evaluate the effect of nutritional labelling of menus or food/drink packaging on purchasing and consumption of healthy and unhealthy foods | Exclusively - considering this as a marketing restriction | Most studies (n=20) evaluated labelling on menus or menu boards, where a range of products were available. The remaining (n=8) provided only one food/drink option, but included a nutritional label on the packaging. Some (n=12) assessed absolute energy labels, with no other information/formatting, others (n=4) assessed traffic light formats, or energy labels with further information on nutritional content or exercise equivalents (n=6). Studies generally scored well on risk of bias assessments, with most bias concerns arising from randomisation method and lack of blinding | Conducted in a lab-based/artificial setting (n=17), or real-world settings (e.g. cafeterias and coffee shops) (n=11). Artificial settings generally involved university campus participants, whilst real-world studies studied the general population  All studies were conducted in high income countries (USA, Canada, UK, Netherlands). | Meta-analysis of the restaurant-based RCTs found a reduction in energy purchased of 47kcal (95% CI -15,-78) equating to a relative reduction of 7.8% (2.5% - 13.1%). Evidence from other high-quality real-world studies, and the lab-based experiments, supported this finding | Tentative support for nutritional labelling on menus in restaurants. More evidence required to recommend in other settings, such as vending machines and grocery stores | Currently the subject of an updated review, with a narrower focus on energy labels only |
| Hollands, 2019 | Altering the availability or proximity of food, alcohol, and tobacco products to change their selection and consumption | Diet/Obesity | 24 availability (n=6) or proximity (n=18) RCTs for health and unhealthy foods, and sugar sweetened beverages (no studies identified for alcohol or tobacco products) | All trials tested an intervention which could be implemented as a population-level intervention, however, only 10 were conducted in real-world settings | Changing the proximity of food and drink (e.g. in lab-based settings) (n=14), changing the order of products encountered in a buffet line (e.g. placing vegetables first in a school canteen) (n=4), changing the relative proportion of healthy to unhealthy options (e.g. in a workplace vending machine) (n=4), changing the absolute availability of healthy foods (e.g. in school canteen) (n=2) | All studies came from high income countries. Workplaces, supermarkets, restaurants, and schools | Interventions generally found to be effective, with meta-analysis suggesting meaningful reductions in selection and consumption of unhealthy choices resulting from both availability and proximity interventions. No evidence of publication bias, but meta-regression did show greater effects of proximity interventions in trials at higher risk of bias. The large proportion of the proximity studies conducted in lab-based settings limited the real-world applicability of proximity-based interventions | Interventions that change the absolute and relative availability, and order of, healthy and unhealthy food and soft drink choices | N.B. a 2023 study by several of the Cochrane authors added to this evidence base with an RCT for alcoholic beverages: (Clarke, PLOS medicine, 2023) finding that online shoppers exposed to a great variety of non-alcohol options, and a smaller proportion of alcoholic options, meaningfully reduced their alcohol purchases |
| Lhachimi, 2020 | Taxation of the fat content of foods for reducing their consumption and preventing obesity or other adverse health outcomes | Diet/Obesity | 2 ITS studies of the effect of the Danish tax on saturated fat (in place 2011 - 2012) on purchases of fatty foods | Exclusively | Excise taxes of 16 Danish krone (£1.70; €2.10; $2.70) per kilogram of saturated fat, on foods with a saturated fat content >2.3% (including meat, full‐fat dairy products, animal fats, edible oils, and margarine). One study used panel data, the other supermarket sales data. Panel data study considered at risk of bias due to potential lack of representativeness of panel households. Supermarket sales data reported only on selected food items | Denmark, general population | Both studies showed a resulting significant decrease in purchasing of high-fat foods. The panel data showed a reduction of approximately 6g/person/day. The supermarket data showed a relative reduction in demand for high-fat minced meat by 10.6% and high-fat sour cream by 8.6%, with evidence of substitution to lower fat products (resulting in a 4.2% reduction in the saturated fat content of all minced meat purchased, and 5.8% for cream sales | Stop short of recommending this intervention due to evidence from only one policy, and lack of outcome data for change in total energy intake or health outcomes | The Danish tax, and planned sugar tax, were scrapped due to economic considerations |
| Pfinder, 2020 | Taxation of unprocessed sugar or sugar‐added foods for reducing their consumption and preventing obesity or other adverse health outcomes | Diet/Obesity | 1 ITS study of the effect of the Hungarian 'public health product tax' on purchases of sugar-added foods | Exclusively | Content-based tax on SSB (including concentrates, syrups, and energy drinks), chocolates, cocoa-powder, alcohol, and fruit preserves (including jam). Co-introduced with a tax on high-salt items (e.g. salted snacks and condiments). Energy drinks could also be taxed for high caffeine content, whilst chocolate and cocoa powder could be taxed for high cocoa content. Outcome from large panel survey of 10,000 households. Scored well on risk of bias assessment, with possible misclassification of the outcome assessment leading to a possible underestimation of the effect, and also flagging the co-interventional nature of the tax | Hungary, general population | Reduction in mean purchasing of taxed sugar-added foods of 4%, corresponding to 40g/Kg or purchases (SMD -0.040 95% CI -0.07, -0.01). No assessment of total energy intake or health outcomes | Stop short of recommending this intervention due to evidence from only one policy, and lack of outcome data for change in total energy intake or health outcomes | Excluded three studies which studied the effect of the Mexican "tax on nonessential energy-dense foods", which included an SSB tax, but was based on the caloric content of foods and not the sugar content. These evaluations showed a 5-6% relative reduction in taxed foods, with evidence of a health equity effect |
| Hollands, 2015 | Portion, package or tableware size for changing selection and consumption of food, alcohol and tobacco | Diet/Obesity  (Also n=3 studies on smoking) | 72 RCTs of interventions that manipulated the portion size, package size, or tableware size or shape (e.g. plate size) of food products (n=69) or cigarettes (n=3) on selection and consumption outcomes. No studies evaluated alcohol products | Exclusively - considering these are availability interventions which affect the physical conditions in the food (and tobacco) environment | Manipulation of portion size (n=32), package size (n=10), or tableware size (n=12), or tableware shape (n=3). The three tobacco studies all tested the effect of longer vs. shorter cigarettes  All studies RCTs, but many considered at unclear risk due to lack of reporting (e.g. of randomisation procedures, blinding procedures, or selective reporting) | Conducted in lab-based (n=50) or real-world (e.g. restaurants, or school/workplace cafeterias) (n=22) settings. Most studies conducted on adults (n=55), or children (n=16) only, with only n=1 study involving both  All studies conducted in high-income countries (USA, Canada, Belgium, Netherlands, UK, Australia, South Korea) | Meta-analysis of 58 studies found a small to moderate effect of portion, package, or tableware size on consumption (SMD 0.38, 95% CI 0.29, 0.46), with effects consistent for adults and children. Which would equate to an 8.5%-13.5% (144-228kcal) reduction in calories, if achieved across the whole diet  Meta-analysis of the three tobacco studies showed no effect of longer cigarettes on tobacco consumption (SMD 0.25 (-0.14, 0.65) | Reduce portion size, package size, and/or tableware size of food products | Likely to have greatest effect by reducing size at the larger end of the scale, than changes to already smaller portions/packages/tableware |
| Baker, 2015 | Community wide interventions for increasing physical activity | Physical Activity | 33 studies using cluster RCT (n=5), or controlled before-after designs (n=28) either through independent or longitudinal cohort samples on the effect of community-wide interventions to increase PA | 31/33 studies included components which could be considered as population-level | All interventions were multi-component, with a general sense that environmental changes formed a relatively small part of most interventions. Partnership working with local government or NGOs (e.g. to coordinate local walking groups) (n=29), environmental interventions (e.g. construction of new cycle lanes) (n=14), mass media and social marketing (e.g. publicising physical activity opportunities, attempts to increase the attractiveness of PA) (n=23), working with specific settings (e.g. workplaces, schools) (n=18). Increase in physical activity measured mostly by self-report. Biases mainly from lack of randomisation and blinding, and outcome ascertainment | General population  Mostly from HICs, but 8 from LMICs (China (n=5), Pakistan, Vietnam, and Iran | Few studies reported statistically significant increases in physical activity rates. Generally, the better designed studies showed no improvement in the primary outcome measure of physical activity at a population level. Meta-analysis not performed due to high heterogeneity. Few studies collected sociodemographic data.   Only one study at low risk of bias appeared to prioritise built environment changes as a major part of the intervention (Wilson 2014) - a cluster RCT with improvements to local walking infrastructure from African American communities accompanied by social marketing to raise awareness and encourage usage. This intervention resulted in an immediate increase in observed walkers from 40 to 400 walkers/month, with evidence of a reduced but sustained benefit (average 200 walkers/month) over 9 months follow up. However, assessment of increase in MVPA by accelerometers of a representative group of individuals found a non-significant effect (0.69 additional mins of MVPA/day (95% CI -0.14, 1.39) which reflect the challenge of measuring a small benefit across the population | The body of evidence in this review does not support the hypothesis that the multi‐component community wide interventions studied effectively increased physical activity for the population, although some studies with environmental components observed more people walking | Considers findings against previous reviews, which generally also found null or inconsistent results - including those that summarised studies with and without a control group. One review (Kahn, 2022) which is also cited by WHO, was noted to have found strong evidence in favour of community-wide interventions for PA, but Cochrane authors note that most of the included studies were excluded by them because of methodological weaknesses. Similarly, Cochrane authors comment on the cycling-based interventions (which WHO also draw from) but comment again on the methodological issues |
| Neil-Sztramko, 2021 | School‐based physical activity programs for promoting physical activity and fitness in children and adolescents aged 6 to 18 | Physical Activity | 89 RCTs which assessed the effect of school-based interventions on an objective measure of physical activity | 78/89 trials included some component which could be considered population-level, however, no trials made significant changes to the school built environment | Interventions which increased the time allocated for physical activity, through provision of after-school clubs, PA within lessons (not PE), or at recess (n=18); interventions which increased the intensity of PA within PE lessons (n=8); multi-component interventions, combining increase PA opportunity/intensity with health education components (n=45), of which some included provision of play equipment (n=6); multi-component interventions which included some change to the school environment (n=7), however, these were generally superficial changes such as establishment of activity stations, drawing walking trails onto the school map etc. | School children  Mostly from HICs, but a few studies from LMICs such as Albania, Ecuador, Mexico, and South Africa (n=1 each) | Interventions probably result in little to no increase in time engaged in MVPA (MD 0.73 mins/d (95% CI 0.16, 1.30)), and may lead to little to no decrease in sedentary time (MD ‐3.78 mins/d (‐7.80, 0.24)), may improve physical fitness reported as maximal oxygen uptake (VO₂max) (MD 1.19 mL/kg/min (0.57, 1.82)), and may result in a very small decrease in BMI z‐scores (MD ‐0.06 (‐0.09, 0.02)), and may not impact BMI expressed as kg/m² (MD ‐0.07 (‐0.15, 0.01)) | Given the variability of results and the overall small effects, school staff and public health professionals must give the matter considerable thought before implementing school‐based physical activity interventions |  |
| Brand, 2022 | Replacing salt with low‐sodium salt substitutes (LSSS) for cardiovascular health in adults, children and pregnant women | Hypertension | 26 RCTs which replaced salt with low-sodium salt substitutes and measured resultant changes to cardiovascular health-related outcomes | 4/26 trials followed population-level approaches | Introducing salt substitutes into kitchens of retirement/nursing homes (n=2)  Introducing salt substitutes into village food supply chains (n=2). Both studies additionally included health education campaign to promote low salt diets. One study additionally investigated the added benefit of providing a financial subsidy for the lower salt product  Studies considered at high or unclear risk of bias due to clustered nature of randomisation, and in some cases due to lack of reporting e.g. of blinding | Care home residents, rural villages in LMICs (Taiwan, China, Peru | Population-Level Findings Introducing LSSS into care homes resulted in reduced cardiovascular mortality at 2.5 years (rate ratio 0.61 (0.39, 0.96)) in one study (Taiwan) (high RoB), and reduced SBP at 3 years (-7.90mmHg (-3.65, -12.15)) but not DBP in another (China) (high RoB). Introducing LSSS into village supply chains reduced hypertension at 2.5 years (HR 0.45 (0.31, 0.65)) in one trial (Peru) (unclear RoB), and produced null results at 2.5 years in the other (China) (unclear RoB).  General Findings Meta-analysis showed statistically significant reduction in SBP -4.76 (-3.50, -6.01) and DBP -2.43mmHg (-1.36, -3.50) at up to 60 months, which was considered clinically non-important at the individual-level, but meaningful at the population-level. Similar findings for studies that reported on stroke, ACS, and CVD-related mortality; but there was not deemed to be enough evidence to make firm conclusions for these outcomes. No evidence of harm associated with the use of LSSS, for example, no meaningful change in hyperkalaemia. However, authors note that some trials excluded people at risk of hyperkalaemia (e.g. those with CKD), and overall the evidence is insufficient to confidently say there is no possibility of harm in general population | Specific interventions not recommended | A small number of included studies were cluster randomised at the household or village level, but do not meet our definition of population-level interventions because they simply provided those in the intervention group with the substitute (there was no change to the societal conditions which would be maintained after the intervention). Whereas, the included studies were considered to be population-level affected the supply chains of the villages/hospital/nursing home, such that the substitutes would continue with ongoing intervention from the researchers |
| McLaren, 2016 | Population‐level interventions in government jurisdictions for dietary sodium reduction | Hypertension | 53 articles pertaining to uncontrolled before-after analyses of the effect of 15 national initiatives (e.g. product reformulation) for dietary sodium reduction on salt intake | 12/15 countries included a population-level component (the other 3 employed health education campaigns only) | Population-level components included: reformulation policies (n=11), procurement policies for specific settings (e.g. schools) (n=9), nutritional labelling policies (n=8), and restriction of marketing to children (n=1)  All studies considered at risk of bias due to uncontrolled design, but generally scoring well otherwise | General population, and specific settings such as schools, workplaces, prisons, and hospitals  Mostly HICs (Australia, Canada, China, Denmark, Finland, France, Ireland, Japan, Netherlands, NZ, Switzerland, Thailand, Turkey, UK, US) | Mixed results, including when focusing only on those countries which included 'structural' policies (reformulation and procurement), with only half of the studies showing significant reductions in sodium consumption. No meta-analysis due to heterogeneity. Evidence suggests that dietary sodium reduction is possible through government intervention | Population‐level interventions in government jurisdictions for dietary sodium reduction, particularly multi‐component (more than one intervention activity) and those of a structural nature (e.g. food product reformulation) |  |

| **Supplementary Table 2B – Extraction of the WHO evidence from search (ii)** | | | | | | | | | | | | | | | | | | |
| --- | --- | --- | --- | --- | --- | --- | --- | --- | --- | --- | --- | --- | --- | --- | --- | --- | --- | --- |
| **Article** | | **Description** | | **Recommended population-level interventions (grey indicates an indication that may or may not be population-level)** | | | | | | | | | | **Process followed** | | **Cited sources** | | **Notes** |
|  |  |  |  | **Tobacco** | | | **Alcohol** | | **Diet** | **Physical Inactivity** | | **Other** | |  |  |  |  |  |
| (2013) Global Action Plan Appendix 3 | | [Appendix 3] Menu of policy options and cost-effective interventions for prevention and control of major NCDs | | Very Cost-Effective Reduce affordability of tobacco products by increasing tobacco excise taxes  Create by law completely smoke-free environments in all indoor workplaces, public places and public transport  Ban all forms of tobacco advertising, promotion and sponsorship  Warn people of the dangers of tobacco and tobacco smoke through effective health warnings and mass media campaign | | | Very Cost-Effective Regulating commercial and public availability of alcohol  Restricting or banning alcohol advertising and promotions  Using pricing policies such as excise tax increases on alcoholic beverages  Also Recommended Supporting communities in adopting effective approaches and interventions to prevent and reduce the harmful use of alcohol | | Very Cost-Effective Replace trans fats with unsaturated fats  Also Recommended Implement recommendations on the marketing of foods and non-alcoholic beverages to children  Replace saturated fat with unsaturated fat  Manage food taxes and subsidies to promote healthy diet | Also Recommended To provide more convenient, safe and health-oriented environments for physical activity | | Very Cost-Effective Reduce salt intake | | [Action Plan] The global and regional consultation process to develop the action plan engaged WHO Member States, relevant United Nations system agencies, funds and programmes, international financial institutions, development banks and other key international orgs, health professionals, academia, civil society and the private sector through regional meetings organized by the six WHO regional offices, four web consultations which received 325 written submissions, three informal consultations with Member States and two informal dialogues with relevant nongovernmental organizations and selected private sector entities.  [Appendix 3] Unclear. "Current scientific knowledge, available evidence and a review of experience on prevention and control of NCDs" (N.B. Personal Correspondence from WHO - process was (1) set criteria for what data must be available on intervention's effectiveness for inclusion, (2) engage experts to suggest interventions which would meet these criteria, (3) review evidence against criteria and determine eligibility, (4) analyse using WHO-CHOICE methodology to determine cost-effectiveness - "very cost-effective = generate an extra year of healthy life for a cost that falls below the average annual income or GDP per person"). | | *Scaling up action against NCDs: How much will it cost? (WHO, 2011)*  *WHO-CHOICE* website  *Disease control priorities in developing countries (Disease Control Priorities Project, 2006)* - reference unclear, assume referencing this textbook, which summarises a lot of relevant evidence, but unclear how it was compiled  *Framework Convention on Tobacco Control* *(WHO, 2003).*   *WHO global strategy to reduce harmful use of alcohol (WHO, 2010)*.  *WHO Global Strategy of Diet, Physical Activity, and Health (WHO, 2004).* Developed by stakeholder engagement. Evidence summary is not referenced and no methodological information provided | | The interventions are to be delivered within a broader framework of an integrated health and development agenda - there a several overarching recommendations for supportive interventions on governance, advocacy, surveillance, and accountability |
| (2017) Appendix 3 (Best Buys Report) | | Provides policymakers with a list of ‘best buys and other recommended interventions to address noncommunicable diseases (NCDs) based on an update of Appendix 3 of the Global Action Plan to take into consideration the emergence of new evidence of cost-effectiveness and the issuance of new WHO recommendations that show evidence of effective interventions | | Best Buys Increase excise taxes and prices on tobacco products  Implement plain/ standardized packaging and/or large graphic health warnings on all tobacco packages  Enact and enforce comprehensive bans on tobacco advertising, promotion and sponsorship  Eliminate exposure to second-hand tobacco smoke in all indoor workplaces, public places, public transport  Implement effective mass media campaigns that educate the public about the harms of smoking/tobacco use and second hand smoke | | | Best Buys Increase excise taxes on alcoholic beverages  Enact and enforce bans or comprehensive restrictions on exposure to alcohol advertising (across multiple types of media  Enact and enforce restrictions on the physical availability of retailed alcohol (via reduced hours of sale)  Other (no CER) Carry out regular reviews of prices in relation to level of inflation and income  Establish minimum prices for alcohol where applicable  Enact and enforce an appropriate minimum age for purchase or consumption of alcoholic beverages and reduce density of retail outlets  Restrict or ban promotions of alcoholic beverages in connection with sponsorships and activities targeting young people  Provide consumer information about, and label, alcoholic beverages to indicate, the harm related to alcohol | | Other (CER >I$100) Eliminate industrial trans-fats through the development of legislation to ban their use in the food chain  Reduce sugar consumption through effective taxation on sugar-sweetened beverages  Other (no CER) Implement subsidies to increase the intake of fruits and vegetables  Replace trans-fats and saturated fats with unsaturated fats through reformulation, labelling, fiscal policies or agricultural policies  Limiting portion and package size to reduce energy intake and the risk of overweight/obesity   Implement nutrition labelling to reduce total energy intake (kcal), sugars, sodium and fats  Implement mass media campaign on healthy diets, including social marketing to reduce the intake of total fat, saturated fats, sugars and salt, and promote the intake of fruits and vegetables | Best Buys Implement community wide public education and awareness campaign for physical activity which includes a mass media campaign combined with other community based education, motivational and environmental programmes aimed at supporting behavioural change of physical activity levels  Other (no CER) Ensure that macro-level urban design incorporates the core elements of residential density, connected street networks that include sidewalks, easy access to a diversity of destinations and access to public transport  Implement whole-of-school programme that includes quality physical education, availability of adequate facilities and programs to support physical activity for all children  Provide convenient and safe access to quality public open space and adequate infrastructure to support walking and cycling  Implement multi-component workplace physical activity programmes  Promotion of physical activity through organized sport groups and clubs, programmes and events | | Best Buys Reduce salt intake through the reformulation of food products to contain less salt and the setting of target levels for the amount of salt in foods and meals  Reduce salt intake through the establishment of a supportive environment in public institutions such as hospitals, schools, workplaces and nursing homes, to enable lower sodium options to be provided  Reduce salt intake through a behaviour change communication and mass media campaign  Reduce salt intake through the implementation of front-of-pack labelling  Other (no CER) Access to improved stoves and cleaner fuels to reduce indoor air pollution | | Updated following consultation, seeking examples of interventions using the following criteria: "(1) An intervention must have a demonstrated and quantifiable effect size, from at least one published study in a peer reviewed journal. (2) An intervention must have a clear link to one of the global NCD targets". Full consultation paper not available online anymore.  Interventions assessed using WHO-CHOICE methodology, and categories as:  (i) 'Best Buys' = average ≤ I$100/DALY averted in LMICs (ii) Effective, but average > I$100/DALY averted (iii) Effective, but insufficient data to conduct WHO-CHOICE analysis. | | Technical annex, and links to implementation guidance, no longer available online | | As above.   Also, stresses importance of considering factors beyond cost-effectiveness, e.g. effectiveness, affordability, implementation capacity, feasibility, according to national circumstances, and impact on health equity of interventions, and to the need to implement a combination of population-wide policy interventions and individual interventions  Also note, "Italy and the United States of America dissociated themselves from operative paragraph 1 of resolution WHA70.11 and did not endorse the updated set of best buys and other recommended interventions for the prevention and control of noncommunicable diseases. They stated, inter alia, that they believe that the evidence underlying certain interventions was not yet sufficient to justify their inclusion. They considered that the proposed interventions should also reflect the view that all foods could be part of an overall healthy diet." |
| (2022) Draft Updated Appendix 3 and Technical Annex | | Update of the 2017 version of the Appendix 3 of the WHO Global NCD Action Plan 2013-2020, now extended until 2030, considering new scientific evidence as well as new WHO recommendations, since the 2017 update | | No change from Best Buys document | | | No change from Best Buys document, except addition of following advice for the first recommendation ('increase excise taxes on alcohol beverages'): " levying taxes should be combined with other price measures, such as bans on discounts or promotions" | | Best Buys Reformulation policies for healthier food and beverage products  Front-of-pack labelling as part of comprehensive nutrition labelling policies  Public food procurement and service policies for healthy diets  Behaviour change communication and mass media campaign for healthy diets  Policies to protect children from the harmful impact of food marketing  Protection, promotion and support of optimal breastfeeding practices  Other (CER I$ 100-500) [Provisional - analyses ongoing] Reduce sugar consumption through effective taxation on sugar-sweetened beverages  Other (No CER) Subsidies on healthy foods and beverages (e.g. fruits and vegetables) as part of comprehensive fiscal policies for healthy diets  Menu labelling in food service to promote healthy diets (e.g. reduce total energy intake (kcal) and/or intake of sugars, sodium and unhealthy fats)  Limiting portion and package size to reduce energy intake and the risk of overweight/obesity | Best Buys Implement sustained, population wide, best practice communication campaigns to promote physical activity, with links to community-based programmes and environmental improvements to enable and support behaviour change.  Other (no CER) Implement urban and transport planning and urban design, at all levels of government, to provide compact neighbourhoods providing mixed-land use and connected networks for walking and cycling and equitable access to safe, quality public open spaces that enable and promote physical activity and active mobility  Implement whole-of-school programmes that include quality physical education, and adequate facilities, equipment and programs supporting active travel to/from school and support physical activity for all children of all abilities during and after school  Improve walking and cycling infrastructure ensuring universal and equitable access to enable and promote safe walking, cycling, other forms of micro mobility (e.g. wheelchairs, scooters and skates) by people of all ages and abilities  Implement multi-component workplace physical activity programmes  Provide and promote physical activity through provision of community-based (grass roots) sport and recreation programmes and conduct free mass participation events to encourage engagement by people of all ages and abilities | | Other (no CER) Access to improved stoves and cleaner fuels to reduce indoor air pollution | | Largely unchanged from 2017 process | | General/Other *- WHO menu of cost-effectiveness interventions for mental health (WHO, 2021) - Compendium of WHO and other UN guidance on health and the environment - WHO global air quality guidelines: particulate matter, ozone, NO2, SO2 and CO (WHO, 2021)*  Tobacco *- FCTC - WHO technical manual on tobacco tax policy and administration (WHO, 2021) - IARC handbook on effectiveness of tax and price policies for tobacco control (IARC, 2011) - Plain packaging of tobacco products: evidence, design and implementation (WHO, 2016)* Alcohol *- WHO global strategy to reduce harmful use of alcohol (WHO, 2010)* - The SAFER Technical package (2019) - Resource book on alcohol taxation (2017) - Manual on alcohol taxation (in press) - Reducing the harm from alcohol by regulating cross-border alcohol marketing, advertising and promotion (WHO 2022) - The WHO ASSIST package for hazardous and harmful substance use (WHO, 2010)  Diet *- WHO Global strategy on diet, physical activity and health (WHO, 2004) - WHO policy brief on reformulation of food and beverage products (WHO, 2022) - WHO global sodium benchmarks for different food categories (WHO, 2021) - REPLACE technical document on eliminating industrially produced trans-fat from the global food supply (WHO, 2021) - SHAKE the salt habit: technical package for salt reduction (WHO, 2016) - Policy brief on nutrition labelling (WHO, 2022) - Guiding principles and framework manual for front-of-pack labelling for promoting healthy diet (WHO, 2019) - Implementing nutrition labelling policies: a review of contextual factors (WHO, 2021) - What is the evidence on the policy specifications, development processes and effectiveness of existing front-of-pack food labelling policies in the WHO European Region? (WHO, 2018) - Policy brief on fiscal policies to promote healthy diets (WHO, 2022) - WHO manual on sugar sweetened beverage taxation policies to promote healthy diets (WHO, 2022) - Implementing fiscal and pricing policies to promote healthy diets: a review of contextual factors (WHO, 2021) - SSB taxes in the WHO European region: success through lessons learned and challenges faced (WHO, 2022) - Fiscal Policies for Diet and Prevention of Noncommunicable Diseases (WHO, 2016) - Protecting children from the harmful impact of food marketing: policy brief (WHO, 2022) - Set of recommendations on the marketing of foods and non-alcoholic beverages to children (WHO, 2010) - Framework for implementing the set of recommendations on the marketing of foods and non-alcoholic beverages to children (2012) - Tackling food marketing to children in a digital world: trans-disciplinary perspectives (WHO, 2016) - Regional action framework on protecting children from the harmful impact of food marketing in WPRO (WHO, 2020)* - Action framework for developing and implementing public food procurement and service policies for a healthy diet (WHO, 2021) - Implementing school food and nutrition policies: a review of contextual factors (WHO, 2021)  Physical Activity *- WHO Global strategy on diet, physical activity and health (WHO, 2004) - Global action plan on physical activity 2018–2030: more active people for a healthier world (WHO, 2018) - Promoting physical activity through schools: a toolkit (WHO, 2021) - ACTIVE: a technical package for increasing physical activity (WHO, 2018) - Global recommendations on physical activity for health (WHO, 2010) - Promoting physical activity in the Eastern Mediterranean Region through a life-course approach (WHO, 2014)* | | The Secretariat identified a revised list of 83 interventions (excluding overarching/enabling actions). These include: 31 interventions which have been unchanged from the 2017 update and for which key parameters were updated and cost-effectiveness estimates were generated, 9 interventions from the 2017 update which have been revised to reflect updates in WHO policy or scientific evidence, 8 interventions included in the 2017 update that had no analysis carried out at the time and for which cost-effectiveness analysis was performed for the 2022 update, 10 new interventions from new WHO guidance and tools, and 25 interventions that are considered priorities but have no cost-effectiveness analysis.  Reports that some interventions were not considered for WHO-CHOICE analysis because of methodological or capacity issues. Indicating that some interventions without a CER analysis may have sufficient data, but were deprioritised by the WHO team - no clear criteria for how this was decided. |
| **Relevant contextual WHO documents cited by the above documents** | | | | | | | | | | | | | | | | | | |
| Year, Title | | | | | | Notes | | | | | | | | | | | | |
| (2021) Saving lives, spending less: the case for investing in noncommunicable diseases | | | | | | Provides updated, and more detailed, cost-benefit workings for the Best Buys. Overall ROI 7:1 by 2030 in LMICs. More favourable ROI and effectiveness when more Best Buys implemented together. Also gives case examples of implementation and results, with some limited references to peer reviewed literature | | | | | | | | | | | | |
| (2011) Scaling up action against NCDs: How much will it cost? | | | | | | Presents costs data for the analysis of the interventions recommended by the original appendix. Cites data source as: 'Prevention and control of NCDs: priorities for investment. Discussion paper for the First Global Ministerial Conference on Healthy Lifestyles and Noncommunicable Disease Control. (WHO 2011)' which is not available online | | | | | | | | | | | | |
| (2003) Framework Convention on Tobacco Control | | | | | | Presents the strategy, and describes the recommended interventions. Does not report any empirical evidence, and does not report how the interventions were selected. | | | | | | | | | | | | |
| (2021) WHO technical manual on tobacco tax policy and administration | | | | | | Presents updated exploration, including empirical evidence, of tobacco tax policy and administration | | | | | | | | | | | | |
| (2016) Plain packaging of tobacco products: evidence, design and implementation | | | | | | Report predominantly serves as an implementation guide. Short section summarising the comprehensiveness of the evidence base, which is in support of plain packaging and health warnings being effective, but do not report effect sizes. | | | | | | | | | | | | |
| (2010) WHO global strategy to reduce harmful use of alcohol | | | | | | Evidence generated through a combination of stakeholder engagement and evidence review - the evidence review findings are described in general terms in the annex (no methodology information), with a commitment to a bibliography being available online, but this link is broken | | | | | | | | | | | | |
| (2022) Draft action plan (2022-2030) to effectively implement the global strategy to reduce the harmful use of alcohol as a public health priority | | | | | | Update of the 2010 action plan. Presents targets and recommended actions, but not evidence. | | | | | | | | | | | | |
| (2021) SAFER - A world free from alcohol related harms. The Technical Package | | | | | | SAFER package provides support for Member States in reducing the harmful use of alcohol by strengthening the ongoing implementation of the Global strategy to reduce the harmful use of alcohol and related docs (including the NCD GAP). Provides guidance on how to implement the recommendations, but not evidence | | | | | | | | | | | | |
| (2017) Resource tool on alcohol taxation and pricing policies | | | | | | Designing and implementing alcohol taxation and pricing policies are normally in the hands of finance personnel, who are usually concerned with public finance rather than public health perspectives. Thus, this resource tool is designed to inform public health personnel and non-economist alcohol control policy advocates how to facilitate effective discussions and negotiations with financial officers. The goal is to integrate public health perspectives into taxation and pricing policy design and implementation. | | | | | | | | | | | | |
| (2020) Alcohol pricing in the WHO European Region - update report on the evidence and recommended policy actions | | | | | | Provides evidence-base and guidance for alcohol taxation and the MUP. | | | | | | | | | | | | |
| (2022) Reducing the harm from alcohol by regulating cross-border alcohol marketing, advertising and promotion. A technical report | | | | | | Requested as a specific report as part of the process of updating the global action plan. Underpinned by a scoping review. Presents a large amount of contextual information, exploring how marketing has become cross-national, strategies used by the alcohol industry, legal aspects of cross-border jurisdictions, and the relationship between marketing exposure and attitudes. Also presents some evidence of prevention strategies, though states this is not intended as a comprehensive review of this. | | | | | | | | | | | | |
| (2004) WHO Global strategy on diet, physical activity and health | | | | | | Presents the strategy. Evidence described in generality | | | | | | | | | | | | |
| (2022) WHO policy brief on reformulation of food and beverage products | | | | | | Summarises the Gressier evidence review (used in the Best Buys calculation), as well as PHE report. Frames reformulation as a harm reduction strategy, with encouraging home-cooked high-quality food preferable, but reformulation pragmatic. | | | | | | | | | | | | |
| (2021) WHO global sodium benchmarks for different food categories | | | | | | Detailed recommendations, by food type, of sodium content targets. Also includes information on the methodology to collate them. No empirical evidence for interventions to reduce sodium content. | | | | | | | | | | | | |
| (2021) REPLACE technical document on eliminating industrially-produced trans-fat from the global food supply | | | | | | Predominantly an implementation guide, but includes a brief summary of international evidence. | | | | | | | | | | | | |
| (2016) SHAKE the salt habit: technical package for salt reduction (WHO, 2016) | | | | | | Implementation guide for salt reformulation | | | | | | | | | | | | |
| (2022) Nutrition labelling: policy brief | | | | | | Information and options for nutrition labelling policies, including policies on ingredient lists, nutrient declarations, supplementary nutrition information (e.g. front-of-pack labelling FOPL) and nutrition and health claims. Briefly describes evidence base for FOPL - almost all references assess knowledge/understanding/attitudes rather than reduction in purchasing - except one study from Chile (Taillie 2021) | | | | | | | | | | | | |
| (2019) Guiding principles and framework manual for front-of-pack labelling for promoting healthy diet | | | | | | Based on a 2015 technical meeting to review the available evidence and to compile various country experiences and lessons learned in developing and implementing FOPL systems. | | | | | | | | | | | | |
| (2021) Implementing nutrition labelling policies: a review of contextual factors | | | | | | Evidence review of the values, ethics, feasibility, and acceptability of FOPL initiatives. Reports on cost-effectiveness modelling of hypothetical interventions, but does not present any empirical data on effectiveness of schemes. | | | | | | | | | | | | |
| (2018) What is the evidence on the policy specifications, development processes and effectiveness of existing front-of-pack food labelling policies in the WHO European Region? | | | | | | Formal review process with relevant search strategy followed (conducted by The WHO's European Region team: The Health Evidence Network) - though not clear 2 reviewers independently screened, and no risk of bias assessment performed. Vast majority of literature on the effectiveness of FOPL is based on self-reported consumer preferences/responses/understanding, rather than behaviour. The majority of studies evaluating changes in behaviour are hypothetical modelling studies. One experimental study was reported (Crockett), randomising cinema customers to popcorn with nutritional labelling or not - finding no effect on consumption. | | | | | | | | | | | | |
| (2022) Policy brief on fiscal policies to promote healthy diets | | | | | | Includes brief review of evidence for healthy food subsidies, as well as evidence in support of SSB taxation. | | | | | | | | | | | | |
| (2022) WHO manual on sugar sweetened beverage taxation policies to promote healthy diets | | | | | | Evidence review and implementation guidance for SSB taxation | | | | | | | | | | | | |
| (2021) Implementing fiscal and pricing policies to promote healthy diets: a review of contextual factors | | | | | | Evidence review of the values, ethics, feasibility, and acceptability of fiscal policies for diet. Reports extensive evidence that both taxation and subsidies are estimated to be potentially cost-effective or cost-saving in hypothetical modelling studies | | | | | | | | | | | | |
| (2022) SSB taxes in the WHO European region: success through lessons learned and challenges faced | | | | | | Summarises the current level of implementation of SSB taxes in Europe, and lessons learned (e.g. opposition faced during implementation). No empirical estimates of effectiveness | | | | | | | | | | | | |
| (2016) Fiscal Policies for Diet and Prevention of Noncommunicable Diseases | | | | | | Meeting report from an expert meeting convened by WHO in 2015 to discuss evidence for fiscal policies for diet. Includes an evidence review and implementation guides for SSB taxation and healthy food subsidy | | | | | | | | | | | | |
| (2022) Protecting children from the harmful impact of food marketing: policy brief | | | | | | Summarises evidence base, current policy, and arguments/counterarguments to implementation of comprehensive marketing bans. Includes reviews of evidence showing changes in children's preferences/eating habits in response to marketing, but not empirical effects of restrictions | | | | | | | | | | | | |
| (2010) Set of recommendations on the marketing of foods and non-alcoholic beverages to children | | | | | | Sets out the recommendations. Brief summary of evidence points to reviews showing the widespread nature of advertising, and the effect it can have on preferences (not the effect of bans). | | | | | | | | | | | | |
| (2012) Framework for implementing the set of recommendations on the marketing of foods and non-alcoholic beverages to children | | | | | | Implementation guide for the above recommendations | | | | | | | | | | | | |
| (2016) Tackling food marketing to children in a digital world: trans-disciplinary perspectives | | | | | | Specifically summarises evidence and recommendations related to digital marketing of unhealthy foods. Evidence presented is generally supportive rather than empirical (e.g. children's use of digital media, digital media historically excluded from restrictions, evidence of using digital media to bypass TV restrictions, digital media affecting preferences). | | | | | | | | | | | | |
| (2020) Regional action framework on protecting children from the harmful impact of food marketing in WPRO | | | | | | Updated framework for implementation of the 2010 recommendations, with a specific focus on the Western Pacific WHO region | | | | | | | | | | | | |
| (2018) A Child Rights-Based Approach to Food Marketing: A Guide for Policy Makers | | | | | | WHO/UNICEF joint publication outlining the supportive evidence base (the same as for the above documents) and providing implementation support for policymakers | | | | | | | | | | | | |
| (2021) Action framework for developing and implementing public food procurement and service policies for a healthy diet | | | | | | Implementation guidance for public food procurement policy. Presents some empirical evidence from reviews. Also summarises supporting evidence and real-world case examples. | | | | | | | | | | | | |
| (2021) Implementing school food and nutrition policies: a review of contextual factors | | | | | | Evidence review of the values, costs, ethics, feasibility, and acceptability of school food nutrition policies. Presents some cost-effectiveness studies which report mixed results - this section is primarily presented through a financial, rather than effectiveness, lens. | | | | | | | | | | | | |
| (2004) WHO Global strategy on diet, physical activity and health | | | | | | Presents the strategy. Evidence described in generality | | | | | | | | | | | | |
| (2007) A guide for population-based approaches to increasing levels of physical activity: implementation of the WHO global strategy on diet, physical activity and health. | | | | | | Implementation guide for the 2004 strategy | | | | | | | | | | | | |
| (2018) Global action plan on physical activity 2018–2030: more active people for a healthier world | | | | | | Presents the action plan. Evidence described in generality throughout rather than in a specific section, but some references to empirical data | | | | | | | | | | | | |
| (2021) Promoting physical activity through schools: a toolkit | | | | | | Toolkit describing how the school environment can be used to develop, implement and evaluate strategies that promote physical activity and reduce sedentary behaviour among children and young people. Including some relevant empirical evidence. | | | | | | | | | | | | |
| (2018) ACTIVE: a technical package for increasing physical activity | | | | | | Represents the 2018 action plan in a slightly more action-focussed way. No evidence directly referenced | | | | | | | | | | | | |
| (2010) Global recommendations on physical activity for health. | | | | | | Set of recommendations for how much physical activity different age groups should engage in for good health, based on scoping review and expert consensus. Not policy recommendations | | | | | | | | | | | | |
| (2016) Physical activity strategy for the WHO European Region 2016–2025 | | | | | | Sets out strategy, no references to empirical evidence | | | | | | | | | | | | |
| (2014) Promoting physical activity in the Eastern Mediterranean Region through a life-course approach | | | | | | Applies thinking from the global strategy to the Eastern Med region. Evidence described in generality. | | | | | | | | | | | | |
| (2019) The power of cities: tackling noncommunicable diseases and road safety | | | | | | Presents recommendations and case studies, without specific references to evidence | | | | | | | | | | | | |
| (2018) Towards More Physical Activity in Cities | | | | | | Presents recommendations and case studies, with some references but not a formal evidence review | | | | | | | | | | | | |
| (2009) Interventions on diet and physical activity: what works: summary report. | | | | | | Formal evidence review of effectiveness of interventions to increase physical activity and improve diets. Included only primary studies No formal QA reported, but applied a judgement of confidence in the effectiveness of a group of interventions on a 4-point scale based on the totality of evidence. Review of primary evidence does not support the conclusions of robustness made by the authors | | | | | | | | | | | | |
| (2008) A healthy city is an active city: a physical activity planning guide | | | | | | Predominantly an implementation guide, but does contain some references to empirical data | | | | | | | | | | | | |
| (2006) Physical activity and health in Europe: evidence for action | | | | | | Makes the case for intervention, summarises policy options for action with some references to empirical evidence | | | | | | | | | | | | |
| (2006) Promoting physical activity and active living in urban environments. The role of local governments | | | | | | Makes the case for intervention, summarises policy options for action with some references to empirical evidence | | | | | | | | | | | | |
| (2005) Review of Best Practice in Interventions to Promote Physical Activity in Developing Countries | | | | | | Formal evidence review (SR + stakeholder work) to identify most effective interventions to increase physical activity in LMICs. Mostly focussed on process outcomes. Annex 3 contains references to empirical effectiveness data for some best practice PA interventions, but these are described in insufficient detail, without appropriate referencing to follow up. | | | | | | | | | | | | |
| **Best Buys and other recommended interventions** | | | | | | | | | | | | | | | | | | |
| Risk  Factor | Intervention | | | | Intervention Details and Caveats | | | WHO Implementation Resource(s) | | | Estimated Effectiveness | | Sources of Evidence of Effectiveness | | Other Sources of Evidence | | Notes | |
| Tobacco | Increase excise taxes and prices on tobacco products | | | | HLY estimate assumes an excise tax increase that causes retail prices to increase by 25%. 4% average reduction in LMICs, 5% in HICs.  Recognition that there are different types of taxation. Comprehensive assessment of this question available in WHO manual on tobacco tax policy (most recently updated 2021) which recognises that different countries have different taxation systems and should make appropriate decisions for their local context. However, excise taxes (specific taxes on tobacco products), which are regularly updated to keep up with inflation and progressively make cigarettes less affordable, are recommended as the most effective measure. Must be implemented alongside other work to ensure reductions in illegal tobacco trade which bypasses excise taxation. | | | *Framework Convention on Tobacco Control (WHO, 2003)  WHO technical manual on tobacco tax policy and administration (WHO, 2021)* | | | 1,717-3,093 HLY gained per year per million people  Price elasticity -0.4 to -0.5 (meaning a 10% increase in price results in an average 4-5% reduction in consumption) | | *Ortegón et al., Cost effectiveness of strategies to combat cardiovascular disease, diabetes, and tobacco use in sub-Saharan Africa and South East Asia: mathematical modelling study (BMJ, 2012) -* N.B. some of author group from WHO  *WHO Technical manual on tobacco tax administration. (WHO, 2010*).* Estimates efficacy based on a combination of empirical data and price elasticity estimates. *This technical manual was updated in 2021. Noting that tobacco has become more affordable in LMICs in recent years. Debunking concerns raised by industry that taxes will lower overall tax revenue, or drive inequalities. Effect estimate for absolute reduction given here is from this updated manual. | | Current prevalence - WHO's Global Health Observatory  Prevalence/incidence/mortality outcome data - GBD study  Current tax rates - Report on the Global Tobacco Epidemic (WHO, 2021)  Costs Estimates - Scaling up action against NCDs: How much will it cost? (WHO, 2011)  Relative Risk for Disease Outcomes - 3 existing reviews (1 x WHO report, 2 x peer-reviewed articles) of observational studies quantifying the independent association. (Diseases modelled against: IHD, Stroke, Cervical Cancer, Asthma, COPD, Type II Diabetes (RR 1.44), Lung Cancer. Effects modelled via direct reductions on outcome disease incidence, or through reductions in population risk) | |  | |
| Tobacco | Implement plain/standardized packaging and/or large graphic health warnings on all tobacco packages | | | | Assumed baseline prevalence of 25%, there is a 5% (credible range 2%-8%) relative reduction in prevalence, using a 5-year time horizon. Using a 40-year time horizon, the estimate is 10% relative prevalence reduction (credible range 5%-15%).  Unless updated on a regular basis with new content, the effectiveness of graphic warning labels may wane over time as consumers become too accustomed to their appearance. Messages can be reinforced by mass media campaigns. Evidence stronger for graphic health warnings than for plain packaging. | | | *Framework Convention on Tobacco Control (WHO, 2003)  Plain packaging of tobacco products: evidence, design and implementation (2016)* | | | 1,212-2,319 HLY gained per year per million people  1.25% absolute reduction in smoking prevalence | | *Levy et al. The Impact of Implementing Tobacco Control Policies: The 2017 Tobacco Control Policy Scorecard. (J Public Health Manag Pract, 2018).* Synthesises empirical evidence from HICs on tobacco policy from US taskforce reports, reviews, and primary articles | |  |  |  | |
| Tobacco | Enact and enforce comprehensive bans on tobacco advertising, promotion and sponsorship | | | | Assumed baseline prevalence of 25%, there is a 4% (credible range 2%-6%) relative reduction in prevalence, using a 5-year time horizon. Using a 40-year time horizon, the estimate is 6% relative prevalence reduction (credible range 3%-9%).  Include bans on direct advertising, such as TV, radio, magazine, newspaper, billboard, and retail point-of-sale advertising, and bans on indirect marketing, such as free distribution of products, promotional discounts, the appearance of tobacco products in TV or films, sponsorship of sports and music occasions, and the distribution of nontobacco products identified with tobacco brand names. Online advertising and indirect marketing efforts not included by the interventions which have been evaluated, and may offset these effects unless these channels are also targeted. Evidence that younger people and those with higher education benefit more. | | | Framework Convention on Tobacco Control (WHO, 2003) | | | 993-1,535 HLY gained per year per million people  0.75% absolute reduction in smoking prevalence | | Levy et al. The Impact of Implementing Tobacco Control Policies: The 2017 Tobacco Control Policy Scorecard. (J Public Health Manag Pract, 2018). Synthesises empirical evidence from HICs on tobacco policy from US taskforce reports, reviews, and primary articles | |  |  |  | |
| Tobacco | Eliminate exposure to second-hand tobacco smoke in all indoor workplaces, public places, public transport | | | | Assumed baseline prevalence of 25%, there is a 10% (credible range 5%-15%) relative reduction in prevalence, using a 5-year time horizon. Using a 40-year time horizon, the estimate is 12.5% relative prevalence reduction (credible range 7%-19%).  May have smaller effects if smoke-free policies are already prominent in private worksites; or if there is low compliance due to weak enforcement or a lack of antitobacco social norms. | | | *Framework Convention on Tobacco Control (WHO, 2003)* | | | 1,475-3,077 HLY gained per year per million people  2.7% absolute reduction in smoking prevalence | | Levy et al. The Impact of Implementing Tobacco Control Policies: The 2017 Tobacco Control Policy Scorecard. (J Public Health Manag Pract, 2018). Synthesises empirical evidence from HICs on tobacco policy from US taskforce reports, reviews, and primary articles | |  |  |  | |
| Tobacco | Implement effective mass media campaigns that educate the public about the harms of smoking/tobacco use and second hand smoke | | | | Assumed baseline prevalence of 25%, there is an 8% (credible range 4%-12%) relative reduction in prevalence, using a 5-year time horizon. Using a 40-year time horizon, the estimate is 10% relative prevalence reduction (credible range 6%-14%).  Effectiveness depends on whether the mass media campaign is well-tested, implemented on multiple media platforms, of sufficient scale, and sustained over time. The effectiveness of a media campaign may be enhanced if implemented alongside other interventions that increase the visibility and reach of the campaign. | | | *Framework Convention on Tobacco Control (WHO, 2003)* | | | 1,345-2,701 HLY gained per year per million people   2% absolute reduction in smoking prevalence | | Levy et al. The Impact of Implementing Tobacco Control Policies: The 2017 Tobacco Control Policy Scorecard. (J Public Health Manag Pract, 2018). Synthesises empirical evidence from HICs on tobacco policy from US taskforce reports, reviews, and primary articles | |  |  | A core component of these interventions is health education, which does not meet the definition of a population-level intervention. | |
| Alcohol | Increase excise taxes on alcoholic beverages | | | | A 50% increase in excise taxes over current tax rates (country-specific) was modelled. Beverage-specific demand elasticities for alcohol, by country income level, were used, accounting for estimated untaxed alcohol consumed.  Levying taxes should be combined with other price measures, such as bans on discounts or promotions; and work to reduce illegal alcohol consumption which bypasses taxation. Detailed discussion of mechanism of excise taxation - i.e. taxation rate based on strength, quantity, or cost - in the 2017 resource, with conclusion that each has their strengths and weaknesses but the overall effect will consistently be reduced alcohol consumption (but differences in the effect on overall tax revenue and rate of substitutions). | | | SAFER - A world free from alcohol related harms. The Technical Package (WHO, 2019)  Resource tool on alcohol taxation and pricing policies (WHO, 2017) | | | 155-797 HLY gained per year per million people  Price elasticity -0.51 to -0.77 (meaning a 10% increase in price results in an average 5.1% - 7.7% reduction in consumption) | | *Chisholm D, Moro D, Bertram M, et al. (2018) Are the "Best Buys" for alcohol control still valid? An update on the comparative cost-effectiveness of alcohol control strategies at the global level. J Stud Alcohol Drugs 79(4): 514-522.* N.B. peer-reviewed, but members of this author group from the WHO team.   *Resource tool on alcohol taxation and pricing policies (WHO, 2017).* These documents draw data from reviews of empirical data from across HIC and LMICs. | | Rates of at-risk alcohol consumption considered as hazardous and harmful - WHO Global Information System on Alcohol and Health. (Defined as more than an average of 40g of pure alcohol per day for males, and 20g for females)  Sex-specific relative risks for diseases and injuries - Global status report on alcohol and health 2018 (WHO, 2018), and GBD study. (Outcomes modelled against: alcohol use disorders, epilepsy (100%), TB, pancreatitis, cirrhosis, various cancers, stroke, hypertension (YLDs Male 13%, Female 5%), various injuries including road traffic injuries, falls, and self-harm (approx. 14%, 4%))  Model - One Health tool. WHO-CHOICE health economic model which estimates the population-level health effects over 100 years, against a counterfactual of no intervention. | |  | |
| Alcohol | Enact and enforce bans or comprehensive restrictions on exposure to alcohol advertising (across multiple types of media). | | | | Source study find a 3% reduction in drinking volume per additional unit (none, voluntary or self-regulation, partial statutory restriction, ban) of marketing restriction for beer, wine and spirits combined across 4 types of media (national TV, national radio, print media, and billboards). WHO team have then simulated this effect size to all world regions, accounting for their existing level of policy in place.   Effect estimate doesn't include bans on web advertising, sponsorship; or the differentiation between domestic and cross-border marketing. SAFER package notes that: restricting only one aspect of the marketing mix often results in an expansion of activity in other parts of the mix. In general, the more complete the regulation on marketing activities, the easier it will be to implement the regulation and the more effective it will be in reducing alcohol-related harm. That is why a comprehensive ban or set of restrictions is preferred. Such frameworks should ideally incorporate all forms of new and emerging media as well as existing media and other promotional channels. This is explored in more detail in the 2022 report on cross-border marketing. | | | SAFER - A world free from alcohol related harms. The Technical Package (WHO, 2019)  Reducing the harm from alcohol by regulating cross-border alcohol marketing, advertising and promotion. A technical report (WHO, 2022) | | | 121-158 HLY gained per year per million people  1.2% absolute reduction in prevalence of hazardous/harmful drinking | | *Cook WK, Bond J, Greenfield TK (2014) Are alcohol policies associated with alcohol consumption in Low and Middle income countries? Addiction 109(7): 1081-1090.* Cross-sectional, regression analyses of data on alcohol policies and alcohol consumption from 15 LMICs, adjusted for age, gender, and other policies, which found an inverse association between increased marketing restrictions and total drinking volume.  This is supported by: *Rossow I (2021). The alcohol advertising ban in Norway: effects on recorded alcohol sales. Drug Alcohol Rev.* Which used a time series analysis, and alcohol sales data from Norway, 1960-2006, to assess the long-term effects of the 1975 complete advertising ban, adjusted for alcohol price changes and wages. They found a 7.4% reduction in alcohol sales. | |  |  | The WHO 2022 report 'Reducing the harm from alcohol by regulating cross-border alcohol marketing, advertising and promotion. A technical report' explores the need for supra-national co-operation to effectively tackle the global advertising landscape, and the legislative considerations for this.   It cites several reviews and editorials, together presenting comprehensive evidence that alcohol industry self-regulation is not an effective tool for reducing alcohol-related harm, and these endeavours should be led by governments.   It also explores newer options which need to be evaluated, such as removing tax exemptions for alcohol advertising spending, and blocking any collection of consumer data for the purposes of alcohol marketing. | |
|  | Enact and enforce restrictions on the physical availability of retailed alcohol (via reduced hours of sale) | | | | Source study reports a regression coefficient for restricted hours of sale of -0.88 (95% CI -1.44, 0.32). WHO team have them simulated this effect size to all world regions, assuming a gamma distribution, to assess for the reduction in hazardous and harmful drinking that would be achieved.  Alongside restricting hours of sale, other mechanisms described in the SAFER package include: regulate the number, density and location of retail alcohol outlets; establish a national legal minimum age for purchase and consumption of alcohol; and restrict the use of alcohol in public places. | | | SAFER - A world free from alcohol related harms. The Technical Package (WHO, 2019) | | | 132-190 HLY gained per year per million people  1.8-2.1% (male), 4% (female) reduction in prevalence of hazardous/harmful drinking | | *Cook WK, Bond J, Greenfield TK (2014) Are alcohol policies associated with alcohol consumption in Low and Middle income countries? Addiction 109(7): 1081-1090.* Cross-sectional, regression analyses of data on alcohol policies and alcohol consumption from 15 LMICs, adjusted for age, gender, and other policies, which found an inverse association between increased restrictions on business hours for off-premises alcohol sales and total drinking volume | |  |  | Note, statistically significant effects are also reported in this study for reducing density of outlets and introducing minimum legal drinking age, but these are not included as Best Buys - likely due to lack of other required info for WHO-CHOICE analysis (costs of implementation, current policies around the world etc.) | |
| Alcohol | Establish minimum prices for alcohol where applicable | | | | Beverage-specific effects of a 10% increase in minimum price in British Columbia, Canada were: spirits and liqueurs by 6.8% (P = 0.004), wine by 8.9% (P = 0.033), alcoholic sodas and ciders by 13.9% (P = 0.067), beer by 1.5% (P = 0.043). But these include some degree of substitution. Simultaneous 10% increase in the minimum prices of all types reduced total alcohol sales by 3.4%.   In Scotland, a reduction of 3.5% was noted in the 1st year after implementation, compared to England. This was in response to an approximate 7.9% increase in the average cost per household following the introduction of the MUP (O'Donnell, BMJ, 2019). | | | SAFER - A world free from alcohol related harms. The Technical Package (WHO, 2019)  Resource tool on alcohol taxation and pricing policies (WHO, 2017) | | | 10% increase in minimum price reduces total alcohol sales by 3.4% | | References from the 2020 WHO report 'Alcohol pricing in the WHO European Region' *Stockwell T, Auld MC, Zhao J, Martin G. Does minimum pricing reduce alcohol consumption? The experience of a Canadian province. Addiction. 2012 May;107(5):912-20.* Natural experiment study using time series analysis to estimate the impact of changes to the minimum unit price over a 20 year period in British Columbia, Canada, on quarterly alcohol sales, with adjustment for household income and alcohol price.   *Robinson, M., Mackay, D., Giles, L., Lewsey, J., Richardson, E., and Beeston, C. (2021) Evaluating the impact of minimum unit pricing (MUP) on off-trade alcohol sales in Scotland: an interrupted time–series study. Addiction, 116: 2697– 2707.* Interrupted time series analysis estimating the effect of the 2018 MUP in Scotland on off-trade alcohol sales, compared to England (no MUP). | | [British Columbia analysis] Analysis of effectiveness is supported by 3 other similar analyses from British Columbia, which observe reductions in alcohol-related hospital admissions, deaths, and traffic offences.   [Scotland analysis] Supported by a similar analysis from a different set of academics (O'Donnell, BMJ, 2019) which reach similar conclusions using consumer panel data. A further paper, not referenced in WHO documents as it was only published in March 2023 (Wyper, Lancet, 2023) found an associated reduction in alcohol-attributable hospital mortality.  [Both] These analyses are further supported by several, cited, international, modelling studies which conclude in support of the potential of MUP policies. | | SAFER package frames MUP as an adjunct to excise taxation, which may have the effect of causing retailers to lower the cost of their products and absorb the increased taxation (or in the case of ad valorem taxes, avoid them). | |
| Alcohol | Restrict or ban promotions of alcoholic beverages in connection with sponsorships and activities targeting young people | | | | n/a | | | SAFER - A world free from alcohol related harms. The Technical Package (WHO, 2019)  Reducing the harm from alcohol by regulating cross-border alcohol marketing, advertising and promotion. A technical report (WHO, 2022) | | | No effect size reported | | *Brown K, Association Between Alcohol Sports Sponsorship and Consumption: A Systematic Review, Alcohol and Alcoholism, Volume 51, Issue 6, 26 November 2016, Pages 747–755.* Reports evidence from 7 studies from HICs that show an association between exposure to sports club sponsorship by alcohol companies, and higher self-reported alcohol consumption - by children and adults. | | Further, there is a large body of literature cited in the 2022 WHO report on cross-border marketing which demonstrates a relationship between advertising, including digital advertising, to young people, and alcohol-related preferences and behavioural intentions, and to the increased susceptibility of children to advertising (of alcohol and other products). | | The WHO 2022 report 'Reducing the harm from alcohol by regulating cross-border alcohol marketing, advertising and promotion. A technical report' cites several reviews and editorials, together presenting comprehensive evidence that alcohol industry self-regulation is not an effective tool for reducing alcohol-related harm, and these endeavours should be led by governments.  Note that e-sports are increasingly popular with younger people, and are not yet legally recognised as 'sports' in many countries, presenting a potential loop hole for industry sponsorship.   Also highlights the importance of supra-national co-operation, given the cross-national nature of modern alcohol marketing. | |
| Alcohol | Provide consumer information about, and label, alcoholic beverages to indicate, the harm related to alcohol | | | | n/a | | | Reducing the harm from alcohol by regulating cross-border alcohol marketing, advertising and promotion. A technical report (WHO, 2022) | | | Mixed evidence, suggestive of a null or very minimal effect | | Four cited reviews (Stockwell 2006, Babor 2010, O'Brien 2014, Kokole 2021) either that the evidence points to meaningful increases in public awareness and intentions, but that the resultant effect on drinking behaviour is minimal to non-existent; or that there is a paucity of evidence. Some newer evidence does suggest reductions in alcohol consumption, and frames this in the context of better understanding of what works on labelling: *Zhou J, Stockwell T, Vallance K, Hobin E (2020). The effects of alcohol warning labels on population alcohol consumption: an interrupted time series analysis of alcohol sales in Yukon, Canada. J Stud Alcohol Drugs. 81(2): 225–37.* An interrupted time series analysis of the effect of introducing labels pertaining to cancer risk, drinking guidelines, harms for pregnant women, and not to operate machinery, on alcohol beverages in 2017 in Yukon, Canada, using neighbouring areas as a control - reporting a 6.31% reduction in alcohol sales. | |  | | It is noted that such messages could change the social acceptability of alcohol, and therefore of future (more effective) alcohol-related public policies such as taxation. | |
| Alcohol | Enact and enforce an appropriate minimum age for purchase or consumption of alcoholic beverages and reduce density of retail outlets | | | | Report a regression coefficient of −0.26 (95% CI −0.48,−0.03) for relative change in total alcohol consumption, but not possible to convert this to a % reduction in hazardous/harmful alcohol consumption from the data provided. However, coefficient for reduced hours of sale from same analysis was -0.88, and WHO modelled this to equate to a 2-4% reduction in hazardous/harmful drinking, so it is likely this effect size would be quite small. Only 2 of the 15 countries included in the analysis did not have minimum legal drinking ages in place  SAFER package: "“Mystery shoppers” – in this case, under-age purchasers – can be used to ensure that establishments enforce the minimum age for purchase. In accordance with national legislation, mechanisms should be considered for placing liability on sellers and servers to prevent sales to intoxicated persons and those below the legal drinking age. Secondary supply of alcohol – e.g. by parents or friends – should be taken into consideration in measures to limit the availability of alcohol – perhaps in the form of fines and/or criminal charges, depending on the circumstances. Alcohol consumption by minors in the presence of parents, family or friends in on-trade establishments undermines" | | | SAFER - A world free from alcohol related harms. The Technical Package (WHO, 2019)  Reducing the harm from alcohol by regulating cross-border alcohol marketing, advertising and promotion. A technical report (WHO, 2022) | | | No effect size reported | | *Cook WK, Bond J, Greenfield TK (2014) Are alcohol policies associated with alcohol consumption in Low and Middle income countries? Addiction 109(7): 1081-1090.* Cross-sectional, regression analyses of data on alcohol policies and alcohol consumption from 15 LMICs, adjusted for age, gender, and other policies, which found an inverse association between minimum legal drinking age and total drinking volume.  A textbook cited by the 2022 report on reducing cross-border alcohol marketing (Babor et al.) in turn cites: Wagenaar A, Toomy T. *Effects of minimum drinking age laws: review and analyses of the literature from 1960 to 2000. Journal of Studies on Alcohol, Supplement, (s14), 206–225 (2002).* A systematic review which finds consistent evidence a reduction in road traffic collisions, and some evidence of a reduction in alcohol consumption, following the introductions of minimum age restrictions for purchasing alcohol. | |  | |  | |
| Diet/Obesity | Reduce sugar consumption through effective taxation on sugar-sweetened beverages | | | | Assumed that taxation led to a 20% increase in retail price. Assumed a price elasticity of -0.8.   Included: carbonated soft drinks, non-carbonated soft drinks, fruit and vegetable juices, liquid powder concentrates, flavoured water, energy and sports drinks, ready-to-drink tea and coffee, flavoured milk drinks. Excluded: low calorie / low sugar product categories as these contained a variable mix of products with or without added sugars, and/or artificial sweeteners. Assumed no substitution to untaxed beverages (based on systematic review by Andreyeva, 2022). SSB consumption levels were assumed to be the same across all population groups within a given country. SSB consumption volume was converted into the number of daily servings per capita, assuming a serving size of 355 ml. | | | WHO manual on sugar-sweetened beverage taxation policies to promote healthy diets (WHO, 2022)  Policy brief on fiscal policies to promote healthy diets (WHO, 2022)  SSB taxes in the WHO European region: success through lessons learned and challenges faced (WHO, 2022) | | | 7-41 HLY gained per year per million people   Price elasticity -0.8 to -1.59 (meaning a 10% increase in price results in an average 8% - 15.9% reduction in consumption) | | Considered evidence from 16 reviews, including the World Bank's 2020 review: '*TAXES ON SUGAR-SWEETENED BEVERAGES: International Evidence and Experiences'* and several systematic reviews (most recent '*Andreyeva T, Marple K, Marinello S, Moore TE, Powell LM (2022) Outcomes Following Taxation of Sugar-Sweetened Beverages: A Systematic Review and Meta-analysis. JAMA Network Open 5(6): e2215276*'). Range of price elasticities -0.8 to -1.59. Selected the lowest estimate here for a conservative analysis.   Further supportive evidence is provided by the most up to date summaries of empirical evidence, from the 2022 WHO 'Policy brief on fiscal policies to promote healthy diets' and the 2022 'WHO manual on sugar sweetened beverage taxation policies to promote healthy diets', which report a price elasticity of -0.8 to -1.3, and also demonstrates healthy equity effects of the taxation, with the greatest reductions in purchasing found in lower SES groups. | | Consumption of sugar-sweetened beverage - Euromonitor. https://www.euromonitor.com/our-story  Baseline BMI - NCD Risk Factor Collaboration (NCD-RisC) (2017) Worldwide trends in body-mass index, underweight, overweight, and obesity from 1975 to 2016: a pooled analysis of 2416 population-based measurement studies in 128·9 million children, adolescents, and adults. Lancet 390(10113): 2627-2642. Population-distribution of BMI assumed to follow log-normal distribution.  All-cause mortality from hazard ratios for each BMI group: Global BMI Mortality Collaboration, Di Angelantonio E, Bhupathiraju ShN, et al. (2016) Body-mass index and all-cause mortality: individual-participant-data meta-analysis of 239 prospective studies in four continents. Lancet 388(10046): 776-786.  Country estimates for population level intakes: GBD study and Global Dietary Database for adults and children, respectively.  Model - OneHealth tool. WHO-CHOICE health economic model which estimates the population-level health effects over 100 years, against a counterfactual of no intervention. Disease outcomes modelled are not as clearly explained as for other risk factors, but appear to be based primarily on lowering of cholesterol, with conversions of reductions in energy and fat to cholesterol derived from systematic reviews. | |  | |
| Diet/Obesity | Policies to protect children from the harmful impact of food marketing | | | | The evidence for policies to reduce the power of and children’s exposure to food and non-alcoholic beverage marketing is scarce; however, there is evidence confirming that such marketing practices are abundant and that they are for unhealthy food high in fats, sugars and salt; and evidence on the impact of marketing on children is unequivocal and has recently been updated in a new systematic review. Therefore, the assumption is made that by adopting and implementing such policies, a reverse impact of equivalent magnitude is likely.  The 2022 policy brief states the importance of comprehensive (all forms of media), and mandated marketing restrictions, over partial and voluntary approaches which were historically employed, and are less likely to be effective (and industry are likely to lobby for). As for alcohol marketing, important to recognise the cross-national nature of digital media. Also importance of protecting children up to the age of 18, rather than just under 12s. | | | Protecting children from the harmful impact of food marketing: policy brief (WHO, 2022)  Framework for implementing the set of recommendations on the marketing of foods and non-alcoholic beverages to children (WHO, 2012)  Tackling food marketing to children in a digital world: trans-disciplinary perspectives (WHO, 2016)  Regional action framework on protecting children from the harmful impact of food marketing in WPRO (WHO, 2020)  A Child Rights-Based Approach to Food Marketing: A Guide for Policy Makers (WHO/UNICEF, 2018) | | | 297-610 HLY gained per year per million people  -60 kcal/day/person | | *Russell, SJ, Croker, H, Viner, RM (2019) The effect of screen advertising on children's dietary intake: A systematic review and meta-analysis. Obes. Rev. 20: 554-568.* An SR an MA of 16 studies which compared the eating behaviours of children exposed to unhealthy food TV advertising (n=11) or advergames (n=5) to a control group. Most studies were conducted either in a lab or school setting, with only immediate follow-up assessment. Conversion to weight loss from a modelling study. Mix of evidence from HICs and LMICs | |  |  |  | |
| Diet/Obesity | Front-of-pack labelling as part of comprehensive nutrition labelling policies for facilitating consumers’ understanding and choice of food for healthy diets | | | | Reduction in % of energy from trans-fatty acids attributed to mandatory declaration of nutrients leading to product reformulation. Reductions in intake of energy, and SFA, are from consumer behaviour changes in response to nutritional labels, and represents only short-term effects. The WHO team selected the most conservative estimates, from real-world analyses (rather than lab-based experiments) for each outcome.   The Codex Alimentarius Commission (Codex) identifies three types of nutrition labelling: nutrient declarations; nutrition and health claims; and supplementary nutrition information, which includes front-of-pack labelling (FOPL).  Guiding principles and framework document describes FOPL as: (i) presented on the front of food packages (in the principal field of vision) and can be applied across the packaged retail food supply; (2) comprise an underpinning nutrient profile model that considers the overall nutrition quality of the product or the nutrients of concern for NCDs (or both); and (3) present simple, often graphic information on the nutrient content or nutritional quality of products, to complement the more detailed nutrient declarations usually provided on the back of food packages. States FOPL mandates should exclude baby and infant food (because they are already held to specific standards), condiments/tea/coffee (because they have low nutritional effects), and alcohol (no reason given). | | | Nutrition labelling: policy brief (WHO, 2022)  Guiding principles and framework manual for front-of-pack labelling for promoting healthy diet (2019) | | | 4,042-9,069 HLY gained per year per million people  92.5% reduction in % of energy from trans-fatty acids   5.3% reduction in energy intake  6.8% reduction in the % of energy intake from saturated fatty acids  6.4% reduction in sodium intake | | *Ratnayake WN, Swist E, Zoka R, Gagnon C, Lillycrop W, Pantazapoulos P (2014) Mandatory trans-fat labelling regulations and nationwide product reformulations to reduce trans fatty acid content in foods contributed to lowered concentrations of trans fat in Canadian women's breast milk samples collected in 2009-2011. Am. J. Clin. Nutr. 100(4):1036-40. Change in average TFA content of breast milk from a Canadian cohort in 1992 to a separate cohort in 2009-11 (Intervention implemented in early 2000s).* Looking at the primary studies, no information available on the cohort profile or sampling methods for the 1992 cohort, though they did represent provinces across Canada. Cohort profile for later cohort shows clear healthy volunteer bias, with cohort significantly better educated than average Canadians, response rate of 39% (range across recruitment sites 13%-90%). Also unclear validity of making this comparison, without accounting for other interventions which could have contributed to the reduction. *Song J, Brown MK, Tan M, et al. (2021) Impact of color-coded and warning nutrition labelling schemes: A systematic review and network meta-analysis. PLOS Med 18(10): e1003765.* SR and NMA of 101 RCTs and 55 quasi-experimental studies from Europe, Northern America, and Latin America, studying effects of nutritional labelling on consumer purchasing behaviours. Reported effects for reduction in energy intake and % of energy from SFA. | |  |  | N.B. a 2023 study assessing the effect of the US Healthy, Hunger-Free Kids Act of 2010 (HHFKA) through an ITS design demonstrated an empirical associated reduction in BMI following the intervention (Chandran, JAMA Paediatrics, 2023) | |
| Diet/Obesity | Reformulation policies for healthier food and beverage products (e.g. elimination of trans-fatty acids and/or reduction of saturated fats, free sugars and/or sodium) | | | | [TFA] Based on data from Denmark after a mandated reduction of artificial TFA in the food chain to <2g/100g of oil or fat.   [Sugar] Modelled an 11% reduction in dietary sugar.  [Sodium] Modelled a 0.57g/day reduction in dietary sodium.   Reformulation may result from policies setting mandatory limits or voluntary targets for nutrient content in food and beverage products, or it may happen in the absence of a specific reformulation policy, as result of industry response to e.g. a FOPL or food or beverage tax policy. Evidence from the contributing systematic reviews suggest that mandatory limits have been more effective. | | | REPLACE trans-fat: an action package to eliminate industrially produced trans-fatty acids (WHO, 2021)  WHO global sodium benchmarks for different food categories (WHO, 2021)  SHAKE the salt habit: technical package for salt reduction (WHO, 2016) | | | 2,449-4,704 HLY gained per year per million people  100% reduction (elimination) of TFA  2-11% reduction in dietary sugar per person per day (resulting in 1.04kg reduction in body weight)  5-10% (-0.57g) reduction per person per day in dietary sodium, resulting in approximately -0.53mmHg of SBP | | *[TFA] Restrepo BJ Rieger M (2016) Denmark's policy on artificial trans-fat and cardiovascular disease. Am J Prev Med 50(1):69-76.* Estimate a resultant reduction in CVD-attributable mortality of 14/100,000 people per year, supported by similar findings from another study from New York banning TFA use in restaurants (13/100,000).  *[SUGAR] Hashem KM, He FJ, MacGregor GA (2019) Effects of product reformulation on sugar intake and health-a systematic review and meta-analysis. Nutr Rev. 77(3):181-196.*Two analyses reported. (i) Feasible reductions in sugar consumption in the population achieved by reformulation policies: averaged across one observational study and several modelling studies. Average 91g/day (range in modelling studies 0.2-62.1g/day; observational study 0.25g/day (2%). (ii) Meta-analysis of 3 small RCTs of low sugar replacements, with follow up ranging from 8 to 10 weeks, observed a reduction in dietary sugar of 11%, with a corresponding estimate of 1.04kg reduction in body weight. Data from HICs only   [Sodium] *Gressier M, Swinburn B, Frost G, Segal AB, Sassi F (2021) What is the impact of food reformulation on individuals' behaviour, nutrient intakes and health status? A systematic review of empirical evidence. Obes Rev. 22(2): e13139.* Review focused on consumer behaviours in response to product reformulations, finding that in the majority of studies consumers continued purchasing the reduced sodium product, and meta-analysis found an overall positive effect on reductions in sodium consumption. Follow-up 4-27 years. Resulting dose-response effect on blood pressure derived from an SR and MA. Most, but not all, data from HICs | |  |  |  | |
| Diet/Obesity | Public food procurement and service policies for healthy diets (e.g. to reduce the intake of free sugars, sodium, unhealthy fats, and to increase the consumption of legumes, wholegrains, fruits and vegetables) | | | | SSB reduction resulted from competitive standards. Fruit increase, sodium decrease, and SFA decrease resulted from school nutrition standards. A smaller effect of +0.28 (combined fruit and vegetables) was seen for direct provision, but the higher value of 0.76 is modelled here, as this is thought to be a more common intervention in LMICs. Effect of SSB modelled on BMI; fruit on CHD, CVD, stroke, cancer, all-cause mortality; sodium on BP; and SFA on cholesterol. With conversions derived from systematic reviews and meta-analyses.  2021 action framework suggests that public food procurement could influence private sector employers to follow suit. It also points to evidence from Brazil where such policies have had co-benefits of guaranteed income for local farmers, and reducing climate footprint. | | | Action framework for developing and implementing public food procurement and service policies for a healthy diet | | | 441-449 HLY gained per year per million people  -0.18 servings per day of SSB (serving=355mls)  +0.76 servings per day of fruit (serving=80g)   -0.17g per day of sodium  -0.93% energy/day from SFA | | *Micha R, Karageorgou D, Bakogianni I, et al. (2018) Effectiveness of school food environment policies on children's dietary behaviours: A systematic review and meta-analysis. PLoS One 13(3): e0194555.* SR and MA of 91 empirical studies, from a range of HICs. Considered 3 types of school food environment change: direct provision of healthier foods, setting healthier school nutrition standards (i.e. all provided school meals must include x portions of vegetables, <y% saturated fat etc.), and affecting the standards of 'competitive' foods (i.e. foods available for purchase at school).   *Driessen, C.E., Cameron, A.J., Thornton, L.E., Lai, S.K. and Barnett, L.M. (2014), Effect of changing school food environment. Obes Rev, 15: 968-982.* SR of 18 studies of changes to the school food environment, e.g. nutritional policies affecting canteens, vending machines, tuck shops. 17/18 studies reported positive effects on diet, and/or BMI. However, study quality mixed.   *Niebylski, M.L.; Lu, T.; Campbell, N.R.C.; Arcand, J.; Schermel, A.; Hua, D.; Yeates, K.E.; Tobe, S.W.; Twohig, P.A.; L'Abbé, M.R.; Liu, P.P. Healthy Food Procurement Policies and Their Impact. Int. J. Environ. Res. Public Health 2014, 11, 2608-2627.* SR of 34 studies (schools n=19, worksites n=6, institutional settings (e.g. hospitals, prisons) n=6, remote communities n=3 of introduction of healthy food procurement policies on diet and/or BMI. Almost all showed positive effects, with a wide range of effect sizes. Almost all data from HICs. Study quality variable, and unable to assess changes to diet outside of settings. | |  |  |  | |
| Diet/Obesity | Subsidies on healthy foods and beverages (e.g. fruits and vegetables) as part of comprehensive fiscal policies for healthy diets | | | | The reviews pooled evidence from interventions to change the cost to the consumer of healthy foods across a range of settings (e.g. supermarkets, vending machines, cafeterias), but generally excluded agricultural subsidies. Interventions included reducing the cost of purchase, and provision of specific vouchers. Most studies assessed the effect on fruit and vegetables, and effect sizes were also consistent for other healthy options such as 'low fat' food products. One of the reviews found a null effect on non-alcohol healthy beverages, but this may have been underpowered. | | | Policy brief on fiscal policies to promote healthy diets (WHO, 2022)  Fiscal Policies for Diet and Prevention of Noncommunicable Diseases (WHO, 2016) | | | 10% increase in healthy foods and beverages, such as fruit and vegetables (increases sales of those items by 5.9% - 12.0%) | | *Andreyeva T, Marple K, Moore TE, Powell LM. Evaluation of Economic and Health Outcomes Associated With Food Taxes and Subsidies: A Systematic Review and Meta-analysis. JAMA Netw Open. 2022;5(6):e2214371.*Systematic review (n=54 studies) and meta-analysis (n=13) studies of the effect of healthy food subsidies on sales and consumption. Majority of studies examining sales were rated as high-quality, price elasticity estimate -0.59 (95% CI -1.04, -0.13). Those measuring consumption directly were generally of lower quality, estimate -0.17 (95% CI, -0.49, 0.15 [P = .26].  *Afshin A, Peñalvo JL, Del Gobbo L, Silva J, Michaelson M, et al. (2017) The prospective impact of food pricing on improving dietary consumption: A systematic review and meta-analysis. PLOS ONE 12(3): e0172277.* Systematic review (n=30 studies) and meta-analysis (n=12) estimating the price elasticity as -1.2 (95% CI -1.5, -1.0) for healthy foods (inclusive of fruit and vegetables, which had a similar effect size of -1.4 (-1.7, -1.1) in separate meta-analysis). | | *Milani C, Lorini C, Baldasseroni A, Dellisanti C, Bonaccorsi G. An Umbrella Review and Narrative Synthesis of the Effectiveness of Interventions Aimed at Decreasing Food Prices to Increase Food Quality. Int J Environ Res Public Health. 2019 Jul 2;16(13):2346.* This umbrella review with narrative synthesis, alongside several modelling studies have led the WHO 'policy brief on fiscal policies to promote healthy diets' to suggest that emerging evidence supports a combination of SSB taxation with healthy food subsidy would achieve the most effective and equitable results. | |  | |
| Diet/Obesity | Menu labelling in food service to promote healthy diets (e.g. reduce total energy intake (kcal) and/or intake of sugars, sodium and unhealthy fats) | | | | n/a | | | n/a | | | n/a | | No evidence is presented in any of the reviewed WHO documents in regard of this recommendation. The WHO are currently preparing a scoping review and guideline on this topic. | |  | |  | |
| Diet/Obesity | Limiting portion and package size to reduce energy intake and the risk of overweight/obesity | | | | n/a | | | n/a | | | n/a | | No evidence is presented in any of the reviewed WHO documents in regard of this recommendation. The WHO are currently preparing a scoping review and guideline on this topic. | |  | |  | |
| Physical Inactivity | Implement urban and transport planning and urban design, at all levels of government, to provide compact neighbourhoods providing mixed-land use and connected networks for walking and cycling and equitable access to safe, quality public open spaces that enable and promote physical activity and active mobility. Improve walking and cycling infrastructure ensuring universal and equitable access to enable and promote safe walking, cycling, other forms of micro mobility (e.g. wheelchairs, scooters and skates) by people of all ages and abilities. | | | | In response to introduction of a congestion charge, and/or improving cycling infrastructure. Greater detail about the types of urban changes which are thought to be effective available in the implementation guides. | | | ACTIVE: a technical package for increasing physical activity (WHO, 2018)  Towards More Physical Activity in Cities (WHO Europe, 2018) | | | 20% increase in cycling journeys | | The WHO Europe 2006 report 'Physical activity and health in Europe: evidence for action' presents two cases studies: the 2003 introduction of a congestion charge in London, England, and the 1999-2002 major investment in cycling infrastructure (alongside mass media campaign) in Odense, Denmark. Both are reported to have been associated with a 20% increase in cycling journeys. No references to peer-reviewed literature with detailed methodology are available. | | A study (*Sallis et al. 2016 Lancet*) referenced by the 2018 global action plan performed cross-sectional analysis to compare differences in physical activity levels across 14 cities in 10 countries (on 5 continents) associated with indicators of walkability, public transport access, and park access. They found that those living in the top 5% of activity-friendly areas engaged in an extra 21-32 mins/week of MVPA.   A systematic review (*Ogilvie, BMJ, 2004*) referenced by the 2006 Evidence for Action report identified a controlled study in the Netherlands which found that extensions to the cycle route networks increased the share of trips made by bike by 3% after 3 years. Though other, uncontrolled, studies identified by the review did not report positive findings.   The 2018 report '*Towards More Physical Activity in Cities'* and the 2006 report *'Promoting physical activity and active living in urban environments. The role of local governments'* by WHO Europe summarise the supporting evidence base of observational and qualitative data demonstrating associations between the urban environment design and likelihood of partaking in physical activity. This includes a case study focus on progressive urban planning policies in Copenhagen, Denmark over several decades, noting they have amongst the highest modal split of active travel journeys in the world. | | N.B. we have combined two recommendations into one here as they both pertain to policies/interventions to improve access to physical activity and active travel in the built environment | |
| Physical Inactivity | Implement whole-of-school programmes that include quality physical education, and adequate facilities, equipment and programs supporting active travel to/from school and support physical activity for all children of all abilities during and after school | | | | This effect size is for an intervention that primarily acted by increasing the quality and quantity of PE lessons. However, it is within the range indicated by reviews of broader intervention types, including provision of physical activity equipment, establishment of physically active after school clubs, and built environment changes to support active travel. | | | Promoting physical activity through schools: a toolkit (WHO, 2021)  ACTIVE: a technical package for increasing physical activity (WHO, 2018) | | | 11 additional minutes of MVPA per day | | *van Sluijs E M F, McMinn A M, Griffin S J. Effectiveness of interventions to promote physical activity in children and adolescents: systematic review of controlled trials BMJ 2007; 335 :703.* SR of 57 studies, of which 15 included a population-level component (e.g. improvements to the playground, provision of play equipment, increasing PA opportunities within the school day and at after school clubs, construction of walking/cycle paths in the surrounding community). Generally reasonable-quality evidence, only included controlled trials. Interventions ranged from a few weeks to a year. Based on the level and consistency of evidence, the authors concluded there was limited evidence of a benefit to physical activity levels, with a large range of effect sizes reported (3-83 extra mins/week) - noting that environmental interventions were more likely to be effective than educational interventions alone.  The 2021 toolkit picks out two individual trials: *Kriemler S, Zahner L, Schindler C, Meyer U, Hartmann T, Hebestreit H et al. Effect of school based physical activity programme (KISS) on fitness and adiposity in primary schoolchildren: cluster randomised controlled trial BMJ 2010; 340 :c785.* A cluster RCT testing a 9-month multicomponent school-based intervention including additional PE lessons, daily short activity breaks, as well as PE-based homework (e.g. balance exercises whilst brushing teeth). PA measured via accelerometer showed increase in in-school PA and MVPA, and total MVPA (11 additional mins/day). However, out of school MVPA and total PA were non-significant. Other outcomes included adiposity, fitness, and quality of life, which all showed small, statistically significant benefits (e.g. the intervention groups' adiposity increased by 6% less than the control groups').   *Chesham, R.A., Booth, J.N., Sweeney, E.L. et al. The Daily Mile makes primary school children more active, less sedentary and improves their fitness and body composition: a quasi-experimental pilot study. BMC Med 16, 64 (2018).* This quasi-experimental study at the school-level was at higher risk of bias. It investigated the effect of adding the daily mile to the school day. After 7 months, found an increase in MVPA, measured by accelerometer, of 9.1 min/day (95% CI 5.1-13.2min). However, intervention and control schools were assessed at different times of the year (baseline for intervention schools in winter, whilst control schools in spring) and only 32-67% of students had valid accelerometer data. | | *Owen MB, Curry WB, Kerner C, Newson L, Fairclough SJ. The effectiveness of school-based physical activity interventions for adolescent girls: A systematic review and meta-analysis. Preventive medicine. 2017 Dec 1;105:237-49*. This more recent SR of 17 studies focuses only on adolescent girls and finds similar results to van Slujis: evidence of variable quality with several studies using short follow up. Meta-analysis showed a very small effect favouring intervention, not reaching statistical significance.   *Martin, Murtagh E (2017). Effect of Active Lessons on Physical Activity, Academic, and Health Outcomes: A Systematic Review, Research Quarterly for Exercise and Sport, 88:2, 149-168.*SR of 10 studies of active classroom interventions on PA and BMI. Evidence generally at high-risk of bias, e.g. short follow up, self-reported outcome data. Found a large range of effect sizes, with a very small effect on BMI. | | The 2021 toolkit identifies 6 parts to a whole-of-school physical activity programme: quality PE, interventions to increase active travel, PA opportunities before and after school, PA opportunities in lunch/recess, active classrooms, PA for those with additional needs. These variably represent predominantly population-level interventions. | |
| Physical Inactivity | | | Implement multi-component workplace physical activity programmes | | A 7% increase in active commuting was reported 6 months after installation of shower facilities, and lobbying local govt to improve cycling infrastructure. | | | ACTIVE: a technical package for increasing physical activity (WHO, 2018) | | | 7% increase in active commuting | | A systematic review referenced by the global action plan (Foster et al. 2004 J of Sports Sciences) found one before-after study. | | A systematic review (Kahn et al. 2002, Am J Prev Med) referenced by the 2006 Evidence for Action report, identified 10 studies of environmental changes to increase PA, such as provision of gym equipment or walking trails. Study quality was variable. All studies reported an increase in PA, with a range of outcome measures and effect sizes. | | 2 systematic reviews (Dishman et al., 1998, Am J Prev Med) (Proper et al., 203, Clin J of Sport Med) of workplace-based physical activity interventions referenced by the 2006 Evidence for Action report identified only individual-level interventions (predominantly health education or structured exercise programmes). | |
| Physical Inactivity | | | Provide and promote physical activity through provision of community-based (grass roots) sport and recreation programmes and conduct free mass participation events to encourage engagement by people of all ages and abilities | | n/a | | | ACTIVE: a technical package for increasing physical activity (WHO, 2018) | | | No effect size reported | | No direct, empirical evidence is presented in any of the reviewed WHO documents in regard of this recommendation. | | The WHO Europe 2006 report 'Promoting physical activity and active living in urban environments. The role of local governments' provides a brief review of some of the supportive evidence for this recommendation, with a focus on boosting social cohesion and reducing inequalities in access. | |  | |

| **Supplementary Table 3A – Extraction of WHO and Cochrane evidence from search (iii)** | | | | | |
| --- | --- | --- | --- | --- | --- |
| **Air Pollution** |  |  |  |  |  |
| **WHO Literature** | | | |  |  |
| **Title** | **Summary** | **Methodological Approach** | **Population-level Recommendations** | **Relevant Cited Evidence** | **Notes** |
| WHO global air quality guidelines: particulate matter (‎PM2.5 and PM10)‎, ozone, nitrogen dioxide, sulphur dioxide and carbon monoxide (2021) | Establishes recommended guidelines for 'safe' levels (concentration and length) of exposure to common pollutants, below which there is no significant health risk | Based on systematic reviews of observational evidence | Explicitly does not make recommendations for interventions, claiming that 'what works' will be context dependent. However, does signpost to a Cochrane review | WHO, 2019. Personal interventions and risk communication on air pollution: summary report of WHO expert consultation, 12-14 February 2019  Burns, 2019. Interventions to reduce ambient particulate matter air pollution and their effect on health. Cochrane Review  Janjua 2021. Individual‐level interventions to reduce personal exposure to outdoor air pollution and their effects on people with long‐term respiratory conditions. Cochrane Review  PHE, 2020. Review of interventions to improve outdoor air quality and public health: Principal interventions for local authorities |  |
| Compendium of WHO and other UN guidance on health and environment (2022 update) | This compendium consists of a systematic compilation of WHO and other UN guidance that addresses all major areas of health and the environment into one resource. This compendium presents a repository, extracting the relevant guidance for policy-makers | This compendium was developed by systematically compiling existing published guidance on health and the environment from WHO and other UN organizations. Relevant WHO technical units were systematically consulted on structure, content and resources. The units’ inputs and subsequent reviews were incorporated. Relevant other UN organizations, responsible for content directly related to health and the environment, were also consulted for their inputs and review  Compendium itself does not report effect sizes, specific interventions, or reference any direct empirical evidence (only other guidance) | Ambient Air Pollution Extensive list of recommendations, too large to reproduce. Not specific interventions, no empirical references provided (just signposting to other guidance), and no effect sizes. Sub-headings of intervention types: - Transport systems (n=5) - Industry (n=7) - Power generation (n=3) - Waste and wastewater management (n=5) - Agriculture (n=5) - Housing (n=2) - Land use (n=3) - Other (n=3) - Awareness raising and capacity building (n=3) | Ambient Air Pollution *[18] WHO 2011, Health in the green economy. Health co-benefits of climate change mitigation - Transport sector.* Mostly summarises modelling evidence from IPCC and others, but includes some empirical case study examples (though study selection approach unclear).  *[19] WHO & UN Habitat, 2020. Integrating health in urban and territorial planning: sourcebook for urban leaders, health and planning professionals.* Implementation guide *[20] WHO, 2016. Health as the pulse of the new urban agenda. United Nations Conference on Housing and Sustainable Urban Development.* Meeting report with some case studies, but no formal evidence review *[21] UN Environment Programme, 2019. Air pollution in Asia and the Pacific: science-based solutions.* Comprehensive status report for pollution in Asia, with modelling of possible options by parameterising Amann et al.'s 2011 European tool to the region. Amann's model appears to be assumption-based regarding intervention effectiveness, rather than grounded in empirical interventional evidence *[22] UN, 2016. Guidance document on emission control techniques for mobile sources under the Convention on Long-Range Transboundary Air Pollution.* Provides recommendations but without evidence, with the exception of 3 citations for high-polluting old car exchange programmes with financial incentives, one of which has empirical evidence in the form of case studies: *Older Gasoline Vehicles In Developing Countries and Economies in Transition: Their Importance and the Policy Options for Addressing Them [23] UN Environment Programme, 2020. Used vehicles and the environment.* Summarises environmental costs of old vehicles in LMICs, and provides case study examples, and high-level comparisons of, current policy approaches. No interventional empirical evidence *[24] WHO Europe Regional Office, 2020. Human health in areas with industrial contamination.* Book summarising harmful effects of pollution on health *[25] WHO Europe Regional Office, 2020. Waste and contaminated sites.* Can't find *[26] WHO, 2020. Risk communication and personal level intervention to reduce exposure and to minimize the health effects of air pollution*. See below *[27] WHO Europe Regional Office, 2017. Urban green spaces: a brief for action.* Summary of an expert consensus meeting [28] *WHO Europe Regional Office, 2017. Urban green spaces interventions and health - a review of impacts and effectiveness.* Non-systematic evidence review of green space interventions. Included studies very low quality, and none measured changes to ambient air pollution. [29*] WHO, 2018. Global action plan on physical activity 2018–2030: more active people for a healthier world.* Presents the action plan. Evidence described in generality [30] *IPCC, 2019. Special report on climate change and land: an IPCC special report on climate change, desertification, land degradation, sustainable land management, food security, and greenhouse gas fluxes in terrestrial ecosystems.* Extensive report focused on land use and climate change. Not all referenced, and does not appear to be any empirical, interventional evidence showing a reduction in pollution *[31] WHO, UNEP, 2010. Healthy environments for healthy children: key messages for action.* No empirical evidence | Reflective that much of this evidence base is modelling based, and/or based upon technical data (i.e. studying a fuel type in a lab setting and assuming this will translate to real-world settings); rather than empirical studies of interventional evidence - which can be difficult to do given the nature of the exposure |
|  |  |  | Indoor Air Pollution Extensive list of recommendations, too large to reproduce. Not specific interventions, no empirical references provided (just signposting to other guidance), and no effect sizes. Sub-headings of intervention types: - General (n=3) - Clean fuels and technologies (n=12) - Housing (n=2) - Awareness raising and capacity building (n=4)  The referenced WHO 2014 guidelines do include empirical estimates and are summarised below | Indoor Air Pollution *[21] UN Environment Programme, 2019. Air pollution in Asia and the Pacific: science-based solutions.* As above *[37] WHO, CCAC, UNEP, 2018. Breathelife campaign.* Can't find this report *[51] WHO, 2014. WHO guidelines for indoor air quality: household fuel combustion (see below) [52] WHO, 2021. Household air pollution: identifying solutions for countries.* Appears to be a signposting page on WHO website *[53] International Organization for Standardization, 2018. Clean cookstoves and clean cooking solutions — Harmonized laboratory test protocols — Part 1: Standard test sequence for emissions and performance, safety and durability.* Provides guidance on how to measure emissions from cooking stoves in a standardised way *[54] WHO, 2019. Ambient air pollution: interventions & tools.* Webpage |  |
| WHO guidelines for indoor air quality: household fuel combustion (2014) | Development of these guidelines included an SR of clean fuel interventions for people currently using biomass or coal as the primary cooking fuel, with open fires or traditional stoves. And an overview of SRs considering case studies and qualitative evidence of what factors influence the household uptake of cleaner cooking stoves | Made recommendations for research and policy using a meta-analysis, and a comprehensive weighing up of the strength of evidence. Recommendations based on 38 experimental studies measuring changes too kitchen PM (n=27), personal PM (n=3), kitchen CO (n=26), and personal CO (n=5). Provides separate effect sizes/meta-analysis estimates for stove type, and for study design | Solid fuel stoves with chimneys Kitchen PM2.5: -0.46 μg/m3, (95% CI -0.60, -0.33). n=12 CBA studies. Moderate confidence Kitchen CO: -5.7 μg/m3 (95% CI -3.9, -7.5). n=10 CBA studies, 1 RCT. Moderate confidence  Solid fuel stoves without chimneys Kitchen PM2.5: -0.26 μg/m3 (95% CI -0.12, -0.39). n=4 CBA studies. Low confidence Kitchen CO: -3.32 μg/m3 (95% CI -1.86, -4.77). n=4 CBA studies. Moderate confidence  Advanced combustion solid fuel stoves Kitchen PM2.5: -0.22 μg/m3 (95% CI -0.06, -0.38). n=1 CBA study. Low confidence Kitchen CO: -3.16 μg/m3 (95% CI -0.73, -5.59). n=1 CBA study. Low confidence  Ethanol Kitchen PM2.5: -0.58 μg/m3 (95% CI -0.24, -0.92). n=2 CBA studies. Low confidence Kitchen CO: -27.3 μg/m3 (95% CI -13.1, -41.5). n=2 CBA studies. Low confidence  Does not report any results for the personal monitoring studies. Notes that, even with the reductions seen for the solid fuel stoves, they don't meet the WHO targets. More RCTs required, and more using advanced stoves and LPG. The review of contextual factors finds that no factors guarantee success, but to be widely adopted, interventions must take relevant contextual factors into account e.g. can the traditional foods be cooked on the provided stove, is there long-term support for replacing parts, is the fuel supply reliable to that setting | SR with reference list.   Though we did not specifically solicit extra evidence for this risk factor, two relevant studies were identified through professional networks: 1. *Ye W, 2022. Effects of a Liquefied Petroleum Gas Stove Intervention on Gestational Blood Pressure: Intention-to-Treat and Exposure-Response Findings From the HAPIN Trial. Hypertension.* This RCT randomised 3,195 pregnant women using biomass stoves in Guatemala, India, Peru, and Rwanda to receive either an LPG stove or waiting list controls. This study measured 24-h personal PM2.5, CO and Black Carbon at baseline and at two follow-up visits. The controls PM2.5 at baseline, follow up 1, and follow up 2 were: 112μg/m3 (SD 107.8), 104.0 (112.3), 102.3 (107.9). In comparison, the intervention group: 120.4 (134.0), 33.9 (33.2), 35.8 (54.8). The authors don't report a difference-in-differences value, but they state that a t test comparing intervention and control groups at the follow-ups were significant at the 0.001 level (this was also the case for CO and black carbon)  2. *Walker E, 2022. Efficacy of air filtration and education interventions on fine particulate matter among rural Native American homes heated with wood stoves: Results from the EldersAIR randomized trial. Science of the Total Environment.* This intervention involved the installation of air filtration devices, rather than clean cooking stoves. It was an RCT involving older people in Native American communities in the US, and it reported a 50.5% reduction in the geometric mean of the kitchen PM2.5 concentrations (95% CI 27.8%, 66.1%) across consecutive winter seasons; and 44.7% (1.2%, 69.0%) for personal PM2.5 | Clear evidence for a recommendation about installing cleaner cooking stoves, given the overall weight of evidence, with consistent evidence from across several countries and contexts, and now further RCT evidence added - suggest high confidence recommendation.   Only one study for air filtration devices, suggest this is included in the detailed information around the intervention only. |
| Draft Updated Appendix 3 and Technical Annex (2022) | Update of the 2017 version of the Appendix 3 of the WHO Global NCD Action Plan 2013-2020, now extended until 2030, considering new scientific evidence as well as new WHO recommendations, since the 2017 update | Updated following consultation, seeking examples of interventions using the following criteria: "(1) An intervention must have a demonstrated and quantifiable effect size, from at least one published study in a peer reviewed journal. (2) An intervention must have a clear link to one of the global NCD targets"  Analysed by WHO-CHOICE methodology | Access to improved stoves and cleaner fuels to reduce indoor air pollution | No evidence provided to support recommendation (but see above) |  |
| Personal interventions and risk communication on air pollution: summary report of WHO expert consultation (12-14 February 2019) | Summary reports of an expert consultation in 2019 on interventions that individuals can take to reduce their risk, and mechanisms of communicating the health risks of pollution | Consensus view of a commission that has "examined" the evidence | n/a | n/a |  |
| Evidence-based strategies to reduce the burden of household air pollution in Accra, Ghana (2021) | Summarises a modelling exercise of more aggressive roll out of a subsidised conversion programme from charcoal and firewood to liquified petroleum gas in homes and small businesses in Accra, Ghana | Model inputs include: - Data on emission rates of the various fuels - Data on the proportion of indoor pollutants attributable to the kitchen, from local field work (peer reviewed) - Survey and government data to assess current fuel usage and conversion rates - A WHO for estimating health impacts avoided from COPD, resp infections, IHD, lung cancer, and stroke, using best available prevalence estimates for these conditions from GBD | Subsidised, national scheme for the replacement of charcoal and firewood cooking stoves with liquified petroleum gas in homes and small businesses. Most aggressive scenario modelled estimated 71,000 DALYs and 1,922 deaths would be averted by 2030. | Estimates behind the model which equate reductions in pollution to health effects are not directly referenced here |  |
| Indoor air pollution and lower respiratory tract infections in children (2007) | A report which examines the effect of indoor air pollution on respiratory tract infections in children, including reporting the findings of a cluster RCT in Guatemala | Randomised 518 households in Guatemala, which used an open fire for cooking at baseline and contained a pregnant woman or child under 4 months, to an improved chimney stove at the start of the study (intervention group) or when the child reached 18 months old (control group). Children and mothers wore 48-hour CO monitors every 3 months of the study | WHO report -  44% reduction (95% CI 39% to 48%) in child exposure to CO associated with improved stove (model unspecified, but adjusted for age, gender, season, and weekday) Intervention group: baseline mean CO 2.4ppm (+/- 2.4 ppm) reduced to 1.0 (+/- 2.3)  Control group: baseline mean CO 2.5ppm (+/- 1.8ppm) reduced to 2.0 (+/- 2.2) Unclear if +/- represents CIs or SE  2009 Paper in J of Exposure Sci & Env Epi -  52% reduction (95% CI 47% to 56% reduction) for child exposure to CO (fixed effects model) This paper also reports on reductions for mothers: 61% (57, 65), and the kitchen area: 90% (87, 92) This papers also reports results of a sub-study which examined the relationship between CO and PM2.5 via continuous monitoring in a sub-sample of households. They found that 8ppm of CO was roughly equivalent to 1mg/m3 of PM2.5 | Source data from a pilot RCT (RESPIRE) which aimed to demonstrate an ultimate reduction in respiratory tract infections. Subsequently written up and published in three publications: McCracken J et al., 2009. Combining Individual- and Group-Level Exposure Information: Child Carbon Monoxide in the Guatemala Woodstove Randomized Control Trial. Epidemiology  Smith K, 2009. Personal child and mother carbon monoxide exposures and kitchen levels: Methods and results from a randomized trial of woodfired chimney cookstoves in Guatemala (RESPIRE). Nature J of Exposure Science & Env Epi  Smith K et al., 2011. Effect of reduction in household air pollution on childhood pneumonia in Guatemala (RESPIRE): a randomised controlled trial. Lancet | N.B. these estimates are included in the 2014 WHO report above  Any problems with the stoves during the study were repaired (free of charge) by the study team |
| **Cochrane** | | | | |  |
| **Title, Author** | **Summary** | **Details of the population-level interventions** | **Findings** | **Recommended interventions** | **Notes** |
| Interventions to reduce ambient particulate matter air pollution and their effect on health (Burns, 2019) | Cochrane review of 42 studies of 38 unique interventions which used time series, or pre-post, analyses (almost all with a control group area) to assess the effect of interventions to reduce pollution, with or without measured health outcomes | **Industrial:** cap and trade programmes (n=1), factor closure (n=2), power plant conversion from oil to gas (n=1), mandatory industry requirements (n=1) **Residential:** stove exchange with financial incentive/maintenance assistance (n=3), comprehensive coal ban (n=3), wood burning ban during times of poor air quality (n=1) **Vehicular:** congestion zone charge (n=2), speed limit restriction fixed or variable (n=3), low emission zones (n=3), even-odd restrictions (n=3), infrastructure changes (n=6), bans on high-polluting vehicles or fuels (n=4), switching public transport vehicles from diesel to natural gas (n=1) **Multiple:** multiple restrictions on high-pollution days (vehicular restrictions, residential heating restrictions, postponing street sweeping and traffic enforcement activities) (n=2), city-wide campaign (including low emission zone, even-odd ban, industrial restrictions) (n=1)  HICs n=30, LMICs n=12 Mixed quality but several at low risk of bia**s** | **Industrial:** 1 study from the US, at low ROB, using ITS design, found that the cap and trade programme resulted in a 7.2% reduction in NO2, and 5.8% reduction in O3. 1 study from Israel, at serious ROB, found that conversion of a power plant fuel source from oil to gas in Israel resulted in a 14% reduction in PM10, with a 31% increase noted in control sites. 1 study from Australia, at serious ROB, using an ITS design, found that closure of a steel works in Australia resulted in a 40.5% reduction in SO2 levels **Residential:** 3 ITS studies from Ireland, all at low ROB, of comprehensive coal bans, were assessed only against health outcomes, showing significant reductions in respiratory mortality (16.8%) in one study, and respiratory (8.5%) and cardiovascular (3.2%) hospitalisations in another study, and a non-significant trend towards reduced respiratory mortality in the other study. 3 studies, from Australia, Canada, and India, all at some or serious ROB, found that stove exchange programmes did not change ambient air pollution or health outcomes. 1 study from the US, at some risk of bias, found that an air-quality dependent wood burning ban resulted in a 12.3% reduction in PM2.5, and 8.5% reduction in coarse PM, as well as a 7% decrease in cardiovascular hospitalisations in people over 65.  **Vehicular:** 3 studies from Netherlands and Germany, all at low ROB, using ITS/CBA designs, found that low emission zones were associated with significant reductions in pollutants (Netherlands, ITS design, 10.4% greater reduction in PM2.5 than control sites. Germany, CBA design, PM10 19.6% reduction in summer, 6.8% in winter. Germany, CBA design, 3.5% NOx, 2.2% NO2, 2.3% NO reductions). 3/4 studies of vehicle/fuel bans found significant benefits (1 low ROB from Japan, ITS, found mandatory standards for diesel cars resulted in a 2.1% reduction in all-cause mortality, 5.9% cardiovascular mortality, and 10% respiratory mortality), 1 study at some ROB, and 1/2 studies at serious ROB also found significant benefits. 2/3 studies, both low risk of bias, of even-odd restrictions found a reduction in pollutants (Beijing, ITS, PM10 31% immediate, 27% sustained reduction. Ecuador, CBA, 9% CO reduction); whilst two studies looked at the same intervention in Mexico, both at some risk of bias, found mixed results. 2/3 speed limit studies found significant reductions in pollutants (Netherlands, CBA design, low risk of bias, 7.4% reduction in PM10. Spain, ITS design, some risk of bias, 14.7% reduction in PM10, 16% reduction in NOx); but the other found a significant increase (ITS at some risk of bias, PM10 5.4% increase, NOx 1.7% increase). Mixed findings for infrastructure changes, with four studies reporting no significant pollutant reduction, and two studies, at some risk of bias, reporting reductions in black carbon (37% compared to 14% at controls; and 72% compared to 6% at controls) resulting from a major road reconstruction in Slovenia, and a public bus system restructuring in Spain. Neither congestion charge study found significant reductions in pollutants (at some/serious risk of bias). Changing public bus fuel to natural gas was not associated with reductions in pollutants in South Korea (some risk of bias).  **Multiple:** Both studies examining multiple restrictions on high-pollution days reported reductions in pollutants (Chile, low ROB, ITS, 16.9% reduction in PM10. USA, some ROB, CBA, 2.3% reduction in O3). One study, some ROB, China, ITS, reduction in respiratory hospitalisations (RR 0.5) | Commenting on the totality of evidence, the authors note mixed findings - particularly for studies attempting to directly measure health outcomes  Considering studies at low ROB by individual intervention types: - 3 studies found that low emission zones found reduced PM2.5 (10.4%), PM10 (6.8% winter, 19.8% summer), and nitrous oxides (2.2-3.5%) - 3 studies found that comprehensive (marketing, sale and distribution) coal bans for residential heating reduced respiratory mortality (16.8%), respiratory hospitalisations (8.5%), and cardiovascular hospitalisations (3.2%) - 2 studies found that even-odd restrictions reduced PM10 (27%), and CO (9%) - 2 studies found that multi-components restrictions (driving, shutdown of certain major stationary emitters, street sweeping, traffic enforcement activities, and restriction on the use of biomass combustion for residential heating) on high-pollution days reduced PM10 (16.9%), an d hospitalisations (RR 0.5) - 1 study found that a cap and trade programme reduced NO2 (7.2%), and O3 (5.8%) - 1 study found that speed restrictions reduced PM10 (7.4%) | N.B. In reference to the recommended intervention by WHO: 3 studies, from Australia, Canada, and India, all at some or serious ROB, found that stove exchange programmes did not change ambient air pollution or health outcomes |
| Individual‐level interventions to reduce personal exposure to outdoor air pollution and their effects on people with long‐term respiratory conditions (Janjua, 2021) | Cochrane review of 11 studies of individual-level interventions to reduce exposure to outdoor air pollution | None. 1 study involved participants cycling home from work on a route which had a lower proximity to traffic. However, the intervention was instructing the individuals to do this, rather than provision of this route.   All other studies involved the use of face masks, or alert/education-based interventions | The alternative cycling route study was at serious ROB. The intervention was preferred by 66% of participants, and resulted in a reduction in nasal/throat irritation but no change on several other measures of respiratory symptoms | n/a |  |
| Organisational travel plans for improving health (Hosking 2010) | Cochrane review of 17 studies of organisational travel plans in schools (n=10), universities (n=2), and workplaces (n=5) and the effects on health and travel mode | **Schools:** Local walking/cycling infrastructure improved near to school, alongside education and encouragement (n=2), individual-level interventions only (n=3), intervention described only as presence/absence of having a plan - with the plan components unclear (n=5)  **Workplace:** Shorter working week to reduce commuting (n=1), advice and encouragement with provision of reflective safety accessories (n=1), individual-level only (n=3)  **University:** Facilitating and reserving car parking spaces for car-pooling (n=1), individual-level only (n=1) | No studies measured pollution outcomes. 1 study (workplace, reflective safety accessories + individual-level advice/encouragement) measured quality of life and found significant improvements in some sub-scales (mental health, vitality, general health) but not 5 other sub-scales  Most studies measured change in transport usage and reported reductions in car usage which could be hypothesised to result in reductions in pollution. However, most of these studies were judged at high-risk of bias (e.g. many used pre-post survey designs). Studies judged to be at lower risk of bias reported null or mixed results | n/a | The authors note that such plans could be considered as part of wider approaches to increase active travel and reduce vehicular usage (and therefore potentially pollution) |
| **Traumatic Brain Injury** | | | |  |  |
| **WHO Literature** | | | | | |
| **Title** | **Summary** | **Methodological Approach** | **Population-level Recommendations** | **Relevant Cited Evidence** | **Notes** |
| Compendium of WHO and other UN guidance on health and environment (2022 update) | This compendium consists of a systematic compilation of WHO and other UN guidance that addresses all major areas of health and the environment into one resource. This compendium presents a repository, extracting the relevant guidance for policy-makers | This compendium was developed by systematically compiling existing published guidance on health and the environment from WHO and other UN organizations. Relevant WHO technical units were systematically consulted on structure, content and resources. The units’ inputs and subsequent reviews were incorporated. Relevant other UN organizations, responsible for content directly related to health and the environment, were also consulted for their inputs and review | Describes recommendations in generality, without specific reference to empirical evidence showing a benefit | Several guidelines, including those extracted below |  |
| Helmets: a road safety manual for decision-makers and practitioners, 2nd edition. (2023) | Part of a series intended to provide guidance on implementation of interventions to address specific risk factors in road safety, supporting the implementation of good practices in road safety to help make the world’s roads safer for all | A review of the evidence on risk factors and interventions was conducted for information for revision of this manual. The review utilized text mining techniques to gather evidence on risk factors and outcomes of interventions. This technique creates computational algorithms for reading and extracting texts from a large volume of information in a short period of time. The review was limited to literature from Jan 2008-Dec 2019, with the understanding that the previous manual had drawn on the evidence that existed before January 2008. Only papers in English, French, Portuguese and Spanish were included. Studies excluded were those presented in conference proceedings, editorials and draft papers. The full search generated 157 abstracts relevant to PTW safety, including helmet use, which were screened to produce 53 full studies for review for this manual. The two experts who conducted the literature review grouped the interventions into three categories – proven, promising and insufficient evidence – based on existing best practices in road safety. The Advisory Committee reviewed the categories and refined them based on the existing best practices in road safety policy and their expert knowledge | **Proven Interventions:** 1. Helmet usage: SR evidence suggests they reduce risk of head injury by 58–60%, and brain injury by 47–74% (roughly in line with earlier Cochrane reviews)**.** Also describe evidence showing full face helmets, and firm fitting helmets, are relatively more protective than alternatives 2. Helmet legislation: An SR on legislation for mandatory motorcycle helmet use (Peng, 2017, Am J Prev Med) reports a median reduction of –54% (–49% to –59%) in head injuries from 5 studies in the USA. The WHO report also cites several single studies from a mix of other countries including LMICs demonstrating effectiveness of legislation. WHO also note examples were legislation has been insufficient due to lack of supporting interventions such as effective enforcement with fines for non-compliance, a ready supply of affordable helmets, and sustained mass media campaigns for the public and education for the police | Extensive - see references 34-78. This includes the Cochrane review by Liu below | Also provides helpful, and comprehensive, implementation guidance |
|  | Reviewing the other manuals in this series (which were not themselves direct hits in the search). Outside of recommending helmet usage, none provide any specific data on reduction of head or brain injuries, though several of the reports make the logical link between improving road safety and reducing injuries including head injuries  Pedestrian safety (2023) - recommended evidence-based interventions include reducing pedestrian exposure through pavements, road and infrastructure design (e.g. overpasses), reducing road traffic through public transport improvements; reducing vehicle speeds; improving visibility through road design; and improving driver behaviour  Drink driving (2022) - recommended evidence-based interventions include making it illegal to drive above set alcohol concentrations with criminal charges and revoking license, minimum drinking age, mass media and rehabilitation  PTW (2022) - aside from helmets (see above) recommended evidence-based interventions include compulsory skill test for a permit, and exclusive motorcycle lanes. In reference to a secondary outcome of the study, a study on the effect of bicycle helmet use is cited: Dodds, 2019, BMJ Open. Which used UK trauma database data to estimate that cycle helmet use was associated with a reduction in severe traumatic brain injury (TBI) of 19.1% (780, 18.0%–20.4%)  Cyclist safety (2020) - helmets are recommended with evidence drawn from Cochrane (see below). Aside from helmets, evidence-based recommendations include speed restrictions for other vehicles, and segregated bike lanes with intersections   Save Lives - a road safety technical package (2017) - overarching summary of the 1st edition of this series, with succinct recommendations and implementation guidance   Strengthening Road Safety Legislation (2013) - useful implementation guide for legislative measures, including helmets  Speed management (2008, no update) - recommended evidence-based interventions include setting and enforcing legal speed limits, road design for traffic calming, limiting maximum speed on new cars, public education  Seatbelts (2009, no update) - states that seatbelts reduce head injuries specifically and cite a Norwegian government report (no longer available online) which estimates 60% of injuries sustained in car crashes are head injuries. However, quoted empirical evidence is for fatalities and general injuries (though could extrapolate from the 60% estimate). Recommended evidence-based interventions include mandated seatbelts and child car seats  Data systems (2010, no update) - focussed on systems to capture trends in road traffic incidents | | | | |
| **Cochrane** | | | | |  |
| **Title, Author** | **Summary** | **Details of the population-level interventions** | **Findings** | **Recommended interventions** | **Notes** |
| Bicycle helmet legislation for the uptake of helmet use and prevention of head injuries (Macpherson, 2008) | Cochrane review of 6 studies assessing the effect of legislative changes on head injuries (n=3), mortality (n=1), or helmet use (n=3) | Introduction of legislation to mandate helmet usage for child cyclists (maximum age 16, 17, or 18 in different studies). All studies from the US (Georgia n=1, California n=2) or Canada (Alberta n=1, Ontario n=1, Canada-wide comparison of provinces with a law and without n=1). Outcome data were trauma registries (n=1), hospital (n=2) or mortality (n=1) data, or serial observations (n=2). Control group was either pre-intervention data (n=2), adults (n=3), or children in control provinces (n=1) | 2/3 studies reported a reduction in head injuries: - In Canada, rates of head injuries in provinces with and without legislation were similar pre-intervention (18.27 and 18.35 per 100,000 respectively). Post-legislation the decline in the intervention provinces was significantly greater (P < 0.001) than for controls (45% reduction in head injuries in intervention provinces, 27% decline in controls. No significant differences in the change in non‐head injuries between provinces with and without legislation (P = 0.11) - In California, the proportion of hospitalised injuries that were traumatic brain injuries among youth decreased after legislation (OR 0.82; 99% CI 0.76 to 0.89) with no change for adults. Greatest change in children under 10 - In San Diego, California. A downward trend in hospitalisation for serious head injury was noted, but this did not reach statistical significance  A significant decrease was reported in 1/1 study of effect on mortality, and significant increases were reported in 3/3 studies of effect on helmet usage  Studies generally scored well on risk of bias assessment, given the designs used | Legislation to mandate the use of helmets for child cyclists | One study (Gilchrist et al.), not included in the Owen review below, also included provision of helmets to school children in the study area (alongside the legislation to mandate helmet usage in Georgia, with police enforcement). This study reported a significant increase in helmet usage amongst children compared to adults |
| Non‐legislative interventions for the promotion of cycle helmet wearing by children (Owen, 2011) | Cochrane review of 29 studies assessing the use of non-legislative interventions for the promotion of helmet use in schools (n=19), healthcare settings (n=7), and the community (n=4) | Distribution of free (n=9) or subsidised helmets (n=11). The other studies were based on health education only. All interventions were targeted at children. All studies came from high income countries | In meta-analysis, compared to controls, providing free helmets resulted in greater observed helmet wearing (two studies: OR 4.35, 95% CI 2.13 to 8.89; Chi2 = 0.47, df = 1, P = 0.49). The effect of providing subsidised helmets failed to reach statistical significance and there was significant heterogeneity between effect sizes (seven studies: OR 2.02, 95% CI 0.98 to 4.17; Chi2 = 20.29, df = 6, P = 0.002). No evidence of publication bias  Similar effects of free helmets were noted for self-reported helmet ownership and self-report helmet usage - data on subsidised helmet usage not amenable to subgroup analysis. Significant benefits observed in schools, healthcare settings, and community-based projects. Interventions for children under 12 were effective, but for those 12-18 did not reach statistical significance. One study reported that free helmet provision in an underserved community reduced inequalities in helmet wearing observed at baseline. Mixed quality evidence, results non-significant when limited to RCTs, but these were mainly health education studies | Providing free helmets (insufficient evidence to recommend subsidised helmets)  Those set in schools appear to be effective but possibly less so than community‐based interventions  Interventions may be more effective if provided to younger rather than older children | A change in head injuries was not measured directly. Whether helmets were being worn correctly was not measured. Whether cyclists take more risks when wearing helmets was not measured. However, the review by Thompson (see below) provides supportive evidence for each of these questions |
| Helmets for preventing injury in motorcycle riders (Liu, 2008) | Cochrane review of 61 observational studies of the effect of motorcycle helmet usage on death (n=30) or head injury (n=36) amongst crash victims | None - the exposure is simply whether helmets were in use at the time of the crash or not | Motorcycle helmets were found to reduce the risk of death and head injury in motorcyclists who crashed. From six higher quality studies helmets were estimated to reduce the risk of head injury by 69% (OR 0.31, 95% CI 0.25 to 0.38), and from four higher quality studies helmets were estimated to reduce the risk of death by 42% (OR 0.58, 95% CI 0.50 to 0.68)   Studies were of varying quality, however, despite methodological differences there was a remarkable consistency in results | It is possible to infer from these results that motorcycle helmets should be mandated for all riders. However, to estimate the potential effect size of this legislation, one would have to know the pre-legislation prevalence of motorcycle helmet usage, and what % increase in usage was feasible from the intervention | Note the intervention here is usage of a motorcycle helmet, not population-level interventions to increase their usage. However, this is a useful review because it countered arguments which suggested that helmet usage may reduce rider vision |
| Helmets for preventing head and facial injuries in bicyclists (Thompson, 1999) | Cochrane review of 5 case-control studies of the effect of helmet usage on head injuries | None - the exposure is simply whether helmets were in use at the time of the crash or not | Studies scored well on risk of bias assessment | Helmets provide a 63 to 88% reduction in the risk of head, brain and severe brain injury for all ages of bicyclists | Note the intervention here is usage of a motorcycle helmet, not population-level interventions to increase their usage. However, this is a useful review because it supports the evidence described by Owen et al. |
| Population‐based interventions for the prevention of fall‐related injuries in older people (McClure, 2005) | Cochrane review of 6 studies of studies of community-based interventions for the prevention of falls amongst older people | 5/6 interventions were multi-component and contained population-level components, including: home hazard reduction by a healthcare worker (n=5), public hazard reduction (n=1), training for healthcare workers, council workers and house planners to reduce hazards in relevant settings (n=1), and environmental improvements such as improved walkway quality or increased lighting (n=2) | All studies reported significant reductions in falls-related injuries, with effect sizes ranging from 6 to 33% reductions, relative to controls. However, no study reported a specific effect on head injuries - though a cited study in the introduction states that 5-10% of all falls in the elderly result in a serious injury such as a head injury, so this could be extrapolated. All were controlled before/after studies that scored well on risk of bias assessment | n/a | Difficult to make a specific recommendation as it is unclear which population-level components were associated with benefits, and whether these would have conveyed specific reductions in traumatic brain injury. Also doesn't fit the lifecourse model heading injury as a midlife risk factor as this is only in the older age group  Protocol for an updated review published in 2020 |
| **Depression** |  |  |  |  |  |
| **WHO literature** | | | | | |
| **Title** | **Summary** | **Methodological Approach** | **Population-level Recommendations** | **Relevant Cited Evidence** | **Notes** |
| WHO menu of cost-effective interventions for mental health (2021) | Menu of cost-effective interventions for mental health for which information on cost-effectiveness is available. It is preliminary and not exhaustive; and includes both population- and individual-level interventions | Method for selection of interventions not described  Cost-effectiveness analysis done with WHO-CHOICE methodology | Regulatory bans on the use of highly hazardous pesticides to prevent suicide. Note this is not relevant for our purposes as the outcome is suicide, rather than a reduction in depression (the mechanism being that many people use fertilisers when attempting to take their own life, rather than fertilizers having a neurochemical effect that worsens mental health) | n/a | Two interventions which WHO describe as population-level do not meet our definition - both based on school-based socioemotional learning to improve mental health and prevent suicide. Psychological support and medication for several psychiatric conditions are listed as individual-level interventions. |
| **Cochrane** |  |  |  |  |  |
| **Title, Author** | **Summary** | **Details of the population-level interventions** | **Findings** | **Recommended interventions** | **Notes** |
| Interventions to improve return to work in depressed people (Nieuwenhuijsen, 2020) | Cochrane review of 45 studies of interventions to improve return to work in depressed people - with some studies reporting depressive symptoms as an outcome | Work directed-interventions alone (n=4) or combined with clinical interventions (n=13) were based around the intervention providers working as an intermediate with the workplace to structure a return-to-work plan which reduced barriers to work for the depressed person, and could therefore be considered to meet our definition of population-level | Work-directed interventions were only associated with a reduction in depressive symptoms when combined with a clinical intervention. When instigated alone, there was a non-significant reduction in depressive symptoms in the first year, with a null effect beyond 1 year | n/a |  |
| Organisational interventions for improving wellbeing and reducing work‐related stress in teachers (Naghieh, 2015) | Cochrane review of 4 studies of organisation-based interventions to reduce stress in teachers, with one study measuring depressive symptoms | None - the study which measured depression as an outcome was based on resilience training for the school children | There was no statistically significant change in job-related depression at 12 or 24 months | n/a |  |
| Later school start times for supporting the education, health, and well‐being of high school students (Marx, 2017) | Cochrane review of 11 studies of later school start times on academic outcomes, sleep, and depressive symptoms (n=1) | Delayed morning (one study also considered the effect of delaying the afternoon session) start time for middle- or secondary- school | In one study from Minnesota US, using a CBA design, there was a significant reduction in the depressive symptoms subscale of a sleep scale for those children starting at 7:25 or 8:30 compared to 7:15 (F(2,412) = 11.49, P<0.001). However, there was no significant difference reported when comparing the longer delay between 7.25 and 8:30 | Insufficient evidence to recommend this intervention from a depression perspective |  |
| Psychological and social interventions for the prevention of mental disorders in people living in low‐ and middle‐income countries affected by humanitarian crises (Papola, 2020) | Cochrane review of 7 RCTs of psychosocial interventions to prevent mental disorders including depression in LMICs affected by humanitarian crises | None. Most were group-based psychotherapy. One study included establishment of a short-term football competition, but this was not accompanied by any sustained structural changes to improve access to sports | The sports-based study reported a worsening in depressive symptoms in the intervention group compared to the waiting list control. In general the results of all studies were null | n/a |  |
| Physical environmental designs in residential care to improve quality of life of older people (Harrison, 2022) | Cochrane review of 20 studies (RCTs n=7, CBA n=12, cohort n=1) from HICs of changing the physical environment of residential homes, with or without changes to the functioning of the setting, on quality of life and related measures including depression (n=2) and social engagement (n=2) | The review contains various interventions including re-designs, better lighting, garden refurbishment. But the two studies which included depression as an outcome were part of the majority design which compared small-scale more home-like environments to larger homes | There was no statistically significant evidence of a reduction in depressive symptoms in the intervention group | n/a |  |
| Community‐based interventions for improving mental health in refugee children and adolescents in high‐income countries (Soltan, 2022) | Cochrane review of 3 RCTs of community-based interventions on mental health outcomes in refugee children | None. One trial was art therapy, one was music therapy, and one was CBT - all group-based | The trials all reported null results on PTSD, depressive symptoms, and psychological distress (n=1 study for each) | n/a |  |
| Unconditional cash transfers for reducing poverty and vulnerabilities: effect on use of health services and health outcomes in low‐ and middle‐income countries (Pega, 2022) | Cochrane review of 34 studies of unconditional cash transfers for poverty alleviation in LMICs on various health outcomes, including depression (n=11) | Studies conducted in Colombia, Ecuador, Indonesia, Lesotho, Mexico, Nigeria, South Africa, Uruguay, Zambia, Zimbabwe, Burkina Faso, Kenya, Malawi, DRC, India, Kenya, Malawi, Zimbabwe  Interventions varied between pension schemes, child support grants, and welfare safety nets | All 5 cluster-RCTs reported non-statistically significant effects on depressive symptoms (follow up range 7 to 47 months). 2/3 and 1/1 CBA and cohort studies reported small but statistically significant benefits. Studies considered at high risk of bias | Given mixed results, with null results for higher quality studies, it is not possible to recommend this intervention for reduction of depression |  |
| Unconditional cash transfers for assistance in humanitarian disasters: effect on use of health services and health outcomes in low‐ and middle‐income countries (Pega, 2015) | Cochrane review of 3 studies of unconditional cash transfers on health service use | UCT provided to mothers in poor households in Niger (n=2) and Nicaragua (n=1). Only 1 of the studies (from Niger) reported an effect on adult depression using CES-D | Null effects | n/a |  |
| Welfare‐to‐work interventions and their effects on the mental and physical health of lone parents and their children (Gibson, 2018) | Cochrane review of 12 studies of interventions to move single parents (almost exclusively mothers) from welfare support into work - to reduce welfare payments (n=3), or to alleviate poverty by ensuring work paid more than welfare (n=8) - on physical and mental health of the parent and children | Studies either aimed for rapid integration into the workforce ('a work-first approach') (n=8) or for a skills and development-based approach (n=4). Other positive components included earning supplements, childcare subsidies, and health insurance subsidies; whilst several studies also used punitive measures to reduce access to welfare payments or mandate activities such as job applications. All studies were conducted in North America | Meta-analyses were stratified by follow up period (18-24 months, 24-48 months, and 49-72 months). At 18-24 month follow-up the meta-analysis (n=2 studies) found in favour of control for depressive symptoms, but this was driven by improvements in the control in one low quality study, and no change in the intervention groups. At 24-48 month, results were null (n=3 studies). At 49-72 months (n=4 studies) there was a small benefit in depression score favouring the intervention (SMD -0.07, 95% CI -0.15,0.00 P=0.05). Outcomes for child mental health were behavioural outcome measures only | Given the mixed results, it is difficult to recommend this intervention for the purpose of reducing depression |  |
| Housing improvements for health and associated socio‐economic outcomes (Thompson, 2013) | Cochrane review included 33 quantitative studies of housing provision or improvements on health and socioeconomic outcomes, including depression (n=5) | Interventions grouped into: warm and energy efficiency improvements, rehousing and retrofitting interventions, provision of basic housing, rehousing from slums  The studies which measured depression were warm/energy efficiency interventions (n=2), and rehousing/retrofitting interventions (n=3) | Mixed results. Two studies were considered at lower risk of bias, one found that depression decreased in the intervention group (rehouse/retrofit), and one found that it increased in the intervention group (warm home/energy efficiency intervention). The other three studies were at higher risk of bias and one found an improvement (rehouse/retrofit), one found null effects (warm/energy), and one found negative results (rehouse/retrofit) | Given the mixed results, it is difficult to recommend this intervention for the purpose of reducing depression |  |
| The WHO Health Promoting School framework for improving the health and well‐being of students and their academic achievement (Langford, 2014) | Cochrane review of 67 studies of the effect of schools adopting some aspect of the WHO health promoting school framework on health and wellbeing outcomes | Three studies reported outcomes for depression. None of these would clearly meet our definition, despite being described as 'whole of school programmes'. Two focused on emotional wellbeing and/or resilience, one on anti-bullying. All involved health education components, some involved reviewing school policies - but no description of conditions changes | Meta-analysis suggests null effect. Also cite systematic review (Kidger 2012) which considered the effect of school environment changes on the emotional health of adolescents and found limited evidence | n/a |  |
| Workplace interventions to prevent work disability in workers on sick leave (van Vilsteren, 2015) | Cochrane review of 14 RCTs of interventions to increase return to work amongst workers on sick leave | Changes to work design and the organisation (n=13), changes to the workplace and equipment (n=11), changes to work environment (n=9), changes to working conditions (n=6). All were multi-component interventions, and n=12 involved case management  Three studies reported depression/depressive symptoms as an outcome | Pooled MD on depression was ‐0.12 (95% CI ‐0.35 to 0.11). Quality of evidence rated very low | n/a |  |
| **Social Isolation** |  |  |  |  |  |
| **WHO literature** |  |  |  |  |  |
| **Title** | **Summary** | **Methodological Approach** | **Population-level Recommendations** | **Relevant Cited Evidence** | **Notes** |
| Social isolation and loneliness among older people: advocacy brief (2021) | A policy brief which describes the problem, relevance to public policy, and describes the interventional evidence base for reducing social isolation and loneliness. Notes the heavy emphasis on individual-level interventions in the evidence base | Unclear | Appropriate, accessible, affordable transportation - e.g. free bus travel for >60s in the UK [ref 74, 97]  Built environment, housing, and public places design that promotes accessibility and inclusivity [ref 74. 98]  Ensure digital technologies are available, affordable, understandable, and accessible to older people [ref 99]  Promote age-friendly communities [ref 100]  Laws and policies to address discrimination and marginalisation, socioeconomic inequality, and intergenerational cohesion and solidarity, and promote the importance of social capital [refs 1, 74, 101]  Adopt a 'social in all policies' approach [ref 102] | *[1] Cotterell, 2018. Prevention social isolation in older people. Maturitas.* A peer reviewed literature review with systematic search but no double screening or quality assessment. Groups interventions according to the socio-ecological framework. Is not able to recommend any specific interventions at the community or structural levels due to lack of evidence identified, citing only general recommendations from policy reports (in contrast to individual-level interventions for which specific empirical examples with effect sizes are given) [74] *Campaign To End Loneliness, 2020. Promising Approaches Revisited: Effective action on loneliness in later life. C*harity report highlighting case studies, with some empirical before-after type survey evaluations, but exclusively focussed on loneliness. Presents an adapted version of the socioecological framework [97] *Reinhard, 2017. Public transport policy, social engagement and mental health in older age: a quasi-experimental evaluation of free bus passes in England. BMJ JECH* Quasi-experimental study in ELSA, using IV analysis to evaluate the effect of eligibility for an >60 bus pass on depressive symptoms via increase in public transport use, controlling for pension receipts (the most likely confounder). Find that bus passes cause an increase in having regular contact with children (β 0.480, 95% CI 0.208 to 0.752) and friends (β 0.311, 95% CI 0.109 to 0.513), with associated statistically significant reductions in loneliness and depressive symptoms [98] *Domenech-Abella, 2019. Loneliness and depression among older European adults: The role of perceived neighbourhood built environment. Health & Place* Cross-sectional survey of the association between perceived usability of the local built environment with loneliness and depression. Social network size was treated as a confounder. Found a protective association of built environment usability with lower loneliness for non-depressed people only. Not interventional evidence [99] *International Telecommunication Union, 2021. Ageing in a digital world – from vulnerable to valuable.* NGO report with no cited empirical evidence to support a population-level recommendation [100] *WHO, 2007. Global age-friendly cities: a guide* . Implementation guide with case studies - no empirical evidence cited [101] *Visser, 2016. The prevalence and impact of risk factors for ethnic differences in loneliness. Eur J Public Health.* A peer reviewed quantitative analysis identifying associations between risk factors and loneliness (stratified by ethnicity). No empirical interventional evidence [102] *Holt-Lunstad, 2020. Social isolation and health – health policy brief. Health Affairs.* A narrative summary of the topic of social isolation and health. Section on interventions describes policy report recommendations and cites one peer-reviewed review of interventional evidence (Gardiner, 2016) which finds the evidence base to be weak and stops short of recommending any specific public health interventions |  |
| **Cochrane and Campbell Collaboration** | |  |  |  |  |
| Title, Author | Summary | Details of the population-level interventions | Findings | Recommended interventions | Notes |
| Physical environmental designs in residential care to improve quality of life of older people (Harrison, 2022) | Cochrane review of 20 studies (RCTs n=7, CBA n=12, cohort n=1) from HICs of changing the physical environment of residential homes, with or without changes to the functioning of the setting, on quality of life and related measures including depression (n=2) and social engagement (n=2) | The review contains various interventions including re-designs, better lighting, garden refurbishment. But the two studies which included social engagement as an outcome were part of the majority design which compared small-scale more home-like environments to larger homes | One study from the Netherlands reported an increase in social engagement at six months (MD 0.79, 95% CI 0.11 to 1.50). One study from the USA reported no overall change in the level of social engagement at 18 months, but a reduction in the probability of not being socially engaged (OR 0.76, 95% CI 0.62 to 0.94)  Both studies scored poorly on risk of bias assessment, partly due to the non-randomised, non-blinded design (a blinded RCT would have been unfeasible). In the study from the Netherlands, the groups were balanced for social engagement at baseline and reasonably well balanced regarding sociodemographics. The baseline mean MMSE scores were 15.4 and 10.3 in the intervention and control groups respectively, suggesting that the majority (if not all) of the participants had dementia at baseline. In the US study, baseline differences between the group were not reported (though social engagement scores at baseline were comparable). There were 62% dropouts by 18 months, concerns regarding power. 50% had dementia at baseline | Given the high risk of bias in these two studies, and the high number of included participants who had dementia at baseline, we cannot recommend this intervention for primary prevention of dementia based on this evidence |  |
| **Hearing Impairment** |  |  |  |  |  |
| **WHO** |  |  |  |  |  |
| **Title** | **Summary** | **Methodological Approach** | **Population-level Recommendations** | **Relevant Cited Evidence** | **Notes** |
| Global costs of unaddressed hearing loss and cost-effectiveness of interventions (2017) | 2017 WHO report summarising 2 reviews: 1 an estimate of global costs of hearing loss, and 1 a review of cost-effectiveness of interventions to prevent, detect, and treat hearing loss | Available evidence on the cost–effectiveness of interventions for hearing loss was studied in 2016, with a focus on measures aimed at preventing hearing loss, identifying it early, and providing suitable interventions, such as hearing aids, to those requiring them. Although over 500 references were identified, only 36 were found to fit the inclusion criteria as they reported original research findings | None, all recommendations were for clinical interventions:  Just two articles relating to strategies for prevention of hearing loss were available. Baltussen & Smith (2009) looked at the cost–effectiveness of selected interventions for prevention of hearing loss in Africa and Asia; they suggest that passive screening for identification and treatment of otitis media is the most efficient preventive measure in these regions. In Australia, 37% of hearing loss is the result of exposure to excessive noise, which is preventable (Access Economics, 2006). Suitable interventions to raise awareness of, and develop policy for, reduction of noise-induced hearing loss can be a cost-effective option for prevention | *Baltussen R, Smith A (2009). Cost-effectiveness of selected interventions for hearing impairment in Africa and Asia: a mathematical modelling approach. Int J Audiol. 48:144–58.* A mathematical modelling study which estimates the effect of these clinical interventions (screening, treating OTM, and treating meningitis) on hearing loss. Inputs come from empirical data, existing screening programme evaluations, Cochrane reviews of clinical effectiveness *Access Economics (2006). Listen Hear! The economic impact and cost of hearing loss in Australia: a report. Melbourne: Cooperative Research Centre for Cochlear Implant and Hearing Aid Innovation.* In turn cites another study from Australia from 1998 (Wilson) which estimates that 37% of hearing loss has a noise-related component (occupational or recreational) |  |
| Addressing the rising prevalence of hearing loss (2018) | This report aims to highlight the changing profile of global hearing loss over the next century, and summarize the actions required to prevent hearing loss where possible, and mitigate its adverse impact | No methodology is reported for the curation of evidence regarding effectiveness of intervention strategies | Occupational Noise The risk of hearing loss due to occupational exposure is substantial. Implementation of hearing conservation programmes in occupational settings will decrease this risk. Reduction in noise levels, improved regulations and use of protective equipment are effective strategies to mitigate the occurrence of occupational hearing loss (Jh et al., 2012; Lie e.t al., 2016). Hearing conservation programmes were implemented in many countries in Europe at the turn of the millennium. France, Italy, the United Kingdom and the Czech Republic have all reported a decline in the incidence of NIHL in recent years. An example of this is in France, where the occurrence of physician-reported NIHL dropped by 17% between 2007 and 2012 (Stocks et al., 2015). Recreational Noise Unsafe listening practices have been identified as major contributors to hearing loss by the Scientific Committee on Emerging and Newly Identified Health Risks in 2008. They indicated that 5-10% of listeners are likely to develop hearing loss in the future due to their personal preferences of volume levels and duration of listening (SCENIHR, 2008). Another study found that more than 40 million adult Americans between the ages of 20 and 69 are affected by non-occupational NIHL (Centre for Disease Control and Prevention, 2017). Regulation of sound exposure from recreational sources will decrease its risk of hearing loss. This could possibly be achieved by: (i) Implementing global standards for safe listening levels in personal audio devices (ii) Putting in place a regulatory framework for the noise levels at recreational venues, such as restaurants, bars, concerts and sporting events Hearing Aids The assistance provided by hearing devices can substantially enhance the lives of those affected by hearing loss. It must be a priority that high quality, affordable hearing devices are made available to all those who need them. The high cost of devices and their limited availability poses a challenge that must be addressed through innovative designs and delivery models (Wilson et al., 2017). It is also essential that all related services such as auditory rehabilitation and maintenance of hearing devices are available and easily accessible, to ensure that people can maximise the benefit of their use (Carroll et al., 2017).  **Clinical interventions:** Vaccination against infection Implementing an improved vaccination program with widespread coverage will reduce some of the easily preventable infections that can cause hearing loss. Antenatal rubella and meningitis are two such infections that commonly lead to deafness. Vaccine programs have already been successful in reducing their occurrence in some regions, with subsequent prevention of a portion of hearing loss (Cheffins et al., 1998; McIntyre et al., 2012). Hearing loss is the most frequent long-term complication of pneumococcal meningitis, affecting up to 40% of survivors (Klein et al., 2003). It causes profound hearing loss that is mostly permanent. In 2010, the Ministry of Health in Brazil introduced a new vaccine (PCV-10) into the childhood vaccination schedule. Following the implementation of PCV-10, the incidence of meningitis halved over the next five years, with a resultant decline in hearing loss associated with meningitis infections (Grando et al., 2015). Treatment of infection Chronic ear infections are a frequent complication of untreated acute ear infections. By identifying and treating acute otitis media in its early stages, hearing loss caused by chronic otitis media can be avoided (Bluestone, 1998). Improved sanitation and personal hygiene will also reduce the occurrence of chronic ear infections (WHO, 2004). For those who have contracted a chronic ear infection, timely and effective treatment can decrease the risk of hearing loss development. Adequate provision of both surgical and medical management options will improve the outcomes in those affected (WHO, 2004). | Occupational Noise *Jh, V. et al. (2012) ‘Interventions to prevent occupational noise-induced hearing loss.* This is the previous version of the Tikka Cochrane review *Lie, A. et al. (2016) ‘Occupational noise exposure and hearing: a systematic review’, International Archives of Occupational and Environmental Health.* A review of the prevalence of occupational noise exposure-related hearing loss. No interventional evidence reviewed *Stocks, S. J. et al. (2015) ‘Trends in incidence of occupational asthma, contact dermatitis, noise-induced hearing loss, carpal tunnel syndrome and upper limb musculoskeletal disorders in European countries from 2000 to 2012’, Occupational and Environmental Medicine.* Evidence of a change in prevalence, no interventional evidence presented Recreational Noise *Scenihr (2008) ‘Potential health risks of exposure to noise from personal music players and mobile phones including a music playing function’.* Report from the European commission's Scientific Committee on Emerging and Newly Identified Health Risks. Extrapolating from what is known on noise-induced hearing loss (from occupational studies), estimate that 5-10% of users are at risk, however, finds that existing studies are very heterogeneous and overall do not find this to be the case - ultimately calls for more research *Centre for Disease Control and Prevention (2017) Too Loud ! For Too Long ! Loud noises damage hearing.* Webpage for the public, no interventional evidence presented Hearing Aids *Wilson, B. S. et al. (2017) ‘Global hearing health care: new findings and perspectives’, The Lancet.* A non-systematic review of evidence. Present primary, secondary and tertiary prevention options in a table but no references provided for recommended interventions (which include provision of hearing aids). No interventional evidence described directly *Carroll, Y. I. et al. (2017) ‘Vital Signs: Noise-Induced Hearing Loss Among Adults — United States 2011– 2012’, MMWR. Morbidity and Mortality Weekly Report.* CDC analysis of NHANES data, estimating the prevalence of audiometric notches, indicative of noise-induced hearing loss | Supports the Cochrane review evidence on occupational noise  Based on the available evidence at the time of writing (2018), is only able to suggest interventions for recreational noise that logically could be effective, rather than any supported by empirical interventional evidence  There is no interventional evidence to support specific programmes for increasing the availability of hearing aids |
| Occupational noise: assessing the burden of disease from work-related hearing impairment at national and local levels (2004) | The present guide describes how to quantify the burden of disease associated with hearing impairment from occupational noise | n/a | n/a | n/a |  |
| Evidence profile: hearing loss. ICOPE guideline (2017) | Scoping review on whether case finding and provision of hearing aids produces benefits or harms for people over 60 with hearing loss | Systematic literature search identified 1 systematic review (the Barker SR described below) and 3 US-based RCTs of screening +/- hearing corrective devices provision in veteran healthcare associations. Evidence was quality assessed and synthesised in meta-analysis | The authors determine that the evidence is limited and low quality, but that it does suggest that case-finding produces a small increase in hearing aid use, and case finding with provision of hearing aids produces a considerable increase in hearing aid use, and associated benefits to hearing-related quality of life, depression, and social functioning. No studies measured harms, however the authors note there is not likely to be significant harms due to confirmatory testing, and the ultimate intervention, being non-invasive | Mulrow C et al., 1990. Quality-of-life changes and hearing impairment: a randomized trial. Ann Intern Med  Yueh B et al., 2001. Randomized trial of amplification strategies. Arch Otolaryngol Head Neck Surg  Yueh B et al., 2010. Long-term effectiveness of screening for hearing loss: the screening for auditory impairment-which hearing assessment test (SAI-WHAT) randomized trial. J Am Geriatr Soc | This would be a low confidence recommendation, but screening interventions are out of scope as clinical interventions - there is a broader point around the ease of access to, and the cost of, hearing corrective devices |
| **Cochrane** |  |  |  |  |  |
| **Title, Author** | **Summary** | **Details of the population-level interventions** | **Findings** | **Recommended interventions** | **Notes** |
| Interventions to prevent occupational noise‐induced hearing loss (Tikka, 2017) | Cochrane review of 29 studies of occupational interventions to reduce noise exposure and/or hearing loss | Policy change in the US in 2000 which moved from relatively lax to stricter legislation on noise exposure in coal mines, including regular monitoring, primacy of engineering and administrative noise controls, lowering of the action level to 85dBA, above which hearing conservation programmes (which include provision of hearing protection) were mandatory (n=1)  Provision, and mandated use, of free hearing protection equipment with/without education and/or personal noise detection devices (n=11)  Case study examples of interventions to reduce the noisiness of equipment by replacing or retrofitting it, or by segregating noise sources (n=12 B/A studies of 107 examples) | **Noise exposure reductions** - Policy change: (analysis partially conducted by Cochrane authors) immediate effect of a 27.7 percentage points reduction in the median noise dose level (95% confidence interval (CI) −36.10 to −19.30 percentage points) compared to that predicted by extrapolation of the pre‐intervention slope. Given a predicted post‐intervention level of 58.7 PEL dose and a measured level of 31 PEL dose, this means a change from 86.1 dB(A) to 81.6 dB(A) or a 4.5 dB(A) decrease. Robust to moving the intervention date to the announcement of the policy a year earlier. - In general hearing protection devices reduced noise exposure on average by about 20 dB(A) (using sufficiently homogeneous data for comparison from one RCT and three CBAs).  - Case studies demonstrated immediate noise reductions between 11.1 to 19.7 dB(A), but lacked control groups or long-term follow up  **Hearing loss** - No data from the policy change study.  - A meta-analysis of 3 studies comparing well-implemented hearing conservation programmes to less well implemented programmes (based on residual noise levels), each with over 5 years follow up, found a reduction in the risk of sustaining a standard threshold shift at 4Hz (OR 0.40 (95% CI 0.23 to 0.69)) - A hearing loss prevention programme required those workers at a US aluminium factory found to have hearing impairment on yearly audiometric screening to wear ear protection with a built-in noise detector. Across 4 years of follow up, this reduced yearly rate of decline compared to matched controls from other factories −1.35 dB decline per year (95% CI −2.09 to −0.61), after adjusting for sociodemographics and baseline hearing  - A cohort of apprentice welders and fitters at a steelworks in Austria, who were assessed by audiographer at commencement of work and every 3-5 years thereafter, who self-reported usage of hearing protection, reported that across 2-23 years of follow up, increasing the frequency of use of hearing protectors when in noise areas (as required by law) by 10% (from around 80 to 90% usage) decreases NIHL by about 3–5 dB - Studies comparing workers in a hearing protection programme to non-exposed workers found mixed results  Study risk of bias was generally considered to be high, because of non-randomised, retrospective designs, many studies using overall noise exposure within the worksite as a proxy for individual risk exposure, and the potential for residual confounding | Policies to reduce noise exposure through environmental interventions such as changing, retrofitting, or segregating noisy equipment. In addition, and especially where this is not possible, noisy worksites should provide and mandate the use of adequate hearing protection, with regular audiometric monitoring | Education/training only interventions did not work |
| Interventions to promote the wearing of hearing protection (El Dib, 2012) | Cochrane review of 7 studies of interventions to promote the use of hearing protection devices amongst people regularly exposed to loud noises | Multi-component intervention for agricultural students in Wisconsin, providing free hearing protection devices in addition to noise monitoring, education and training, and serial hearing assessments (n=1 cluster RCT intervention, 2 evaluation studies)   The other interventions were training/education/motivation-based only | The % of agricultural students reporting the use of hearing protection "at least sometimes" in noisy areas in the intervention group increased from 23% at baseline to 88% at 4 year follow up, compared to a change of 24% to 45% in the controls (RR 1.94; 95% CI 1.72 to 2.20, P < 0.00001 - completer analysis only, but robust to ITT analysis). In a survey, 94% of students reported the provision free equipment was a driver of adherence, more than any other factor (e.g. yearly hearing test 90%, classroom instruction 66%). However, in a separate audiometric evaluation of this intervention, no change in hearing impairment was observed, but the authors conclude that 3 years probably provides insufficient follow-up time for this analysis | Supportive of the findings of the Tikka review | Note there was a 2013 update but this was withdrawn due to analysis error, with no capacity to correct and re-publish this version  N.B. the Wisconsin RCT was also covered by the Tikka review |
| Interventions to improve hearing aid use in adult auditory rehabilitation (Barker, 2016) | Cochrane review of 37 studies of interventions to promote the use of hearing aids for adults with acquired hearing impairment | None - despite attempts to identify studies evaluating the effect of community-based or hospital-system-based availability interventions, only self-management interventions were identified | Mixed results, mostly null or small benefits of unclear clinical significance | n/a |  |
| Hearing aids for mild to moderate hearing loss in adults (Ferguson 2017) | Cochrane review of 5 RCTs on the effect of hearing aids for hearing loss | None - the RCTs simply involved providing participants with professionally fitted hearing aids, or waiting list controls. Measured outcomes were hearing-related QoL, and listening ability | Meta-analyses showed significant benefits for all outcomes | n/a | This would provide supportive evidence for an intervention to improve population access to hearing aids |
| **Education** |  |  |  |  |  |
| **Cochrane** |  |  |  |  |  |
| **Title, Author** | **Summary** | **Details of the population-level interventions** | **Findings** | **Recommended interventions** | **Notes** |
| Later school start times for supporting the education, health, and well‐being of high school students (Marx, 2017) | Cochrane review of 11 studies of later school start times on academic outcomes as measured by the grade point average (either comparing the change in the intervention group to the control (n=3), or comparing period 1 classes to period 3 (n=1)), some studies also reported on absenteeism | Delayed morning (one study also considered the effect of delaying the afternoon session) start time for middle- or secondary- school | Mixed. One study reported worse academic performance, one reported better, one reported non-significant changes, and one did not report quantitative results. Of those studies reporting on absenteeism, some reported improvements, whilst others reported no change | n/a |  |
| Financial benefits for child health and well‐being in low income or socially disadvantaged families in developed world countries (Lucas, 2008) | Cochrane review of 9 studies involving provision of financial support (e.g. through welfare reform) on child health | Studies of the effect of welfare reforms (n=8), dollar-a-day teenage pregnancy reduction programme (n=1). All studies from the US | Two studies reported absenteeism outcomes, with one finding a significant benefit, and one null. No significant effects were seen in 2 studies for proportion of students suspended or expelled, or in one study on school drop out | n/a |  |
| Physical activity, diet and other behavioural interventions for improving cognition and school achievement in children and adolescents with obesity or overweight (Martin, 2018) | Cochrane review of 18 studies of the effect of healthy lifestyle interventions for overweight children on cognition and school achievement | All interventions were multi-component, and all were predominantly individual-level focused. Population-level components included: curriculum changes to increase the amount of physical activity built into the school day (n=15), improvements to the school play equipment (n=1), provision of healthier foods (n=2) | No measures of school attainment or attendance reported. Some evidence of improved performance in interventions which included provision of healthier foods, but null effects for physical activity-based interventions | n/a |  |
| The WHO Health Promoting School framework for improving the health and well‐being of students and their academic achievement (Langford, 2014) | Cochrane review of 67 studies of the effect of schools adopting some aspect of the WHO health promoting school framework on health and wellbeing outcomes, including educational achievement | Only two interventions measured an outcome of interest (absenteeism) and they were both health education campaigns to increase handwashing | Not relevant due to intervention design, but a reduction in absenteeism was observed for the handwashing interventions | n/a |  |
| Slum upgrading strategies involving physical environment and infrastructure interventions and their effects on health and socio‐economic outcomes (Turley, 2013) | Cochrane review of 5 studies of slum upgrades on health and socioeconomic outcomes - including education (n=1) | Multi-component interventions to improve the physical environment, such as water supply and sanitation, electricity supply, cement flooring | Null effects in one study on school enrolment, absenteeism, and household literacy | n/a |  |
| Unconditional cash transfers for reducing poverty and vulnerabilities: effect on use of health services and health outcomes in low‐ and middle‐income countries (Pega, 2022) | Cochrane review of 34 studies of unconditional cash transfers for poverty alleviation in LMICs on various health and SES outcomes, including education (n=8) | 8 cluster RCTs from Africa (Malawi n=2, Kenya n=2, Zambia n=2, Lesotho n=1, Zimbabwe n=1), running for 2 years (n=6), 16 months (n=1), 3 years (n=1). Providing unconditional cash transfers to poor households (n=3), poor households with children (n=2), children (n=1), women aged 13-22 (n=1), children and adults (n=1). One CBA study, not included in the meta-analysis, from Indonesia, 1 year long, targeting poor households | Meta-analysis of effect showed a small benefit, RR 1.06 (1.04, 1.09) on school attendance either measured by "current attendance" at time of survey, or "has not missed a day of school in the last month". The included studies were very heterogeneous with which age groups they included (ranging from 3-5 years to 15-17 years), however, the Cochrane authors felt this was appropriate for meta-analysis. In absolute terms, assuming a mean in the control group of 676 attenders per 1000 children (i.e. the median across the six studies with such data), after receiving the unconditional cash transfer, an estimated 717 per 1000 children (95% CI 703 to 737) attended school - which the Cochrane authors judged to be clinically meaningful. The results of the CBA study are not reported, but reviewing the primary study, they report a null effect on school dropout rate. Based on the quality and consistency of evidence, the Cochrane authors considered this to be a moderate confidence recommendation | Provide unconditional cash transfers to increase school attendance in poor households in LMICs, RR 1.06 (1.04, 1.09) |  |
| Community‐based supplementary feeding for food insecure, vulnerable and malnourished populations – an overview of systematic reviews (Visser, 2018) | Cochrane umbrella review of 8 systematic reviews of food security interventions on various outcomes (n=1 for education) | Only 1 review (Kristjansson 2007) included evidence on change in school attendance, see below | See below | n/a |  |
| School feeding for improving the physical and psychosocial health of disadvantaged students (Kristjansson, 2008) | Cochrane review of 18 studies providing free food at school for disadvantaged students on their health and psychosocial outcomes | 6 studies measured school attendance, and provided breakfast (n=3), lunch (n=2), and breakfast, lunch and snacks (n=1), for between 5 weeks and 3 years. Studies were conducted in USA (n=2), Peru, Jamaica, India, and Canada, using cluster RCT design (n=2), and CBA studies (n=4). Children were aged 5-9 (n=5) and 11-12 (n=1) | 5/6 studies reported increases in school attendance. The effect sizes for the cluster RCTs were: 3.4% and 2.3% more days attended in the 8 month and 5 week study periods in Jamaica and Peru, respectively. | Provide free school meals, such as breakfast and/or lunch, for children who would not otherwise have access to sufficient food, to increase school attendance by 2.3-3.4% |  |
| Housing improvements for health and associated socio‐economic outcomes (Thomson, 2013) | Cochrane review of 33 quantitative studies of housing provision or improvements on health and socioeconomic outcomes | Interventions grouped into: warm and energy efficiency improvements, rehousing and retrofitting interventions, provision of basic housing, rehousing from slums  The studies which measured education were warm/energy efficiency interventions (n=4), and rehousing/retrofitting interventions (n=2) | 3/3 powered, high-quality, studies of warmth/energy efficiency interventions reported statistically significant reductions in school absenteeism. Two RCTs from New Zealand in low income areas reported statistically significant reductions in absenteeism for retrofitting insulation: 0.49, 0.31 to 0.80; P=0.004 (specifics of outcome measure unclear), and installation of an efficient, non-polluting home heater 1.80 fewer days off school (0.11 to 3.13, P=0.04) during the winter term (100 school days) using statutory school records on absence - with parent self-report suggesting reduced asthma illnesses may be a mechanism for this. A natural experiment study in Cornwall examined the effects of non-recurrent NHS underspend funding on housing improvements e.g. gas central heating installation in damp homes in 1994. As assessed by questionnaire, amongst the 45 children aged 5+ in the households, number of days missed per 100 school days due to asthma were higher before vs. after the intervention (rate ratio 7.27 (3.32-11.21, P<0.0001), with no change in the non-asthma related school absences   One rehousing/retrofitting study found null effects on days of school in past month. One retrospective health equity audit of rehousing support for Roma groups in Hungary did report an increase in years of education (67% after vs. 60% before, for having 8+ years of schooling when reaching adulthood), but this study scored poorly on risk of bias assessment | Improvements to the energy efficiency, or warmth, of poor-quality housing, to reduce school absenteeism - example effect size: 1.80 fewer days off school (0.11 to 3.13, P=0.04) |  |

| Supplementary Table 3B – Extraction of further searches for depression literature | | | |
| --- | --- | --- | --- |
| **TRIP** |  |  |  |
| **Author/Year** | **Title** | **Summary** | **Possible intervention for inclusion?** |
| Shah, 2021 | National or population level interventions addressing the social determinants of mental health – an umbrella review | Umbrella review aiming to identify the best available evidence for national or population level policies or interventions that improve population mental health and wellbeing by addressing the social determinants of mental health. Most identified reviews considered observational evidence only and scored poorly on risk of bias assessment. Higher quality reviews considering interventional evidence were either Cochrane reviews we've already considered, or found insufficient evidence to make strong recommendations | No - and confirms a lack of evidence exists despite attempts to find it |
| Simpson, 2021 | Effects of social security policy reforms on mental health and inequalities: A systematic review of observational studies in high-income countries | SR of 38 natural experiment studies of the effect of social security policy reforms on mental health outcomes (depression n=13). Very mixed results, half of studies found an effect, and half found null results. No clear pattern of results by expansionary vs. contractionary policies; or for children vs. adult benefits. Stronger signal for generally improved mental wellbeing, so unclear whether misclassification bias and power could explain null results for depression. All data from HICs | Represents a signal that welfare support can improve mental health (as one would expect) but not a specific intervention we can recommend. Suggest softer recommendation with Reeves studies (see below) as examples |
| Nunez-Gonzalez, 2020 | Overview of “Systematic Reviews” of the Built Environment's Effects on Mental Health | Umbrella review of 11 reviews on built environment and mental health. Only 2 included reviews measure depression specifically as an outcome. Of these only 1 considers interventional evidence - a Cochrane review on slum improvements (we extracted only for education), which identified one controlled retrospective study from Mexico which scored well on risk of bias assessment and reported fewer symptoms of maternal depression post-intervention in intervention areas where cement floors were installed in slum houses (CES‐D scale MD ‐2.37; 95% CI‐3.46 to ‐7.02; P < 0.001) | The intervention here is housing improvement (albeit in a very impoverished setting) so should be seen in the context of the 2013 Cochrane review by Thompson, which found mixed results. Again, this is probably a signal to be included as a softer recommendation |
| Moore, 2018 | The effects of changes to the built environment on the mental health and well-being of adults: Systematic review | SR of 14 urban regeneration, green space, or transport interventions on mental health. Depression only considered in single questions within national surveys, that combine feeling depressed or anxious, so not relevant here | n/a |
| Nagy, 2017 | Social interventions: An effective approach to reduce adult depression? | SR of 24 intervention studies measuring the effect of 'social interventions' (interpersonal interventions that aimed to strengthen bons between individuals and their environment) on adult depression. Reviewing the intervention designs in detail, none would meet our definition (e.g. group therapy, exercise classes, psychoeducation, peer support) | n/a |
| **Google Scholar** |  |  |  |
| Reavley, 2010 | Prevention and early intervention to improve mental health in higher education students: a review | Systematic search for evidence relating to interventions for the prevention of/early intervention for depression amongst higher education students, with an explicit attempt to identify individual- and population-level evidence. Only identified social marketing-type intervention for population-level (information booklets and posters) | No - and confirms a lack of evidence exists despite attempts to find it |
| Reeves, 2016 | Reductions in the United Kingdom's Government Housing Benefit and Symptoms of Depression in Low-Income Households | Included in the Simpson review | Consider in the context of the broader Simpson review |
| Reeves, 2016 | Introduction of a National Minimum Wage Reduced Depressive Symptoms in Low-Wage Workers: A Quasi-Natural Experiment in the UK | Natural experiment study of introduction of minimum wage in UK, comparing people who received additional income to those in the next 10% or at private companies who did not implement the minimum wage. Used panel data, 1 year apart. Compared to controls, found that depressive symptoms ("have you recently been feeling unhappy or depressed" yes/no), reduced differences in differences (mean) −0.14, SE = 0.08, p = 0.045). Effect size for whole of the GHQ (12-item mental health/wellbeing score) was equivalent to 0.39 of an SD, which the authors state is comparable to the effect of anti-depressants on depression. No difference observed for hearing loss, cigarettes smoked, or blood pressure (control conditions) | Not a reliable outcome measure, but supports a softer recommendation of Simpson review (above) of social security/welfare-type interventions |
| Caldwell, 2019 | School-based interventions to prevent anxiety and depression in children and young people: a systematic review and network meta-analysis | Systematic search for evidence relating to interventions for the prevention of depression in schools, with an explicit attempt to identify individual- and population-level evidence. Unable to recommend any population-level interventions based on evidence available | No - and confirms a lack of evidence exists despite attempts to find it |
| Hunter, 2019 | Environmental, health, wellbeing, social and equity effects of urban green space interventions: A meta-narrative evidence synthesis | SR of 38 intervention studies of urban green interventions on health (depressive symptoms n=1). A Dutch study, leveraging serial panel data and comparing adolescents in intervention and control areas, noted a small reduction in depressive symptoms following interventions such as park refurbishment and improving municipal paths, drainage, and gardens | Insufficient on its own |
| Wenelboe-Nelson, 2019 | A Scoping Review Mapping Research on Green Space and Associated Mental Health Benefits | Scoping review of 263 studies of green space interventions and mental health. 19 studies measured depressive symptoms by questionnaire, 1 study measured clinical depression. Vast majority of studies non-interventional. Of the studies described in any detail which measured depression, all were nature-based psychotherapy (e.g. forest walks). Overall commented that the evidence base lacks robust interventional designs | No |
| WHO, 2017 | Urban green space interventions and health: a review of impacts and effectiveness | N.B. underpinning evidence review is by Ruth Hunter (see 2019 paper above). Evidence review of 38 green space interventions on health/environmental outcomes. Identified the same single study with depression as an outcome as Hunter 2019. Commented on the generally low-quality of the evidence base | No |
| Kondo, 2018 | Urban Green Space and Its Impact on Human Health | Two studies measured an effect on depression (or depressive symptoms) and met our definition. Both involved greening-type urban space improvement interventions. One (Gubbels 2016) was included by Hunter 2019 above. The other, though underpowered, reported no effect |  |
| **Papers from experts/professional networks** | |  |  |
| Rose, 2023 | The mental health and wellbeing impact of a Community Wealth Building programme in England: a difference-in-differences study | A difference in difference comparison of a community wealth building intervention in Preston, UK to comparable areas. Community wealth building was initiated in 2013, and involved working with local anchor institutions to address economic inequalities through investment, procurement, paying a living wage etc. NHS data up to 2019 showed that antidepressant prescriptions and depression incidence increased less quickly compared to controls. Antidepressants absolute effect: ADQ -1.25 (-1.78, -0.72) p<0.001; relative reduction 3%; cost-saving £108,000. Depression incidence absolute effect per 100 population -2.44 (-4.46, -0.42) p=0.018; relative reduction 2%. Greatest reductions in poorest LSOAs. No change in mental health-related admissions. Compared to synthetic control, employment, wages, and life satisfaction all improved significantly - which the authors suggest as possible mechanisms (not possible to compare to other areas due to data availability). Robust to various sensitivity analyses | Impressive study, but only in one area. One anchor institution involved, the university, announced a £200 million pound investment in its campus in 2015 which the authors acknowledge could be a driver of results, rather than the community wealth building specifically. Note, anti-depressant prescribing and depression incidence increased in Preston and control areas during study (less in Preston) - so although methods theoretically robust to this, does mean that other trends were in play during the study. To discuss |
| Baskin, 2020 | Community-centred interventions for improving public mental health among adults from ethnic minority populations in the UK: a scoping review | SR of 7 community-centred interventions to improve mental health amongst ethnic minority groups in the UK. Only 1 of the studies meets our intervention definition (free gym membership), but this study measures only physical activity rates and perceived health benefits | n/a |
| Walsh, 2022 | A systematic review of the cost-effectiveness of community and population interventions to reduce the modifiable risk factors for dementia | SR of economic studies of population-level interventions for all 12 (Lancet commission 2020) modifiable risk factors for dementia. Did not identify any studies for depression | No - and confirms a lack of evidence exists despite attempts to find it |

| **Supplementary Table 3C – Extraction of further searches for social isolation literature** | | |  |
| --- | --- | --- | --- |
| **TRIP** |  |  |  |
| **Author/Year** | **Title** | **Summary** | **Possible intervention for inclusion?** |
| Moore, 2018 | The effects of changes to the built environment on the mental health and well-being of adults: Systematic review | SR of 14 urban regeneration, green space, or transport interventions on mental health. 3 studies measured something related to social isolation. Results were almost all null. A UK study of urban regeneration found no effect on feeling part of the community, perception of friendliness of locals, and social relations problems. A US study found no effect of a green space improvement programme on perceived friendliness of the local neighbourhood. A Norwegian study found that turning roads into pedestrianised 'street parks' increased acts of neighbourly support, but increased neighbour annoyance, and had no effect on neighbourly attachment, or social ties | No |
| Tcymbal, 2022 | Interventions simultaneously promoting social participation and physical activity in community living older adults: A systematic review | SR of 46 studies of interventions for community-dwelling older adults measuring both social participation and physical activity as an outcome. Only one study involved a population-level intervention (co-produced urban improvements e.g. construction of walkways and installation of benches in a relatively deprived part of Copenhagen) but evaluation was via qualitative interviews only - which suggested the intervention had succeeded in increasing levels of social participation amongst community dwelling older people | No |
| **Google Scholar** |  |  |  |
| Cotterell, 2018 | Preventing social isolation in older people | Review already identified by WHO extraction - lack of population-level evidence | No - and confirms a lack of evidence exists despite attempts to find it |
| Osborn, 2021 | Interventions to address loneliness and social isolation in young people: A systematic review of the evidence on acceptability and effectiveness | SR of 16 interventions to reduce loneliness or social isolation. No population-level interventions, and only one study measured social isolation | No - and confirms a lack of evidence exists despite attempts to find it |
| Day, 2020 | A public health approach to social isolation in the elderly | Perspective piece on the role of health visitors and district nurses in alleviating loneliness for older people, including comments on their role as social connectors, befriending, the effects of the pandemic, and digital inclusion. No empirical evidence presented | No |
| Orlando, 2021 | The Effectiveness of Intervening on Social Isolation to Reduce Mortality during Heat Waves in Aged Population: A Retrospective Ecological Study | A retrospective study of an individual-level, clinical intervention to support people who are socially isolated, and educate them about heat waves, demonstrating a reduction in heat wave-related mortality as a result | No |
| Nishimi, 2022 | Master-planned communities in the United States as novel contexts for individual and population-level research | Perspective piece considering the opportunities and challenges related to new housing developments and health effects. No empirical data presented | No |
| Hodge, 2021 | Legal and Policy Interventions to Address Social Isolation | A perspective piece considering the potential ethical/legal aspects of hypothesised interventions | No |
| Cattan, 2005 | Preventing social isolation and loneliness among older people: a systematic review of health promotion interventions | SR of 30 studies of interventions to reduce social isolation or loneliness in older people. No interventions meet our definition of a population-level intervention | No |
| Sandu, 2019 | Addressing loneliness and social isolation among older people in Europe | Policy brief for the European Centre for Social Welfare Policy and Research. Describes the issue, provides some case study examples of programmes designed to tackle loneliness/social isolation for older people but no empirical evidence of effectiveness | No |
| Gardiner, 2016 | Interventions to reduce social isolation and loneliness among older people: an integrative review | An attempt to reconcile the fact that several reviews of quantitative evidence of intervention effectiveness for social isolation/loneliness have found mixed and often contradictory results, by integrating different sources of evidence. Included 39 studies (6 RCTs, 21 other quantitative, 10 qualitative, 2 mixed methods). Evidence of mixed quality, and only trials of psychotherapy were considered low risk of bias. Thematic analysis identified 6 types of intervention (social facilitation interventions, psychological therapies, health and social care provision, animal interventions, befriending interventions and leisure/skill development) none of which would fully meet our definition of a population-level intervention | No - and confirms a lack of evidence exists despite attempts to find it |
| Dickens, 2011 | Interventions targeting social isolation in older people: a systematic review | SR of 32 intervention studies for social isolation or loneliness in older people. No studies fully met our definition of a population-level intervention. The quality of the evidence base was found to be moderate to poor | No - and confirms a lack of evidence exists despite attempts to find it |
| Markzac, 2019 | Preventing social isolation and loneliness among older people | Rapid literature review of interventions to prevent social isolation and loneliness. Describe four levels of possible interventions (individual, group, community (e.g. provision/accessibility of public libraries), technological). But only presents evidence for individual, group, and technological - suggesting none was found for community-level interventions, but this is not explicitly stated | No |
| Courtin, 2015 | Social isolation, loneliness and health in old age: a scoping review | A scoping review that identified 9 intervention studies for loneliness and social isolation, none of which were population-level (mostly befriending or carer support interventions) | No - and confirms a lack of evidence exists despite attempts to find it |
| Fakoya, 2020 | Loneliness and social isolation interventions for older adults: a scoping review of reviews | A scoping umbrella review which identified 33 reviews of interventions for social isolation/loneliness in older adults. None of the reviews described indicated an inclusion of population-level evidence | No - and confirms a lack of evidence exists despite attempts to find it |
| Taylor, 2023 | The state of loneliness and social isolation research: current knowledge and future directions | Editorial stating that we need more research to understand what the best primary prevention interventions would look like for social isolation. No empirical evidence (or references) provided | No |
| McGuire, 2022 | Tackling poverty and social isolation using a smart rural development initiative | Before/after study using regression analysis of repeated survey data to evaluate a rural isolation/poverty initiative (including home improvements, occupational therapy, and transport infrastructure improvements) in Northern Ireland. Not every household in the survey data received each intervention, so households not receiving the intervention acted as controls - but, as these interventions were targeted, not randomly assigned, there is likely bias introduced here. Find that self-reported indicators of social isolation (reporting improvements on any of 6 questions about social disconnectedness, exclusion, loneliness) reduced for the total cohort by 22.5% (aged 40-59), and 14.8% (60+). In those who benefited from the transport intervention (e.g. free passes/improved access) social isolation reduced by 20.7% | This is a relevant intervention, but the quality of evidence is too low to use this study alone to make a recommendation |
| **Papers from experts/professional networks** | |  |  |
| Baskin, 2020 | Community-centred interventions for improving public mental health among adults from ethnic minority populations in the UK: a scoping review | SR of 7 community-centred interventions to improve mental health amongst ethnic minority groups in the UK. Only 1 of the studies meets our intervention definition (free gym membership), but this study measures only physical activity rates and perceived health benefits | n/a |
| Walsh, 2022 | A systematic review of the cost-effectiveness of community and population interventions to reduce the modifiable risk factors for dementia | SR of economic studies of population-level interventions for all 12 (Lancet commission 2020) modifiable risk factors for dementia. Did not identify any studies for social isolation | No - and confirms a lack of evidence exists despite attempts to find it |

| **Supplementary Table 3D – Extraction of further searches for education literature** | | | |
| --- | --- | --- | --- |
| **TRIP** |  |  |  |
| **Author/Year** | **Title** | **Summary** | **Possible intervention for inclusion?** |
| Psaki, 2022 | Policies and interventions to remove gender-related barriers to girls' school participation and learning in low- and middle-income countries: A systematic review of the evidence | Campbell Collaboration SR of 82 studies of interventions to address gender-related barriers to schooling for girls in LMICs. Authors consider there is consistent evidence, judged to be low risk of bias, for interventions against 3 barriers:  (1) Inability to afford tuition and fees - direct evidence 7 studies (free primary schools in Uganda n=3 studies, reporting improvements in primary enrolment, years of education, grade attainment, and primary completion; studies of primary school tuition waivers (e.g. via conditional payments to the school) n=3 from Haiti, Pakistan, Ecuador reporting mixed effects on primary enrolment, and secondary school tuition waivers n=1 from Ghana, reporting benefits to secondary enrolment, completion, and grade attainment), supported by 10 further studies which were multi-component but included free schooling.  (2) Lack of adequate food - two studies reported effects on boys and girls combined. One examined the effects of policies to improve access and quality of schooling in Burkina Faso, using secondary data from surveys and RCTs, reporting significant benefits to primary school enrolment, and average grade attainment across primary and secondary, mainly for in-school feeding programmes and also out of school rations in some cases. Another considered the effect of a national policy to provide free lunches to all primary school children in India and reported significant benefits to primary school enrolment. Ten studies reported effects on girls specifically, with around half reporting benefits and half reporting null effects or non-significant benefits (3) Inability to afford school materials - these results are similar to those for adequate food, with 2 studies reporting benefits when effects are combined for boys and girls, and a more mixed picture for girls alone. However, the evidence quality was more mixed, the interventions were more heterogeneous, and the majority of evidence was from multi-component interventions (e.g. combined uniform provision with support for tuition fees)  Interventions against inadequate access to school, and lack of water/sanitation were found to be consistently effective, but the authors were unable to disentangle the specific effects of these interventions from the multi-component programmes they were part of. Most studies came from sub-Saharan Africa or Asia. Quality was generally high, though some included quasi-experimental studies were at risk of bias - but authors explicitly considered these factors when grading overall confidence in recommendations | Fiscal interventions (including removing school fees, conditional payments to schools, conditional cash transfers to households) to support children in LMICs to attend school, where financial barriers would otherwise exist - see also Patel-Campillo  Free provision of food in-school with/without additional take-home rations, in LMICs where a lack of food would otherwise occur (boys and girls) (N.B. this builds on an existing recommendation derived from an earlier Cochrane review) |
| **Google Scholar** |  |  |  |
| Greenberg, 2017 | Universal Interventions: Fully Exploring Their Impacts and Potential to Produce Population-Level Impacts | A perspective piece on the importance of population-level strategies within school settings (as opposed to individual-level ones). Draws on some evidence but not a formal review, focused on outcomes such as behavioural problems, rather than educational attainment | No |
| Freeman, 2015 | Examining the Impact of Policy and Practice Interventions on High School Dropout and School Completion Rates: A Systematic Review of the Literature | SR of 32 studies of interventional evidence analysing the effect of policy or school-based interventions to reduce dropout rates and increase completion rates in high schools. Mixed results. Most studies reporting a positive effect were multi-component made up of predominantly individual-level interventions. Some signal for smaller class sizes, but insufficient to make a specific recommendation | No |
| Nishimura, 2008 | Impacts of the universal primary education policy on educational attainment and private costs in rural Uganda | This policy was also evaluated by Grogan et al. in 2009, and included through this study in the Psaki review above | Already included (Psaki, 2022) |
| Pressler, 2016 | Increasing Low-income Mothers’ Educational Attainment: Implications for Anti-poverty Programs and Policy | Secondary regression analysis of Head Start programme in Chicago, suggesting that low-income mothers' randomised to the intervention (enhanced pre-school offer for their children) were more likely to have increased their educational attainment, e.g. through college courses, by the time their child reached 5th grade, compared to those randomised to control | No - indirect effect from one study with a relatively small sample size |
| Patel-Campillo, 2022 | Breaking the poverty cycle? Conditional cash transfers and higher education attainment | Natural experiment study of the effect of a conditional cash transfer programme in Peru, using panel data. Cash transfer was equivalent to 22% of the minimum wage, was delivered on a means tested basis to mothers in poor families, and was conditional on children being in school with >85% attendance. Panel data from the Young Lives Survey (n=587 children, of whom 93 were Juntos recipients). Intervention associated with an 11.4% absolute increase in attending university, compared to controls. However, this result was driven exclusively by males, with no effect on females  N.B. published after searches for Pega 2022 review on UCTs (and this is conditional, not unconditional), and after Psaki 2022 review (and this excluded household cash transfers) | Yes - conditional cash transfers |
| Braga, 2011 | Institutional Reforms and Educational Attainment in Europe: A Long Run Perspective | Regression analysis of the effect of educational policy interventions in Europe from the Eurydice database, with attainment from various European surveys. Find that increasing access to early years education, and expanding compulsory education ages (either by lowering the compulsory starting age of education, or increasing the leaving age) are statistically significantly associated with increased educational attainment, and equity benefits. Increasing EY access is supported by references to supporting evidence. Increasing compulsory education is supported by several references to other empirical data (Aakvik 10 - Norway, Brunello 09 - Europe-wide, Meghir 05 - Sweden) which each find increases in attainment associated with later compulsory leaving age, and equity effects. Alterations to financial support for higher education were associated with non-significant increases in attainment. Also reports some technical interventions such as the use of standardised tests were associated with significant effects  N.B. Not peer reviewed but presented to the European Commission Panel Meeting of Economic Policy | Yes - increasing school leavers age. Obvious caveat is that supply needs to be there, and financial barriers overcome, data from HICs only |
| Hollands, 2014 | Cost-Effectiveness Analysis in Practice: Interventions to Improve High School Completion | 5 interventions selected for cost-effectiveness analysis are individual-level interventions | No |
| Wilson, 2011 | Dropout Prevention and Intervention Programs: Effects on School Completion and Dropout among School-aged Children and Youth | SR and MA of dropout prevention interventions. Most are individual-level interventions, or technical education reforms - e.g. provision of alternative schooling options for excluded children | No |
| Lyche, 2010 | A Literature Review on Policies to Prevent Dropout and Early School Leaving | An OECD review of causes and preventative interventions for high school dropout. Identifies 68 policies/interventions from literature searches but presents these in case study format rather than any synthesis from which general recommendations can be extracted. Does provide supportive evidence for the use of conditional cash transfers in the UK (Dearden 2009), but not for channelling of financial resource towards local authorities in deprived areas in France, Germany or Denmark | No - supportive evidence for a CCT recommendation |
| Earle, 2018 | Is free pre-primary education associated with increased primary school completion? A global study | Cross-sectional regression analysis using UNESCO data to analyse the effect of free and compulsory early years provision in 104 countries, controlling for national income and urbanisation. Find that provision of at least 1 year of free and compulsory pre-primary education is associated with a nearly 10 percentage point increase in primary school graduation rates for countries at the median and a 12 percentage point increase in rates for primarily low- and lower-middle-income countries at the lower end of the distribution. Does not investigate the effect of policy implementation, so this represents supportive observational data rather than direct empirical evidence meeting our inclusion criteria | Supports first recommendation from Psaki review (in fact extends it to pre-primary) |
| Cohen, 2012 | Education: A Missed Opportunity for Public Health Intervention | Perspective/review article without search strategy, describing important aspects of educational policy/education interventions at various stages of childhood for education and health outcomes. References Glymour 09 showing effects of compulsory schooling age changes in the US (adds to European evidence above) | No |
| Lehr, 2003 | Moving beyond dropout towards school completion: An integrative review of data-based interventions | SR of 45 studies of interventions to reduce school dropout. No included intervention meets our definition | No |
| **Papers from experts/professional networks** | |  |  |
| Snilstveit, 2015 | Interventions for improving learning outcomes and access to education in low- and middle- income countries: a systematic review | Non-peer reviewed SR for an NGO which identifies 58 studies of interventions to improve education access and outcomes in LMICs. Structure results into clinical interventions to reduce educational effects of ill-health; providing information on future benefits of education; school feeding programmes n=16 - meta-analyses report improved attendance, dropout, enrolment but no data on completion; merit-based scholarships n=11 with null effects on dropout and attendance, but significant benefits for school completion (2 studies), fiscal interventions - reducing school fees (n=4) eliminating fees (n=4) providing uniforms (n=2), meta-analyses report non-significant effects on enrolment, attendance, dropout, and completion; cash transfers (n=50 of which n=47 conditional), meta-analyses report significant effects on enrolment, attendance, dropout, and completion; technical pedagogical interventions; provision of remedial education (n=4) effects on outcomes only; longer school day (n=3) effects on outcomes only; provision of school materials (n=4) no meta-analysis, results from 1 study each show non-significant effects for dropout and completion, and null effects on enrolment and attendance; school construction or improvement (n=7) meta-analysis reports significant benefits for enrolment and attendance, no data on dropout or completion; grouping students by ability; teacher incentives; decentralising authority to school level (n=14) meta-analyses report null effect on enrolment, non-significant for completion and dropout; community-based school monitoring interventions (n=11), meta-analyses report significant benefit for enrolment and completion, non-significant for attendance, null for dropout; public/private partnerships (n=13), meta-analyses report significant benefit on enrolment, and non-significant for completion; multi-level interventions (n=12), meta-analyses report significant benefit on completion, non-significant enrolment, attendance, dropout | No quality assessment of included studies, and not peer reviewed. Provides further supportive evidence for school feeding programmes and cash transfers (mainly conditional). Less supportive of school fee interventions and school materials interventions, but includes few studies in this regard |
| Akyeampong, 2023 | Cost-Effective Approaches to Improve Global Learning | Report from Global Education Evidence Advisory Panel to the FCDO, World Bank, UNICEF, and USAID convened to provide policy-focused recommendations to support decision-making on education investments in LMICs. Presented in Great Buys/Good Buys/Promising but Limited Evidence format. Underpinned by what reads like a comprehensive but non-systematic review of literature - methods and results not fully described. Mostly outcomes are not specified beyond "improving global learning", occasionally specified as enrolment or test scores. Great Buys recommended are providing information on benefits of education, structured pedagogy (e.g. providing lesson plans, learning materials, teacher training). Good Buys recommended are providing parent-directed EY programmes 0-36 months, and pre-primary education (3-5 yrs), reducing travel times to schools, giving merit-based scholarships to disadvantaged CYP, and administering school-based mass deworming. Promising interventions were software, community-hired staff, mass school based treatments, mobile phones, and safeguarding. Results mostly written in case study fashion | No |
| Walsh, 2022 | A systematic review of the cost-effectiveness of community and population interventions to reduce the modifiable risk factors for dementia | SR of economic studies of population-level interventions for all 12 (Lancet commission 2020) modifiable risk factors for dementia. 10 studies were included which considered education interventions, of which 8 meet our definition of a population-level intervention and measure an outcome related to level of education achieved, or educational attainment. Interventions were heterogeneous, but 4 reduced financial barriers, 2 included education system reform, 1 provided free hot lunches, and 1 provided free pre-school care and support to parents in deprived areas. 7/8 studies reported significant benefits, on years of secondary school completed or high school completion (n=6), and/or higher education enrolment/ matriculation (n=4). One study (provision of free hot lunches only in Senegal) reported non-significant effects on primary school dropout rates, and secondary school enrolment  Evidence from both HICs and LMICs  N.B. no assessment for publication bias in this systematic review (not possible due to heterogeneity of literature examined) | Yes - consistent evidence for the reduction of financial barriers increasing educational attainment  Some evidence for education reform and community-centre support in early life, but not enough for specific recommendation |
| Sakaue, 2018 | Informal fee charge and school choice under a free primary education policy: Panel data evidence from rural Uganda | Several studies included by the Psaki review examine the effects of Uganda's free primary school policy. This study reports that, in spite of legislation prohibiting government-funded schools taking money from parents, the practice of requesting 'informal' fees is common in rural communities. In addition, several fee-paying private schools have opened. This study uses panel data to regress the size of school fees (formal or informal) on likelihood of attendance for children from poorer households - finding that increased cost is an equity barrier | No but useful context |

| **Supplementary Table 4 – Extraction of existing non-communicable disease prevention frameworks** | | | | |
| --- | --- | --- | --- | --- |
| **Author/Year/Title** | **Summary** | **Methods for framework development** | **Framework** | **Notes on applicability to the evidence we have collected** |
| Carey, 2015 Systems change for the social determinants of health | An analysis of recommendations from major social determinants of health reports using the concept of ‘system leverage points’ | Review across several social determinants of health reports, e.g. the Marmot report | 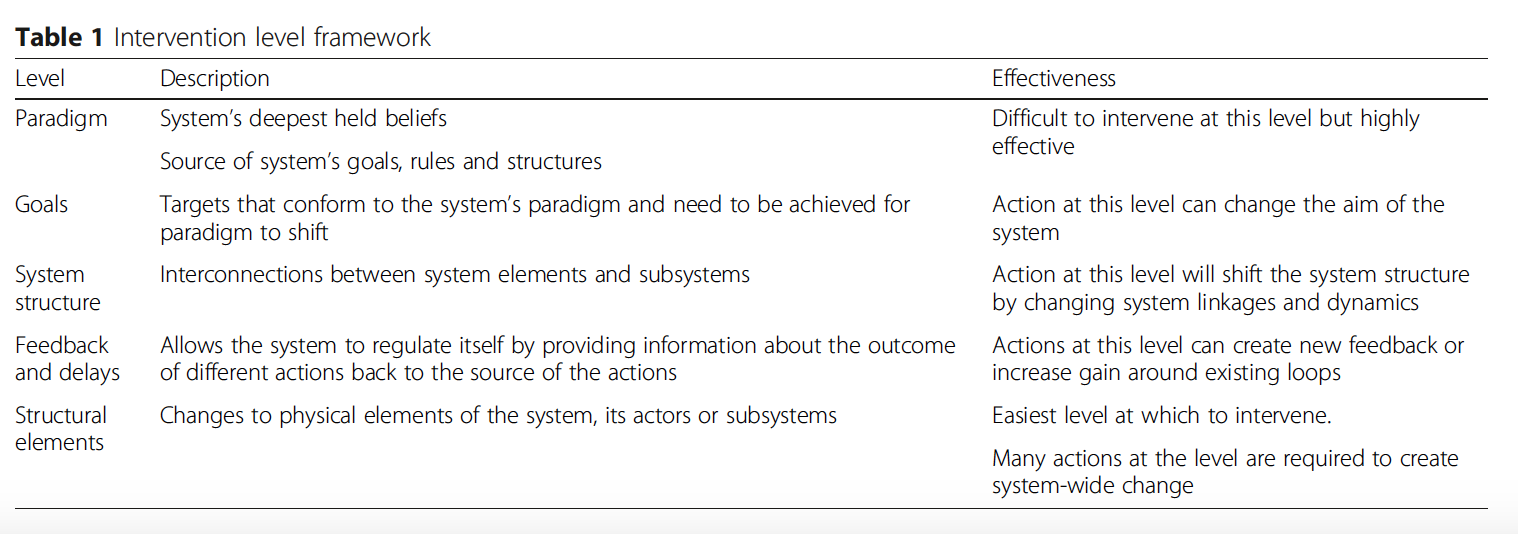 | More of a strategic view, than one focused on interventions. But helpful to recognise that the conceptualisation of 'structural' change that is encompassed by our recommendations are actually at the most superficial layer of this hierarchy |
| Hawkes, 2013 A food policy package for healthy diets and the prevention of obesity and diet-related non-communicable diseases: the NOURISHING framework | Presents the NOURISHING framework of food policies to promote healthy diets | Framework drawn from existing suggested strategies for improving the food environment. Interventions supported with selected (non-systematic) evidence in the text, but this is often supportive evidence from observational studies, rather than empirical evidence of intervention effectiveness. Many references are from WHO documents | Food Environment' N - Nutrition label standards and regulations O - Offer healthy foods and set standards in public institutions U - Use economic tools to address food affordability and purchase incentives R - Restrict food advertising and other forms of commercial promotion I - Improve the quality of the food supply S - Set incentives and rules to create a healthy retail environment (e.g. planning/licensing) 'Food System' H - Harness supply chain and actions across sectors 'Behaviour Change Communication' I - Inform people about food and nutrition through public awareness N - Nutrition advice and counselling in healthcare settings G - Give nutrition education and skills | Separation into food environment (what people are presented with), food system (what happens behind the scenes), and communication (what you tell people) may be less applicable to other risk factors  N, O, U, R all map well to the evidence collected I, S, H are considered to some degree but empirical evidence was found lacking I, N, G are mainly individual-level interventions |
| Hyseni, 2017 The effects of policy actions to improve population dietary patterns and prevent diet-related non-communicable diseases: scoping review | Scoping (umbrella) review of policy actions to improve diet | Framework itself drawn from pilot searches of the literature, building upon the 4P's of social marketing (price, place, promotion, and product). Followed up by search for the interventions themselves from identified reviews | Food price—policies influencing prices through taxes, subsidies or economic incentives; Food promotion—advertising/marketing; particularly on children; media campaigns and health education; Food provision—in specific settings: schools, communities or workplaces; Food composition—reformulation or elimination; Food labelling—nutrition labelling, calorie labelling in stores/restaurants; Food supply chain, trade and investment—including legislation or regulation affecting production policies or supply-chain logistics; Multi-component interventions (including at least two of the categories described above) | Price, promotion, provision*, composition, labelling all map well to evidence collected  Supply chain interventions not particularly well covered by evidence collected, and may reflect this being a higher level of the Carey systems framework. However, these interventions are considered to some degree by the evidence base (e.g. salt substitution of a village food chain)  *Evidence for setting-specific interventions generally supports only a small effect size being possible |
| Lloyd-Williams, 2014 Smorgasbord or symphony? Assessing public health nutrition policies across 30 European countries using a novel framework | Aimed to create a public health nutrition database, summarising current policies in 30 European countries, supported by qualitative interviews with policymakers to understand the level of implementation of the policies | "After extensive piloting and reviewing, we agreed the most practical and coherent approaches was the "4P's" marketing mix framework" | Price - taxes; subsidies; or other economic incentives Product - reformulation; elimination or new products Place - schools, workplaces or community settings Promotion - restricting marketing to children and adults (advertising controls); nutritional food labelling; nutritional information on menus; public information campaigns; and health education | Price, product, place*, promotion all map well to the evidence collected  *Evidence for setting-specific interventions generally supports only a small effect size being possible |
| Lloyd-Williams, 2021 The QUEST for Effective and Equitable Policies to Prevent Non-communicable Diseases: Co-Production Lessons From Stakeholder Workshops | Reports the learning from 4 policymaker and stakeholder workshops, trying to understand barriers to effective implementation of NCD prevention strategies | Not completely clear, but appear to have been derived from the interventions put forward within the workshops | Workshop 2 Life skills - e.g school readiness, cooking skills, leadership on mental health Active design - eg. active city planning Social policy - e.g. junk food sport sponsorship regulation, traffic light labelling, living wage Food policy - e.g. mandatory alcohol labelling, reshape agriculture Regulation of risk factors - e.g. ban marketing of unhealthy products, smoke free places Fiscal policies - e.g. junk food tax, free childcare  Workshop 3 CDOH - e.g. tobacco pricing policies Self-worth - e.g. upstream policy for improved population mental health Good places - e.g. urban design for active travel Income/Employment - e.g. minimum income, reduce food poverty Regulation of harmful substances - e.g. nationalisation of alcohol retail sales | Derived from suggested interventions, rather than set-out as a framework to help structure interventions. As a result, categories appear to be quite broad and not overly useful |
| Marteau, 2019 Increasing healthy life expectancy equitably in England by 5 years by 2035: could it be achieved? | Reports outcome from an expert meeting convened to provide advice to the UK Government on interventions to increase healthy life expectancy, and reduce health inequalities. Identifying the most promising population preventive interventions to add 5 years to healthy life expectancy by 2035, and reduce the gap between the rich and the poor in England | \| Unclear how the framework was selected. Participants were not requested to provide example interventions according to any framework, so framework may have been derived from the interventions proposed \| \| --- \| | 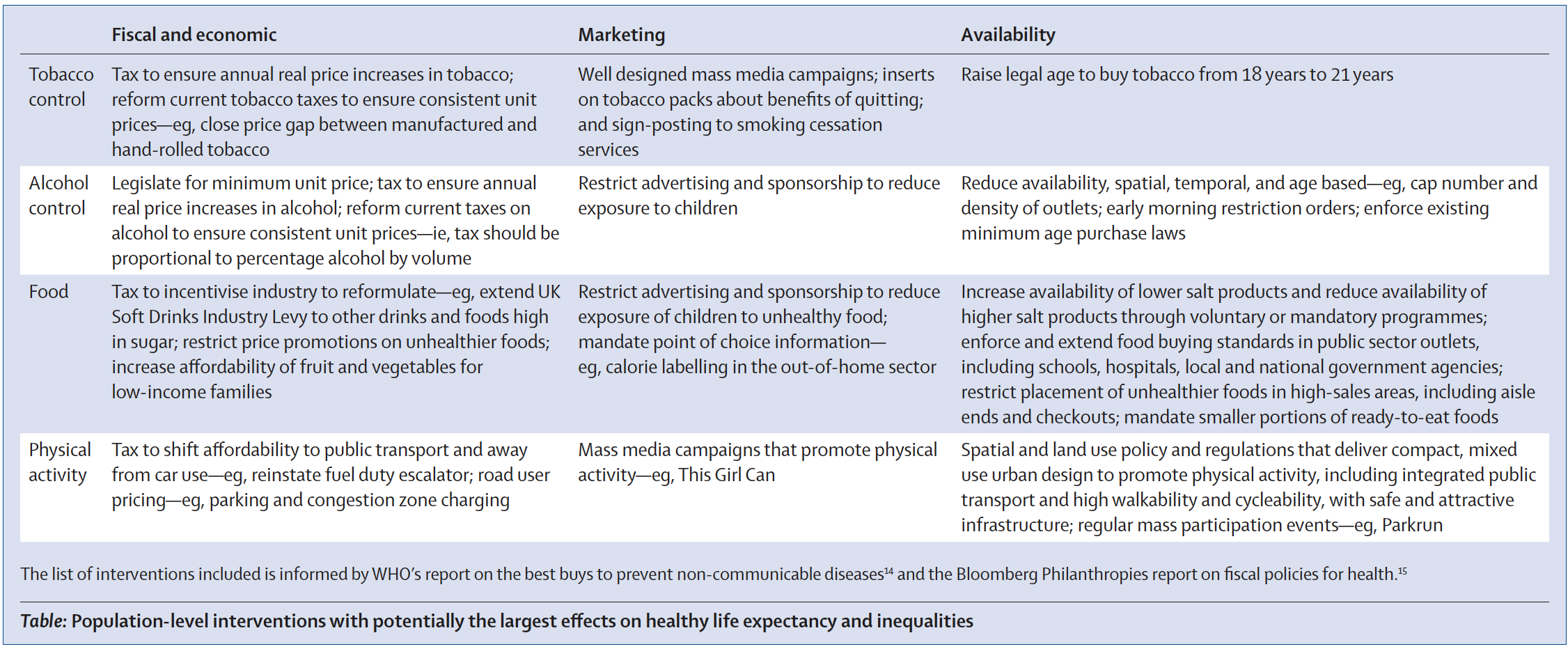 | Map well to the evidence collected. Note that many of the interventions ultimately considered to have sufficient evidence base for inclusion were based upon the evidence from the WHO Best Buys report |
| Watson, 2017 Priority actions for addressing the obesity epidemic in England | Application of the Food Environment Policy Index (EPI) to England, using government documents and checking accuracy with officials. Followed by an expert meeting to propose policies to fill identified gaps and set priorities for action | EPI was developed through an expert meeting | 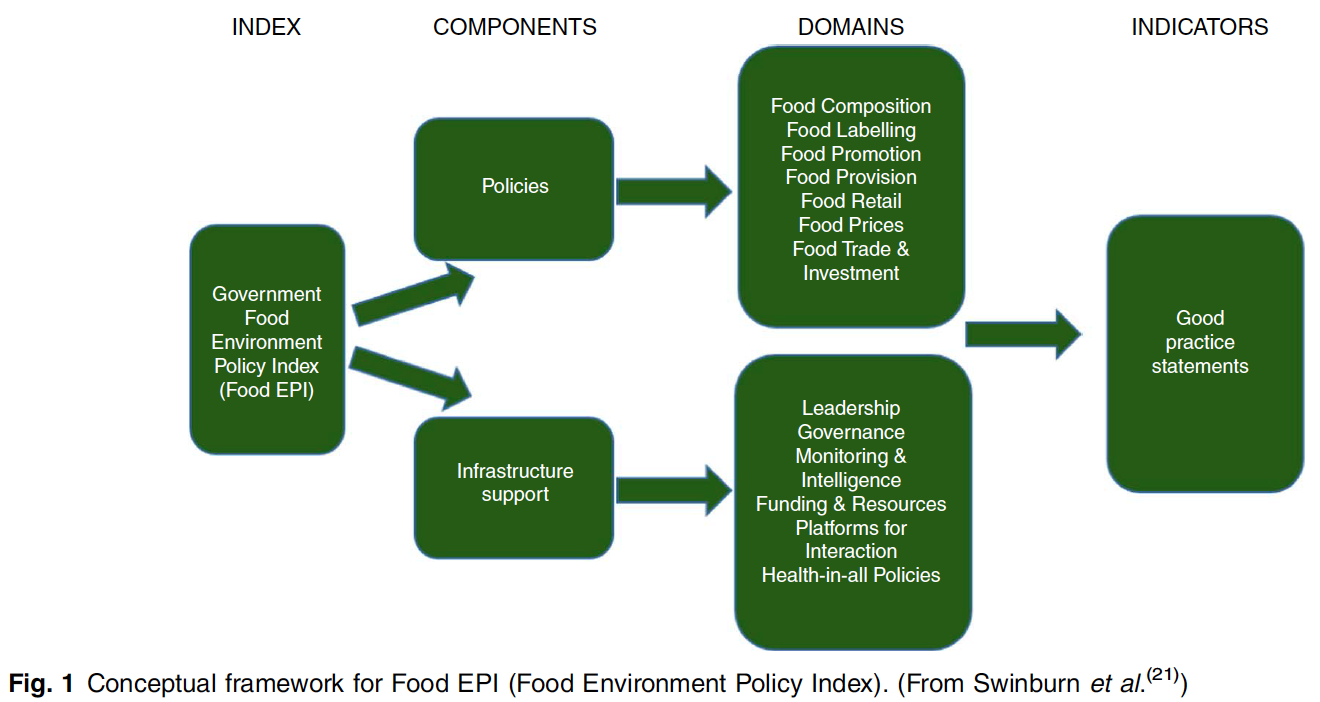 | The overall structure splits policies into those interventions we have considered, and 'infrastructure support' which speaks to Carey et al's hierarchy with tiers of strategic aims above our interventions  The interventions themselves (reformulation, labelling, marketing, provision, prices) all map well to the evidence collected, with the exception of "food trade and investment", which may reflect this being a higher level of the Carey et al. systems framework. However, these interventions are considered to some degree by the evidence base (e.g. salt substitution of a village food chain) |
| WHO, 2010 Global strategy to reduce the harmful use of alcohol | To outline the WHO 2010 global strategy to reduce alcohol-related harm | Unclear | (a) leadership, awareness and commitment (b) health services’ response (c) community action (d) drink-driving policies and countermeasures (e) availability of alcohol (f) marketing of alcoholic beverages (g) pricing policies (h) reducing the negative consequences of drinking and alcohol intoxication (i) reducing the public health impact of illicit alcohol and informally produced alcohol (j) monitoring and surveillance. | Specific intervention types for population-level primary prevention are: (e) availability of alcohol (f) marketing of alcoholic beverages (g) pricing policies  which map well to the evidence collected |
| WHO, 2022 A public health perspective on alcohol establishments: licensing, density and locations | Part of a series of advocacy briefs, to highlight attention to "blind spot" areas that have not been well implemented from the 2010 global action plan | \| Methods for the framework are not reported separately, but for the brief as a whole: "First, leading experts were engaged in searching and consolidating the available scientific evidence. Second, the first-hand experiences of countries related to the topic were sampled and documented. Third, stakeholders were brought together in webinars to discuss the evidence and country experiences. Lastly, the literature, experiences from countries and insights from discussions were brought together in a brief report that forms the varied issues of the “snapshots”. \| \| --- \| | 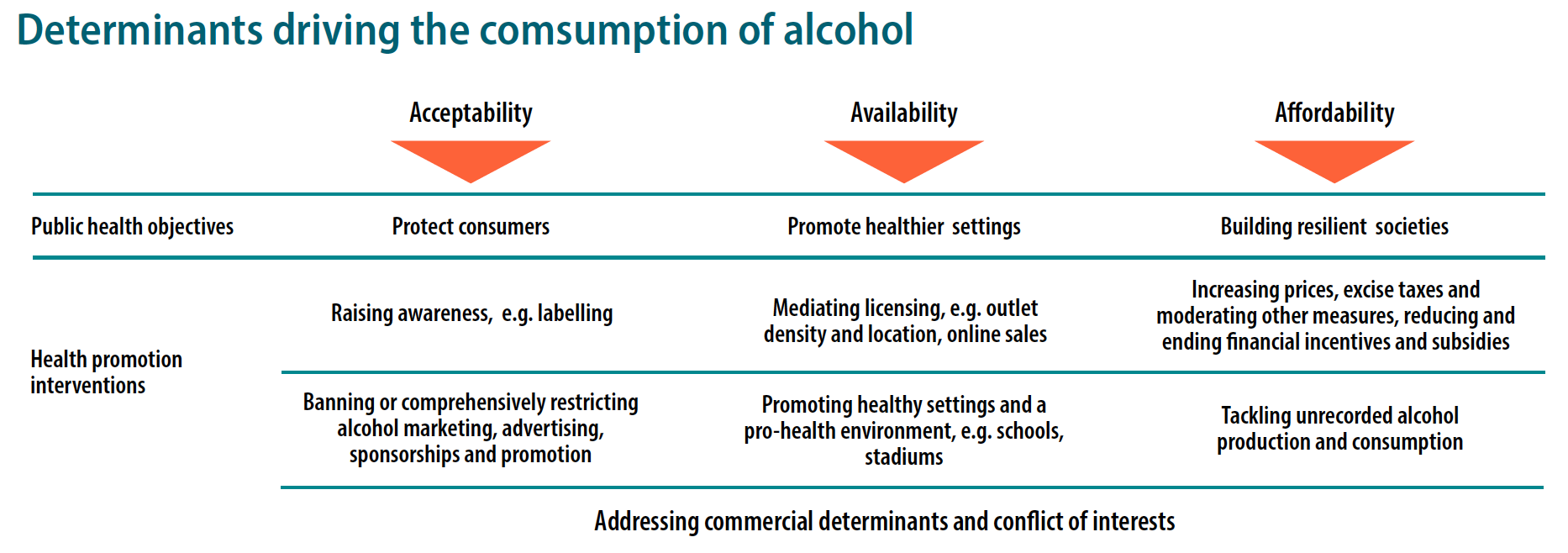 | This 3 A's framework maps well to the evidence collected, if a little narrow (possibly to fit the alliteration). For other risk factors, 'acceptability' may need to be broadened to definitively include all marketing restrictions, 'availability' may need to be widened to consider legislative interventions like reformulation, and 'affordability' to include interventions which place the financial burden on manufacturers rather than consumers |
